# Demethylating therapy increases anti-CD123 CAR T cell cytotoxicity against acute myeloid leukemia

**DOI:** 10.1038/s41467-021-26683-0

**Published:** 2021-11-08

**Authors:** Nadia El Khawanky, Amy Hughes, Wenbo Yu, Renier Myburgh, Tony Matschulla, Sanaz Taromi, Konrad Aumann, Jade Clarson, Janaki Manoja Vinnakota, Khalid Shoumariyeh, Cornelius Miething, Angel F. Lopez, Michael P. Brown, Justus Duyster, Lutz Hein, Markus G. Manz, Timothy P. Hughes, Deborah L. White, Agnes S. M. Yong, Robert Zeiser

**Affiliations:** 1 Precision Medicine Theme, South Australian Health and Medical Research Institute (SAHMRI), Adelaide, SA Australia; 2 School of Medicine, Faculty of Health and Medical Sciences, University of Adelaide, Adelaide, SA Australia; 3 Department of Medicine I, Medical Center - University of Freiburg, Faculty of Medicine, University of Freiburg, Freiburg, Germany; 4 Faculty of Biology, University of Freiburg, Freiburg, Germany; 5 Centre for Cancer Biology, SA Pathology and University of South Australia, Adelaide, SA Australia; 6 Department of Medical Oncology and Hematology, University Hospital Zurich and University of Zurich, Comprehensive Cancer Center Zurich (CCCZ), Zurich, Switzerland; 7 Institute of Experimental and Clinical Pharmacology and Toxicology, Division II, Faculty of Medicine, University of Freiburg, Freiburg, Germany; 8 Faculty of Medical and Life Sciences, University Furtwangen, Villingen-Schwenningen, Germany; 9 Department of Pathology, Institute for Clinical Pathology, University Medical Center Freiburg, Freiburg, Germany; 10 Department of Haematology, Royal Adelaide Hospital, Adelaide, SA Australia; 11 Cancer Clinical Trials Unit, Department of Medical Oncology, Royal Adelaide Hospital, Adelaide, SA Australia; 12 School of Biological Sciences, Faculty of Science, University of Adelaide, Adelaide, SA Australia; 13 Department of Haematology, Royal Perth Hospital, Perth, WA Australia; 14 School of Medicine, The University of Western Australia, Perth, WA Australia; 15 Signaling Research Centres BIOSS and CIBSS-Centre for Integrative Biological Signalling Studies, University of Freiburg, Freiburg, Germany

**Keywords:** Cancer immunotherapy, Acute myeloid leukaemia, Immunology

## Abstract

Successful treatment of acute myeloid leukemia (AML) with chimeric antigen receptor (CAR) T cells is hampered by toxicity on normal hematopoietic progenitor cells and low CAR T cell persistence. Here, we develop third-generation anti-CD123 CAR T cells with a humanized CSL362-based ScFv and a CD28-OX40-CD3ζ intracellular signaling domain. This CAR demonstrates anti-AML activity without affecting the healthy hematopoietic system, or causing epithelial tissue damage in a xenograft model. CD123 expression on leukemia cells increases upon 5′-Azacitidine (AZA) treatment. AZA treatment of leukemia-bearing mice causes an increase in CTLA-4^negative^ anti-CD123 CAR T cell numbers following infusion. Functionally, the CTLA-4^negative^ anti-CD123 CAR T cells exhibit superior cytotoxicity against AML cells, accompanied by higher TNFα production and enhanced downstream phosphorylation of key T cell activation molecules. Our findings indicate that AZA increases the immunogenicity of AML cells, enhancing recognition and elimination of malignant cells by highly efficient CTLA-4^negative^ anti-CD123 CAR T cells.

## Abstract (web summary)

The success of CAR-T cells for treating acute myeloid leukaemia (AML) is hampered by toxicity to normal cells and low CAR-T cell persistence. Here, the authors show that the demethylating compound 5′-Azacitdine increases anti-CD123 CAR-T cell cytotoxicity against AML.

## Introduction

Despite recent advances in the field of allogeneic hematopoietic cell transplantation (allo-HCT)^1^ and the introduction of disease-specific inhibitors (e.g., FLT3 inhibition)^2^, the prognosis of AML remains unfavorable in the majority of patients. While standard induction chemotherapy and allo-HCT can induce complete remissions, most patients eventually relapse and succumb to the disease^3^. Post-relapse treatment combinations with tyrosine kinase inhibitors (TKIs) and donor lymphocyte infusions (DLI) have led to long-term remission in a fraction of patients with FLT3-ITD AML^1,4^. In recent years, a plethora of immunotherapy-based treatment approaches has been developed. One of the most successful approaches uses T cells that express chimeric antigen receptors (CARs) where T cell specificity is re-directed towards cell surface antigens overexpressed on the cancer cell. Several CAR T cell products targeting the CD19 antigen on B cells are approved by the American Food and Drug Administration for relapsed or refractory B-lymphoid malignancies based on the high and durable clinical responses^5–7^.

Conversely, the use of CAR T cells against AML is more difficult due to undesired effects on normal myeloid progenitor cells that share most targetable antigens with AML cells. Of the antigens currently explored, CD123, the transmembrane α chain of the interleukin-3 receptor remains the most promising target because of its ubiquitous expression on AML blasts^8,9^. Several preclinical and clinical studies^10^ have reported potent anti-leukemic activity of anti-CD123 CAR T cells against AML. However, myelotoxicity or reduced CAR T cell efficacy has been reported due to the immunosuppressive AML microenvironment that hinders its broader applicability^10–12^.

Here, we used a third-generation anti-CD123 CAR product with the ScFv derived from the humanized and Fc optimized CSL362 monoclonal antibody (mAb)^13^. The anti-CD123 CAR T cells demonstrated anti-leukemic activity with no severe toxicity to the healthy hematopoietic system in vivo. To increase potency, combination therapy with the DNA methyltransferase (DNMT) inhibitor, 5′-azacitidine (AZA) was explored. Hypomethylating agents (HMAs) such as AZA are cytidine nucleoside analogs that induce transient and variable DNA hypomethylation. The resultant effect promotes reactivation of key epigenetically silenced genes thereby inducing senescence and apoptosis^14,15^. More importantly, AZA has been shown to upregulate the expression of leukemia-associated antigens on AML and other cancer cells. This activates a type I interferon (IFN) signaling response thereby increasing T cell efficacy^15^. However, AZA treatment alone is ineffective at eradicating leukemia stem cells in AML^16^. Most recently, AZA combined with the Bcl2 inhibitor, venetoclax, has proven to be an effective therapy for a proportion of AML patients^17,18^. Additionally, HMAs in combination with other agents, such as isocitrate dehydrogenase (IDH) or immune checkpoint inhibitors, are also under investigation^19–21^. Therefore, we proposed that combining AZA with anti-CD123 CAR T cells represents an attractive cellular therapy as both have demonstrated activity against AML and may present as a highly efficacious universal therapy for AML patients.

Here, we show that pre-treatment of AML cells with AZA followed by infusion of our third-generation anti-CD123 CAR T cells leads to long-term control of AML xenograft models. We report, here, that AZA treatment led to the upregulation of the target antigen, CD123. This resulted in reduced numbers of cytotoxic T lymphocyte antigen-4 (CTLA-4) expressing CAR T cells in vivo following infusion. CLTA-4 is a regulator of T cell activation and function, in which T cells with a low expression of this molecule showed increased longevity and activity^22^. AML-bearing mice infused with CTLA-4^negative^ anti-CD123 CAR T cells exhibited superior cytotoxicity against AML cells, with higher TNF-α production. TNF-α was also shown to be required for T cell-mediated cytotoxicity against tumor cells^23^. Furthermore, CTLA-4^negative^ anti-CD123 CAR T cells demonstrated proliferative capacity and longer survival compared to AML-bearing mice infused with CTLA-4^positive^ anti-CD123 CAR T cells.

Additionally, CD4^+^ anti-CD123 CAR T cells, exposed to AZA pre-treated AML cells, exhibited higher intracellular retention of CTLA-4 compared to naive AML cells. This observation was accompanied by higher phosphorylation levels of the T cell signaling and activation molecules, Lck and Zap70. We postulate that the increased immunogenicity of AZA pre-treated AML cells enhances the downstream signaling events of the CAR T cells by prolonging phosphorylation of molecules associated with T cell activation and function thereby preventing the induction of CTLA-4 on the surface of the CAR T cells. Our findings pave the way for a CAR T cell and AZA combination therapy for AML.

## Results

### Third-generation anti-CD123 CAR T cells exhibit anti-AML activity in vitro

In order to develop anti-CD123 CAR T cell therapy for AML, we first confirmed the expression of CD123 on primary human AML bone marrow (BM) versus healthy donor (HD) BM specimens (Supplementary Figs. 1 and 2a). Concordant with previous reports^24,25^, the median expression of CD123 on the AML specimens was significantly higher than on the HD specimens, where the overall expression was low (Supplementary Fig. 2b–d). While some AML samples had CD123^low^ expression, this was still higher than that of the HD cells.

We designed a third-generation anti-CD123 CAR construct incorporating the humanized CSL362-based^13^ ScFv fused to a CD28-OX40-CD3ζ intracellular signaling domain (Supplementary Fig. 3a). We used the lentiviral-based gene-transfer method to produce polyclonal CAR T cells that demonstrated the capacity to expand and differentiate into effector- or central-memory T cells (Supplementary Fig. 3b–d). Furthermore, expansion of the CAR T cells with recombinant human IL-2 did not induce high expressions of surface markers associated with activation and/or exhaustion including LAG-3, TIM-3, CTLA-4, and PD-1. This suggested that the possibility of the CAR T cells to be functionally inhibited is unlikely due to the low expression of these markers (Supplementary Fig. 3e).

The anti-CD123 CAR T cells demonstrated ability, in vitro, to express the T lymphocyte degranulation marker, lysosomal-associated membrane protein 1 (LAMP-1 or CD107a), proliferate, lyse target cells, and produce effector and homeostatic cytokines and chemokines against the human CD123^+^ cell line KG1a compared to the CD123^−^ cell line, SUPB15 (Supplementary Fig. 4). Furthermore, the cytotoxic activity of the CAR T cells against the MOLM-13 cells reached 90% at the 10:1 effector to target (E:T) ratio, while there was <10% cytotoxicity caused by the non-transduced (NTD) T cells at any E:T ratio (Supplementary Fig. 5a). More importantly, the high cytotoxic activity of the CAR T cells was observed against primary AML cells from 3 different patients with less than 10% cytotoxicity caused by the NTD T cells (Supplementary Fig. 5b). We next explored whether CAR T cells inhibit leukemia colony formation (CFU) of CD34^+^ and CD34^−^ BM-derived AML cells. We observed that colony formation was suppressed by CAR T cells and not NTD T cells (Supplementary Fig. 5c, d). These findings show the capacity of the third-generation anti-CD123 CAR T cells to induce robust anti-leukemic effects in vitro.

### Anti-CD123 CAR T cells exhibit in vivo activity against AML cells

**Fig. 1:**
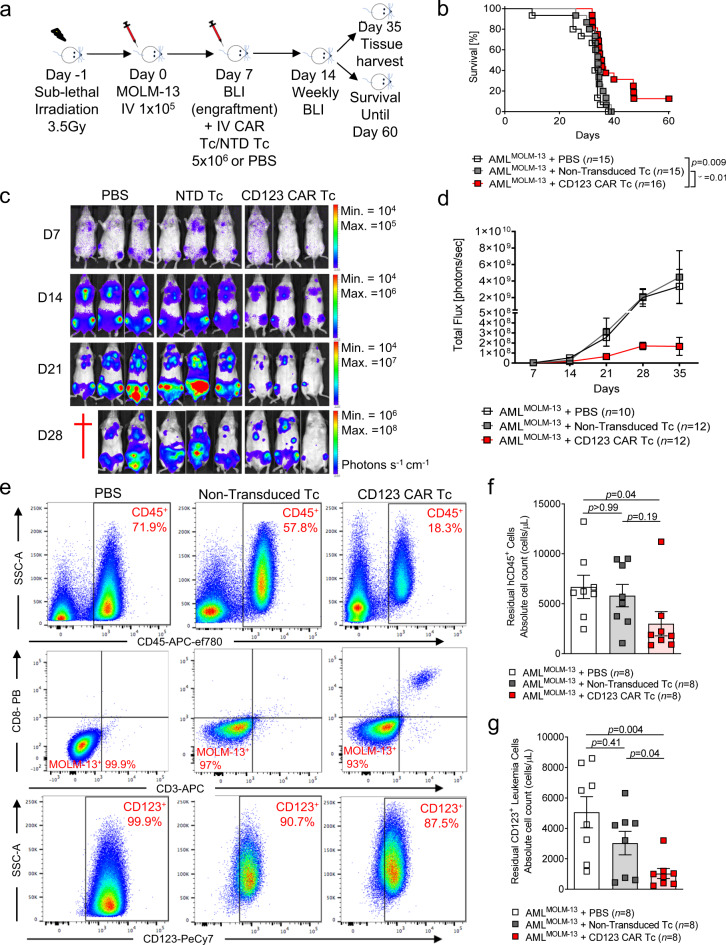
CD123 CAR T cells demonstrate anti-leukemic effects in MOLM-13 AML xenograft mice. **a** Schematic diagram summarizing the experimental plan for the MOLM-13^Luc^ AML xenograft model using *Rag2*^−/−^*Il2rγ*^−/−^ recipients. **b** Kaplan–Meier analysis demonstrating percentage survival of AML^MOLM-13^-bearing mice treated with PBS (*n* = 15), non-transduced T cells (NTD) (*n* = 15), or anti-CD123 CAR T cells (*n* = 16). Data were pooled from three independent experiments. *p-*value was calculated using the two-sided Mantel–Cox (log-rank) test. Attrition of mice was due to paralysis in both hind legs, growth of subcutaneous tumors (>2 cm), or BLI-detectable AML progression in the head. **c** Representative serial BLI images from three independent experiments depicting leukemia burden of MOLM-13 engrafted mice treated with PBS, NTD Tc or anti-CD123 CAR Tc. Data are represented colorimetrically (photons s^−1^ cm^−^^1^) with the scale bars indicating upper (max) and lower (min) BLI thresholds at each analysis time point. **d** Quantification of the BLI signal at the indicated time points for each treatment group. Data was pooled from two independent experiments, and represented as mean ± SEM for each treatment group (PBS *n* = 10; NTD Tc *n* = 12; anti-CD123 CAR Tc *n* = 12). On day 35, at least 5 mice per group were still alive and included in the analysis. **e** Representative flow cytometry-gating strategy for total human CD45, residual MOLM-13 CD123^+^ leukemia cells as well as residual CD4^+^/CD8^+^ T cells from the BM of mice treated with PBS, NTD Tc or anti-CD123 CAR Tc Flow cytometric analysis depicting absolute counts (cells/μL) of live residual **f** human CD45^+^ and **g** hCD45^+^ CD3^−^ CD123^+^ leukemia cells in the BM of PBS (*n* = 8), NTD Tc (*n* = 8) or anti-CD123 CAR Tc (*n* = 8) treated mice. Data were pooled from two independent experiments and represented as mean absolute cell counts ± SEM for each treatment group. *p-*values for **f** and **g** were calculated using two-sided one-way ANOVA (Kruskal–Wallis test with Dunn’s multiple comparisons).

We used an established xenogeneic MOLM-13 AML model^4^ to test the anti-AML in vivo activity of the CAR T cells. This model allowed us to measure AML cell expansion using bioluminescence imaging (BLI) (Fig. 1a). With this xenograft model, the mice that received CAR T cells exhibited a modest improvement in survival compared to the groups treated with phosphate buffered saline (PBS) or NTD T cells (Fig. 1b). The AML-derived BLI signal showed the typical distribution pattern of the leukemic cells with major infiltration in the BM and peripheral organs. Additionally, we observed that the AML-derived BLI signal was lower when the AML-bearing mice received the CAR T cells compared to mice that were PBS or NTD T cell treated (Fig. 1c, d). Additionally, flow cytometric analysis revealed lower absolute counts of residual human CD45^+^ (hCD45) in the CAR T cell-treated mice compared to PBS and NTD T cell-treated mice.

Within this population of cells, residual CD123^+^ leukemia cells were significantly lower in CAR T cell-treated mice compared to the other groups 35 days following tumor inoculation (Fig. 1e–g). Despite the anti-leukemic activity of the CAR T cells, complete eradication of the disease was not observed, indicating the need for a combinatorial therapeutic approach.

### Azacitidine increases CD123 expression on AML cells and enhances the anti-leukemic activity of CD123 CAR T cells in vivo

**Fig. 2:**
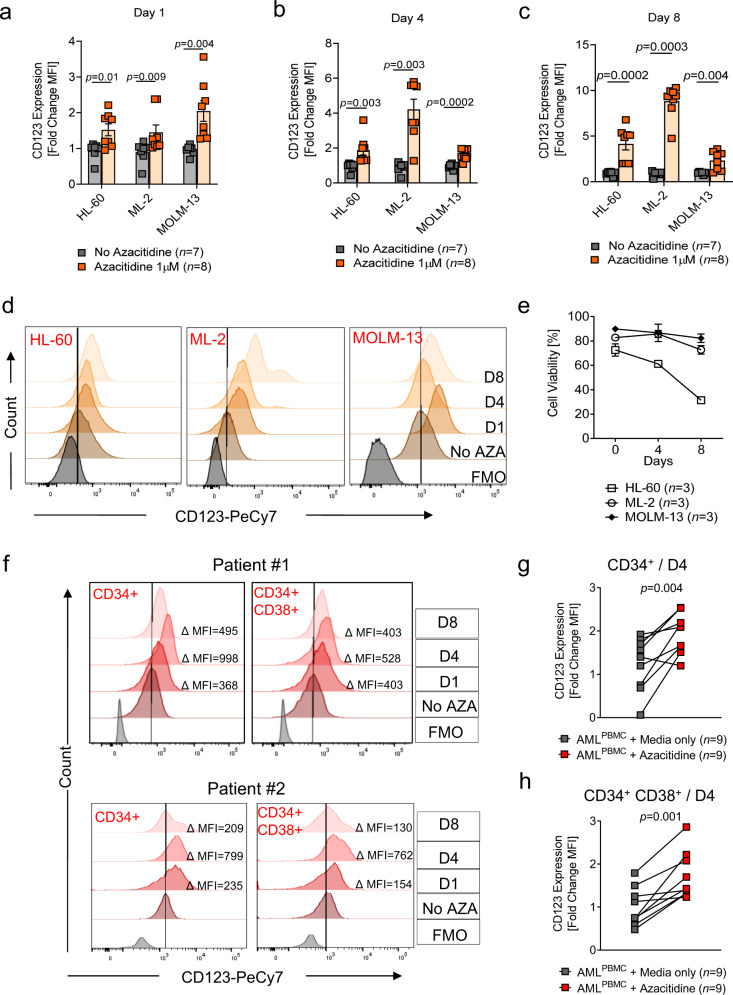
Azacitidine treatment leads to enhanced CD123 expression on AML cells. Scatter plot showing the expression of CD123 on HL-60, ML-2, and MOLM-13 cells represented as a fold change of mean fluorescence intensity (MFI) with respect to mean MFI of untreated controls (*n* = 7) on **a** day 1, **b** day 4, and **c** day 8 following culture with 1 µM azacitidine (AZA) (*n* = 8). *p*-values were calculated using a two-sided unpaired non-parametric student’s *t*-test (Mann–Whitney) or parametric student’s *t-*test. **d** Representative flow cytometric analysis of CD123 on AML cell lines (with varying basal CD123 expression levels) following culture in the absence or presence of 1 µM AZA for 1, 4, and 8 days. Histograms depict staining with anti-human CD123 Ab (PeCy7) compared to the fluorescence minus one (FMO) control. **e** Percentage cell viability (trypan blue) of HL-60 (*n* = 3), ML-2 (*n* = 3) and MOLM-13 (*n* = 3) cells at baseline (day 0) and upon exposure to 1 µM AZA after 4 days and 8 days. **f** Representative flow cytometric analysis of CD123 on 2 primary patient AML CD34^+^ and CD34^+^ CD38^+^ blast cells following culture in the absence or presence of 1 µM AZA for 1, 4, and 8 days. Histograms depict staining with anti-human CD123 Ab (PeCy7) compared to the FMO control. Inset numbers state the absolute difference in MFI between treated and non-treated cells. Scatter plot showing the expression of CD123 on primary AML **g** CD34^+^ and **h** CD34^+^ CD38^+^ cells represented as a fold change of MFI with respect to MFI of untreated controls (*n* = 9) on day 4 following culture with 1 µM AZA (*n* = 9). *p*-values were calculated using a two-sided unpaired student’s *t-*test (Mann–Whitney) or parametric student’s *t-*test. All graphed data in this figure are represented as mean values ± SEM.

**Fig. 3:**
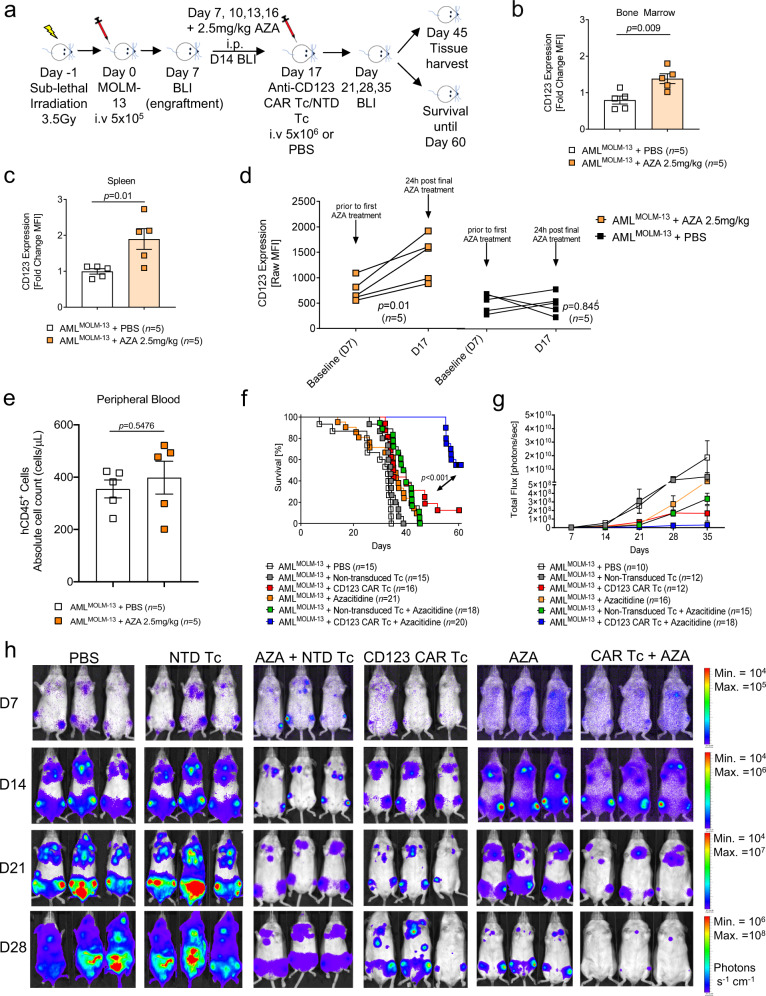
Azacitidine treatment supports the increased anti-leukemic effect of anti-CD123 CAR T cells in MOLM-13 xenograft mice. **a** Schematic diagram summarizing the experimental plan for the MOLM-13^Luc^ AML and AZA xenograft model using *Rag2*^−/−^*Il2rγ*^−/−^ recipients. Scatter plots showing the expression of CD123 at D17 in the **b** BM and **c** spleen, represented as a fold change of MFI of AML^MOLM-13^-bearing mice treated with 2.5 mg/kg AZA (*n* = 5) with respect to MFI of mice treated with PBS (*n* = 5). **d** Scatter plots showing the expression of CD123 at D17 in the PB, represented as raw MFI values of AML^MOLM-13^-bearing mice treated with AZA (*n* = 5) compared to mice treated with PBS (*n* = 5). **e** Scatter plots depicting the absolute cell count (cells/μL) of hCD45 in AML^MOLM-13^-bearing mice treated with AZA (*n* = 5) compared with AML^MOLM-13^-bearing mice treated with PBS (*n* = 5). Mice in **b**–**e** were pair-matched for similar hCD45 engraftment levels and CD123 MFI. **f** Kaplan–Meier analysis of percentage survival for each treatment group (PBS *n* = 15; NTD Tc *n* = 15; anti-CD123 CAR Tc *n* = 16; AZA *n* = 21; NTD Tc + AZA *n* = 18; anti-CD123 CAR Tc + AZA *n* = 20). Attrition of mice: as described above. All *p-*values were calculated with respect to the anti-CD123 CAR Tc + AZA group. **g** Quantification of the BLI signal for each treatment group over time (PBS *n* = 10; NTD Tc *n* = 12; anti-CD123 CAR Tc *n* = 12; AZA *n* = 16; NTD Tc + AZA *n* = 15; anti-CD123 CAR Tc + AZA *n* = 18). On day 35, at least 5 mice per group were still alive and included in the analysis. **h** Representative serial BLI from depicting leukemia burden in MOLM-13 engrafted mice treated with PBS, NTD Tc, anti-CD123 CAR Tc, AZA only, AZA with NTD Tc, and anti-CD123 CAR Tc + AZA. Data are represented colorimetrically (photons s^−1^ cm^−1^) with the scale bars indicating upper (max) and lower (min) BLI thresholds at each analysis time point. All graphed data are represented as mean ± SEM and pooled from two (**g**, **h**) or three (**b**–**e**) independent experiments. *p-*values were calculated using **f** two-sided Mantel–Cox test (log-rank) and **b**–**e** two-sided paired parametric student’s *t*-test.

**Fig. 4:**
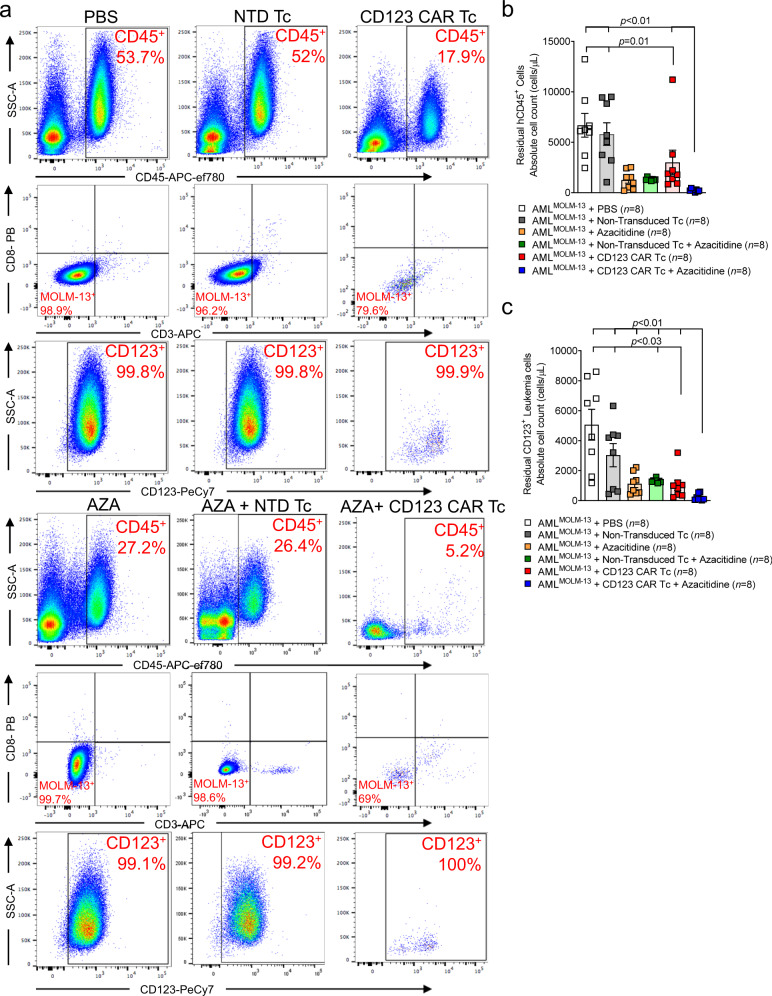
Azacitidine pre-treatment enhances anti-CD123 CAR T cell-mediated AML elimination. **a** Representative gating strategy used for flow cytometry-based analysis of total human CD45 cells, residual MOLM-13 CD123^+^ leukemia cells as well as residual CD4^+^/CD8^+^ T cells in the BM of mice treated with PBS, NTD Tc, anti-CD123 CAR Tc, AZA only, NTD Tc + AZA or anti-CD123 CAR Tc + AZA. Flow cytometric analysis depicting absolute cell counts (cells/μL) of residual **b** hCD45^+^ and **c** CD123^+^ MOLM-13 leukemia cells in the BM of the various treatment groups (PBS *n* = 8; NTD Tc *n* = 8; AZA *n* = 8; NTD Tc + AZA *n* = 8; anti-CD123 CAR Tc *n* = 8; anti-CD123 CAR Tc + AZA *n* = 8). All graphed data were pooled from two independent experiments and are represented as mean ± SEM. *p-*values were calculated using two-sided one-way ANOVA (Kruskal–Wallis test with Dunn’s multiple comparisons).

Since the anti-CD123 CAR T cell-treated mice did not eliminate the AML cells entirely, we sought a combination partner that would increase in vivo efficacy by upregulating the expression of CD123 on AML cells. Based on our knowledge of currently approved therapeutic agents for the treatment of high-risk myelodysplastic syndrome (MDS) and AML, we investigated the impact of the DNA methyltransferase inhibitor, AZA, on CD123 expression^16,26^. HL-60 and OCI-AML3 cells exhibited increased expression of CD123 and multiple immune response genes over an 8-day exposure to AZA as determined by RNA-seq (Supplementary Fig. 6a). Additionally, we observed a sustained increase of surface CD123 expression on the AML cell lines HL-60 and ML-2 and transient increases on MOLM-13 cells over an 8-day exposure to AZA (Fig. 2a–d). Cell viability did not decrease upon AZA exposure for ML-2 and MOLM-13 cells but was significantly decreased for HL-60 cells (Fig. 2e). CD123 expression was induced in some primary peripheral blood (PB) AML cells within 24 h of AZA administration. The overall peak increase in CD123 expression was observed on day 4 of exposure to AZA and decreased by day 8 in both bulk CD34^+^ and CD34^+^CD38^+^ blast populations (Fig. 2f–h). DNA methylome analysis revealed that AZA caused global DNA demethylation as well as demethylation at *cis-*regulatory elements (low methylated regions, LMR) or surrounding promoters (unmethylated regions, UMR) in MOLM-13 cells (Supplementary Fig. 6b–d). This effect remained detectable 48 h following depletion of AZA from the culture medium. The in vivo MOLM-13^Luc^ AML-bearing mouse model was designed to reflect the in vitro treatment schedule (Fig. 3a). We observed increased CD123 expression on the MOLM-13^Luc^ AML cells in mice treated with AZA on days 8, 17, and 23 compared to PBS-treated mice (Fig. 3b–d and Supplementary Fig. 6e, f). The AZA treatment did not reduce the number of viable CD45^+^ AML cells indicating no direct cytotoxic effect (Fig. 3e). Based on these observations, administration of CAR T cells within a 24 h time frame of the last AZA treatment is likely to be efficient. We intentionally did not treat the mice with CAR Tc and AZA simultaneously because AZA, when in direct contact with T cells, has been shown in several studies to induce T_regs_^27^, which could suppress the immune response against the leukemia cells. We observed that the AML-bearing mice that received AZA with CAR T cells exhibited superior survival compared to AZA alone, CAR T cells alone, or AZA with NTD Tc (Fig. 3f). We also observed that the AML-derived BLI signal was lowest in the AZA/CAR T cell combination group as compared to all other groups (Fig. 3g, h). The number of residual hCD45^+^ and hCD45^+^ MOLM-13 leukemia cells, found in the BM of mice that received AZA and CAR T cells, was significantly lower compared to the other treatment groups (Fig. 4a–c). These findings were recapitulated in a second AML mouse model using the human OCI-AML3 cell line (Supplementary Fig. 7a–e). Leukemia burden was lowest in the AZA/CAR T cell group and this group experienced the longest survival (Supplementary Fig. 7a–e).

### The AZA/anti-CD123 CAR T cell treatment combination does not cause inflammatory organ damage or hematopoietic insufficiency

One major concern of anti-CD123 CAR T cells is organ damage by activation of the endogenous T cell receptor and activity against the healthy hematopoietic cells that express CD123. We, therefore, analyzed different GVHD target organs^28,29^ as well as the ability of normal hematopoietic cells to form CFUs. We found no inflammatory organ damage in the colon, small intestine, and liver of the mice that received CAR T cells or the AZA/CAR T cell combination (Supplementary Fig. 8a–d). We did, however, observe leukemia cell infiltration in the liver of some mice treated with PBS or NTD T cells but not those treated with CAR T cells or the combination-treated mice (Supplementary Fig. 8e). This supports the concept that the CAR T cells are mainly activated via their CAR while activation via their endogenous TCR plays only a limited role.

**Fig. 5:**
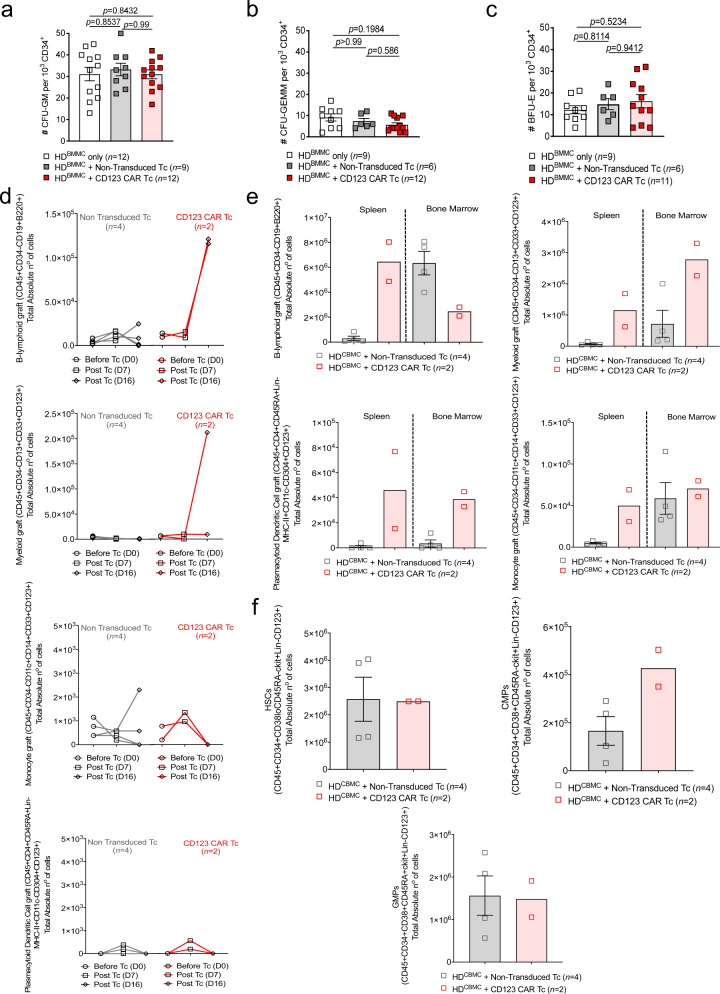
The effect of anti-CD123 CAR T cells on normal hematopoietic progenitor cell development. **a** CFU-GM (HD BMMC only *n* = 12; NTD Tc + HD BMMC *n* = 9; anti-CD123 CAR Tc + HD BMMC *n* = 12), **b** CFU-GEMM (HD BMMC only *n* = 9; NTD Tc + HD BMMC *n* = 6; anti-CD123 CAR Tc + HD BMMC *n* = 12), and **c** BFU-E (HD BMMC only *n* = 9; NTD Tc + HD BMMC *n* = 6; anti-CD123 CAR Tc + HD BMMC *n* = 11) colonies were scored using an inverted microscope following a 14-day culture in methocult supplemented with hSCF, IL-3, EPO, G-CSF, and GM-CSF. CD34^+^ HD BM mononuclear cells (BMMC) were cultured alone, with NTD T cells, or anti-CD123 CAR T cells at an E:T ratio of 1:1 for 6 h before the cell suspension was transferred and plated in methocult. Colony numbers are represented per 1000 plated cells. Data was pooled from three different primary HD samples, each plated in at least duplicates. **d** Flow cytometry-based analysis depicting the total absolute cell numbers of B-lymphoid (CD45^+^CD34^−^CD19^+^), Monocyte (CD45^+^CD34^−^CD14^+^CD16^+^CD33^+^/CD123^+^), Myeloid (CD45^+^CD34^−^CD13^+^CD33^+^/CD123^+^), and plasmacytoid dendritic cell (pDC) (CD45^+^CD34^−^CD4^+^CD45RA^+^CD11c^−^MHC-II^+^CD304^+^CD123^+^) graft in the PB of HD CD34^+^ cord blood (CBMC) engrafted NSG mice pre- (day 0) and post-infusion (day 7 and day 16) with 5 × 10^6^ NTD Tc (*n* = 4) or anti-CD123 CAR Tc (*n* = 2). **e** Flow cytometry-based analysis depicting the absolute cell numbers (cells/µL) of B-lymphoid, monocyte, myeloid, and pDCs in the spleen and BM of HD CD34^+^ CBMC NSG engrafted mice 16 days following infusion with 5 × 10^6^ anti- NTD Tc (*n* = 4) or anti-CD123 CAR Tc (*n* = 2). **f** Flow cytometry-based analysis depicting the absolute cell numbers (cells/µL) of hematopoietic stem cells (HSCs), common myeloid progenitors (CMPs), and granulocytic macrophage progenitors (GMPs) in the BM of HD CD34^+^ CBMC NSG engrafted mice 16 days following infusion with 5 × 10^6^ NTD Tc (*n* = 4) or anti-CD123 CAR Tc (*n* = 2). Data were pooled from two independent experiments (**d**–**f**) and all graphed data are represented as mean ± SEM. *p-*values were calculated using two-sided one-way ANOVA (Tukey’s test or Kruskal–Wallis test with Dunn’s multiple comparison) (**a**–**c**) or two-sided unpaired student’s *t*-test (Mann–Whitney) (**d**–**f**).

The expression of CD123 on several populations of normal hematopoietic cells, including circulating B cells, myeloid progenitors, dendritic cells, and megakaryocytes raises concerns regarding potential on-target, off-tumor effects. To analyze the potential hematopoietic toxicity of the anti-CD123 CAR T cells, we next exposed healthy BM cells to the CAR T cells and studied their ability to form CFU-GM, CFU-GEMM, and BFU-E colonies. We observed that the ability to form CFU-GM, CFU-GEMM, and BFU-E colonies was still intact in the CAR T cell-treated group versus medium only or NTD T cell-treated conditions (Fig. 5a–c).

To investigate the effect of the anti-CD123 CAR T cells on hematopoietic insufficiency in vivo, NSG and humanized cytokine knock-in *Rag2*^−/−^*Il2rγ*^−/−^ mice^30^ (MISTRG-SKI) were engrafted with HD CD34^+^ CB cells and treated with either anti-CD123 CAR or NTD T cells. Changes to the peripheral B-lymphoid, monocyte, myeloid, and plasmacytoid dendritic cells (pDC) graft were evaluated over 7 (MISTRG-SKI) or 16 days (NSG). Mice were terminated at days 14–16 and changes to the above-mentioned populations and the primitive hematopoietic populations were analyzed in the spleen and/or BM. We observed an increase in PB B-lymphoid and myeloid graft of anti-CD123 CAR T cell-treated NSG mice while there were no differences in the monocyte and pDC graft when compared to NTD T cell-treated mice (Fig. 5d). There were no significant differences observed in the PB between the groups in the MISTRG-SKI model (Supplementary Fig. 9a). While anti-CD123 CAR T cell-treated NSG mice revealed an increase in the spleen and BM in the majority of the target cell populations, this was not significantly different when compared to the NTD T cell control (Fig. 5e, f). Similarly, no differences were observed in any of the target cell populations between the MISTRG-SKI mice treated with anti-CD123 CAR T cells and NTD T cells in the spleen and BM (Supplementary Fig. 9b, c).

**Fig. 6:**
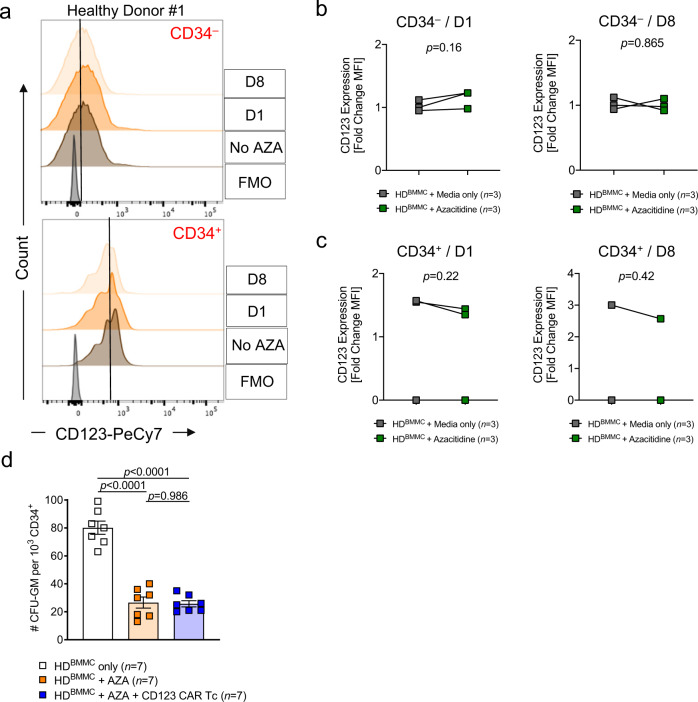
Impact of Azacitidine pre-treatment followed by anti-CD123 CAR T cell exposure on hematopoietic progenitor cell development. **a** Representative flow cytometry-based analysis of CD123 expression on CD34^+^ and CD34^−^ cells from a HD following culture in the absence or presence of 1 µM AZA for 1 or 8 days. Histograms depict staining with anti-human CD123 Ab (PeCy7) compared to the FMO control. Scatter plots showing the expression of CD123 on primary HD **b** CD34^−^ and **c** CD34^+^ cells represented as a fold change of MFI with respect to MFI of untreated controls (*n* = 3) on days 1 and 8 following culture with 1 µM AZA (*n* = 3). **d** CFU-GM colonies were scored using an inverted microscope following a 14-day co-culture in methocult supplemented with hSCF, IL-3, EPO, G-CSF, and GM-SCF. CD34^+^ HD BMMC was first co-cultured in the presence or absence of 1 µM AZA for 24 h. The following day the cells were washed. Untreated cells were cultured in media only (*n* = 7) and treated cells were cultured in media only (*n* = 7) or with anti-CD123 CAR Tc (*n* = 7) at an E:T ratio of 1:1 for 6 h. The cell suspension was subsequently transferred and plated in methocult. Colony numbers are represented per 1000 plated cells. Data were pooled from three primary HD samples, each plated in at least duplicates. All graphed data are represented as mean ± SEM. *p-*values were calculated using two-sided unpaired student’s *t*-test (Mann–Whitney) (**b**, **c**) and two-sided one-way ANOVA (Tukey’s test) (**d**).

Since AZA was found to increase the expression of CD123 on AML cells, it was important to evaluate whether the same pattern would be observed in healthy cells. We observed that the expression of CD123 on CD34^+^ and CD34^−^ HD BMMC was not significantly increased in the presence of AZA (Fig. 6a–c). In the clinic, AZA is reported to cause pancytopenia^31^. Concordant with published data^31^, we observed that AZA treatment significantly decreased the ability of the HD BMMC to form CFU-GM colonies compared to BMMC in medium only. However, the AZA/anti-CD123 CAR T cell combination did not further reduce CFU-GM colony formation compared to the AZA-only treatment (Fig. 6d). These findings indicate that the AZA/anti-CD123 CAR T cell combination is unlikely to cause inflammatory organ damage or profound hematopoietic insufficiency at a higher rate than AZA alone.

### AZA treatment induces a CTLA-4^negative^ CAR T cell fraction in vivo

**Fig. 7:**
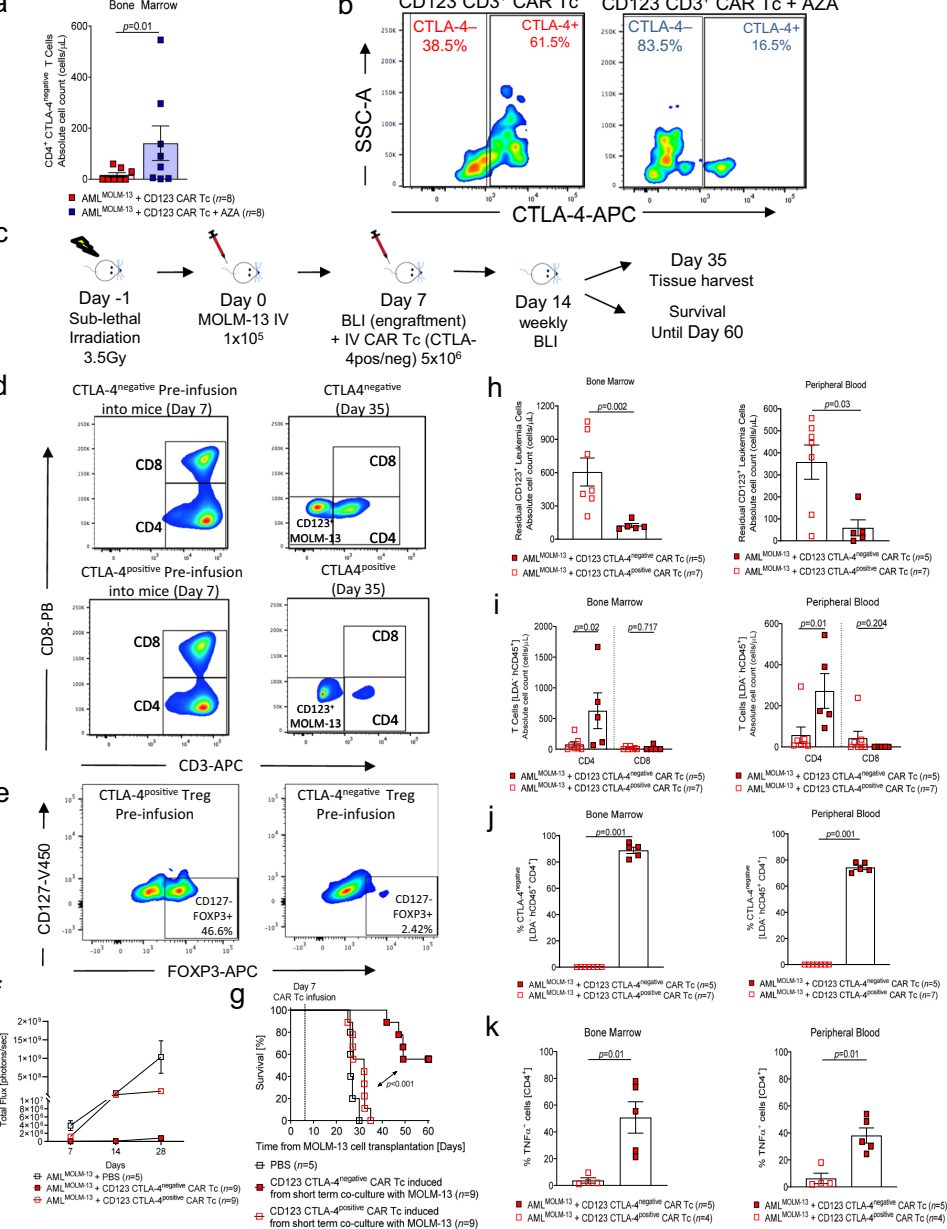
Azacitidine pre-treatment induces a population of cytotoxic CTLA-4^negative^ anti-CD123 CAR T cells in MOLM-13 xenograft mice. **a** Scatter plot demonstrating the residual BM isolated CD4^+^ CTLA-4^negative^ anti-CD123 CAR Tc of AML-bearing mice treated with anti-CD123 CAR Tc only (*n* = 8), or with AZA + anti-CD123 CAR Tc (*n* = 8). Data were pooled from two independent experiments. **b** Representative flow cytometry-gating depicting CTLA-4 expression on CD3^+^ T cells from mice treated with anti-CD123 CAR Tc only (left panel), or AZA + anti-CD123 CAR Tc (right panel). **c** Schematic diagram depicting the experimental plan for the MOLM-13^Luc^ AML model receiving CTLA-4^positive^ or CTLA-4^negative^ anti-CD123 CAR Tc using *Rag2*^−/−^*Il2rγ*^−/−^ recipients. **d** Representative flow cytometry plots depicting CD4^+^ and CD8^+^ cells of the CTLA-4^negative^ or CTLA-4^positive^ anti-CD123 CAR Tc pre-infusion versus the following infusion into the mice. **e** Representative flow cytometry plots depicting T_regs_ (CD4^+^CD25^hi^CD127^lo^FOXP3^+^) in the sorted CTLA-4^negative^ or CTLA-4^positive^ anti-CD123 CAR Tc, before infusion into the mice. **f** Summary BLI signals for each treatment group over time (PBS *n* = 5; CTLA-4^negative^ anti-CD123 CAR Tc *n* = 9; CTLA-4^positive^ anti-CD123 CAR Tc *n* = 9). **g** Kaplan–Meier analysis of percentage survival for each treatment group (PBS *n* = 5; CTLA-4^negative^ anti-CD123 CAR Tc *n* = 9; CTLA-4^positive^ anti-CD123 CAR Tc *n* = 9). Attrition of mice: as described above. Scatter plots showing absolute cell count (cells/μL) of **h** residual CD123^+^ leukemia cells and **i** residual CD4^+^ and CD8^+^ T cells in the BM and PB of mice treated with CTLA-4^negative^ (*n* = 5) or CTLA-4^positive^ anti-CD123 CAR Tc (*n* = 7). **j** Scatter plots depicting the percentage of CTLA-4^negative^ cells CD4^+^ T cells in the BM and PB of mice treated with CTLA-4^negative^ (*n* = 5) or CTLA-4^positive^ anti-CD123 CAR Tc (*n* = 7). **k** Scatter plots depicting the percentage of TNFα expression on CD4^+^ T cells in the BM and PB of mice treated with CTLA-4^negative^ (*n* = 5) or CTLA-4^positive^ anti-CD123 CAR Tc (*n* = 4). All graphed data, apart from survival analysis are represented as mean ± SEM. *p-*values were calculated using two-sided Mantel–Cox test (log-rank) (**g**) or two-sided unpaired student’s *t*-test (Mann–Whitney) (**f**, **h**–**k**).

To better characterize the mechanism by which AZA improves the in vivo efficacy of anti-CD123 CAR T cells, we next analyzed CAR T cells isolated from monotherapy treated mice or from the AZA/anti-CD123 CAR T cell-treated mice. In the MOLM-13^Luc^ AML-bearing mice, we observed that there was no difference in the number of residual CD4^+^ and CD8^+^ T cells in the BM and PB of mice (Supplementary Figs. 10a, b and 11a, b) or the number of residual CD4^+^ and CD8^+^ CAR T cells that were PD-1^negative^ TIM-3^negative^ between the treatment groups (Supplementary Figs. 10c, d and 11c, d). Interestingly, we found that CAR T cells isolated from AML-bearing mice that received prior AZA treatment exhibited a significantly higher residual number of CTLA-4^negative^ CD4^+^ anti-CD123 CAR T cells in the BM 28 days following anti-CD123 CAR T cell infusion, but not the PB (Fig. 7a, b and Supplementary Fig. 11e). Additionally, we observed a similar trend for CD8^+^ CAR T cells. However, these data were not significant (Supplementary Figs. 10e and 11f). Within the CD4^+^ and CD8^+^ T cell compartments, there were no consistent differences in the phenotypic distribution of T_scm-like_, T_cm_, T_em_, and T_eff_ cells between the two treatment groups in both BM and PB (Supplementary Fig. 12). Additionally, similar observations were made with the OCI-AML3 AML-bearing mice (Supplementary Figs. 13–15). However, in contrast to the MOLM-13 model, dual-treated mice showed a higher amount of residual PB CD4^+^ CAR T cells (Supplementary Fig. 14a). Higher numbers of CTLA-4^negative^ CD4^+^ T cells were also observed in both the BM and PB in the AZA/CAR T cell-treated group compared to the CAR T cell-only group (Supplementary Figs. 13e and 14e). Higher numbers of CTLA-4^negative^ CD8^+^ T cells were also found in the BM in the AZA/CAR T cell-treated group compared to the CAR T cell-only group (Supplementary Fig. 13f).

### CTLA-4^negative^ anti-CD123 CAR T cells allow for robust anti-leukemic efficacy in vivo

To functionally validate whether CTLA-4^negative^ anti-CD123 CAR T cells are responsible for sustained anti-leukemia effects and subsequently improved survival in AML-bearing mice, CTLA-4^negative^ or CTLA-4^positive^ CAR T cells were transferred into leukemia-bearing mice. In this model, the anti-CD123 CAR T cells were first exposed to MOLM-13 cells for 7 days, in vitro, to generate CTLA-4^negative^ and CTLA-4^positive^ populations. These populations were sort purified and injected into MOLM-13 engrafted mice on day 7 (Fig. 7c). Prior to the transfer of cells in the mice, we observed a 60:40 ratio of CD4:CD8 T cells in both CTLA-4^positive^ and CTLA-4^negative^ populations. Interestingly, in the BM and PB 28 days post CAR T cell infusion, the residual T cells were predominantly CD4^+^ T cells (Fig. 7d). T_regs_ were also analyzed in both CD4^+^ CTLA-4^positive^ and CTLA-4^negative^ anti-CD123 CAR T cells post-sorting. We found no T_regs_ in the sorted CD4^+^ CTLA-4^negative^ anti-CD123 CAR T cells and a moderate population in the CTLA-4^positive^ cells (Fig. 7e).

We found that the mice treated with CTLA-4^negative^ anti-CD123 CAR T cells demonstrated effective leukemia control whereas the mice treated with CTLA-4^positive^ anti-CD123 CAR T cells had exponential tumor progression (Fig. 7f). This observation was in agreement with an improved survival of mice injected with CTLA-4^negative^ anti-CD123 CAR T cells compared to mice injected with CTLA-4^positive^ CAR T cells (Fig. 7g). Flow cytometry-based analysis of the BM and PB 28 days following CAR T cell infusion also showed a significantly higher number of residual leukemia cells in mice injected with CTLA-4^positive^ anti-CD123 CAR T cells compared to the CTLA-4^negative^ group (Fig. 7h). The number of residual CD4^+^ T cells was significantly higher in the mice treated with CTLA-4^negative^ anti-CD123 CAR T cells compared to the mice treated with CTLA-4^positive^ anti-CD123 CAR T cells (Fig. 7i). Mice treated with CTLA-4^positive^ anti-CD123 CAR T cells remained positive for CTLA-4 in the BM and PB, whereas mice that had received CTLA-4^negative^ anti-CD123 CAR T cells had a high fraction of cells in both BM and PB that remained negative for CTLA-4 28 days following T cell injection (Fig. 7j). We also observed that mice treated with CTLA-4^negative^ anti-CD123 CAR T cells exhibited a higher expression of TNFα compared to the mice that were treated with CTLA-4^positive^ anti-CD123 CAR T cells (Fig. 7k). No residual CD8^+^ T cells were observed in either of the treatment groups. Additionally, no difference in the percentage of CTLA-4^negative^ or the expression of TNFα in CD8^+^ T cells was seen (Supplementary Fig. 16).

### CTLA-4^negative^ anti-CD123 CAR T cells exhibit recall immunity

**Fig. 8:**
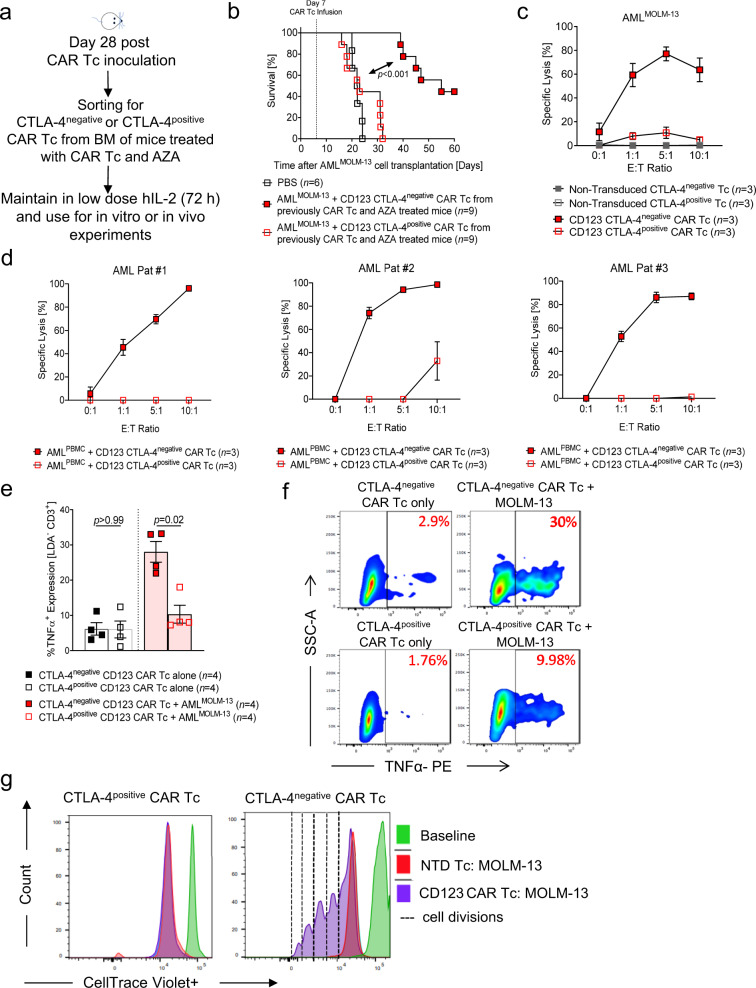
Recall immunity and long-term leukemia control. **a** Schematic diagram depicting the isolation of residual anti-CD123 CAR T cells from the BM or mice 28 days post inoculation for further in vitro and in vivo analysis. **b** Kaplan–Meier analysis of percentage survival for each treatment group PBS (*n* = 6; CTLA-4^negative^ anti-CD123 CAR Tc *n* = 9; CTLA-4^positive^ anti-CD123 CAR Tc *n* = 9). Secondary recipient mice were engrafted with 0.5 ×10^6^ MOLM-13^Luc^ cells on day 0. On day 7, mice were randomly distributed and received either 100 μL PBS, 2.5 ×10^6^ CTLA-4^positive^ or CTLA-4^negative^ anti-CD123 CAR Tc isolated from primary engrafted mice. Attrition of mice: as described above. **c** Specific cytotoxicity of MOLM-13 cells following co-culture with NTD or anti-CD123 CTLA-4^negative^ (*n* = 3) or CTLA-4^positive^ CAR Tc (*n* = 3). **d** Specific cytotoxicity of anti-CD123 CTLA-4^positive^ (*n* = 3) and CTLA-4^negative^ CAR Tc (*n* = 3) against 3 independent primary patient AML PB PBMCs with varying CD123^+^ expression following a 16 h co-incubation. For **c** and **d**, data represent three independent experiments with a fixed number of target cells/well for all E:T ratios. In both cases, counting beads were used to quantify the absolute number of residual live target cells. Residual live target cells were CellTrace violet^+^ 7-AAD^−^. **e** Scatter plot showing the percentage quantification of TNFα from live CTLA-4^negative^ or CTLA-4^positive^ CD3^+^ anti-CD123 CAR Tc following a 24 h co-culture with media only (*n* = 4) or MOLM-13 cells (*n* = 4). **f** Representative flow cytometry plot from each condition from **e** is shown. **g** Representative histograms of 3 independent experiments depicting the proliferation of CTLA-4^positive^ (left panel) and CTLA-4^negative^ (right panel) CD123 CAR T cells or NTD T cells as examined by CellTrace violet dye dilution. Cells were co-cultured for 96 h with MOLM-13 cells at an E:T ratio of 1:1. Each dotted line represents one cell division. All graphed data are represented as mean ± SEM. *p-*values were calculated using **b** two-sided Mantel–Cox test (log-rank) or two-sided unpaired student’s *t*-test (Mann–Whitney) (**e**).

An important feature of successful CAR T cell therapy is their capability to recall an immune response against the malignancy in the event of relapse. To determine the efficacy of CTLA-4^negative^ anti-CD123 CAR T cells derived from AZA-treated leukemia-bearing mice, we evaluated their immune recall capacity. CTLA-4^negative^ and CTLA-4^positive^ anti-CD123 CAR T cells were isolated 28 days following inoculation into primary recipients (Fig. 8a). Cells were maintained in low-dose IL-2 and re-inoculated into secondary MOLM-13 leukemia-bearing mice. CTLA-4^positive^ anti-CD123 CAR T cell-treated secondary recipients failed to recall an anti-leukemia immune response and did not survive longer than PBS-treated mice (Fig. 8b). Conversely, secondary recipients treated with CTLA-4^negative^ anti-CD123 CAR T cells demonstrated a significantly longer survival compared to mice treated with PBS or CTLA-4^positive^ anti-CD123 CAR T cells (Fig. 8b). In vitro, we subjected the sorted CAR T cells to MOLM-13 leukemia cells for 24 h and tested their cytolytic ability. We demonstrated that CTLA-4^negative^ anti-CD123 CAR T cells exerted high leukemia cell lysis, while CTLA-4^positive^ anti-CD123 CAR T cells exerted less than 10% killing of the MOLM-13 cells. The NTD T cells showed no killing capacity (Fig. 8c). The ability of the sorted CTLA-4^negative^ anti-CD123 CAR T cells to effectively kill leukemia cells was also seen using three independent patient-derived AML samples. No killing of the AML PBMCs was observed with the CTLA-4^positive^ anti-CD123 CAR T cells (Fig. 8d). We observed that the production of TNFα was significantly higher in the CTLA-4^negative^ anti-CD123 CAR T cells compared to the CTLA-4^positive^ anti-CD123 CAR T cells. (Fig. 8e, f). Additionally, the proliferation of CTLA-4^negative^ anti-CD123 CAR T cells in the presence of MOLM-13 cells was higher compared to CTLA-4^positive^ anti-CD123 CAR T cells or NTD T cells (Fig. 8g).

### CTLA-4^positive^ anti-CD123 CAR T cells do not negatively affect CTLA-4^negative^ anti-CD123 CAR T cells

To confirm that the increased cytotoxic immune response of the anti-CD123 CAR T cells is due to the change in the capacity of the AML cells to induce CTLA-4 expression on the T cells as a result of its exposure to AZA, CTLA-4^negative^, and CTLA-4^positive^ anti-CD123 CAR T cells, were separately co-cultured with MOLM-13 or OCI-AML3 AML cells that had been pre-treated with AZA for 8 days or with untreated cells. The observation that CTLA-4^negative^ anti-CD123 CAR T cells exhibit higher cytotoxicity in the presence of AZA pre-treated AML cells was made when using MOLM-13 and OCI-AML3 cells. CTLA-4^positive^ anti-CD123 CAR T cells were not able to specifically lyse untreated AML cells. However, the absent cytotoxic effect could, to a certain extent, be rescued when in the presence of AZA pre-treated AML cells (Supplementary Fig. 17a–d). CTLA-4^positive^ T cells are known to possess regulatory functions which could dampen the immune response. To understand whether the CTLA-4^positive^ anti-CD123 CAR T cells could exert cytotoxic effects and whether they could negatively affect the function of CTLA-4^negative^ anti-CD123 CAR T cells, CTLA-4^positive^, CTLA-4^negative^, or an equal amount of either cell phenotype were cultured with AML cells and the cytotoxic and proliferative capacity was investigated. CTLA-4^positive^ anti-CD123 CAR T cells did not affect the cytotoxic capacity of the CTLA-4^negative^ anti-CD123 CAR T cells when cultured with 3 different AML cell lines (Supplementary Fig. 18a–c). Similarly, proliferation was also unaffected when CTLA-4 ^positive^ and CTLA-4^negative^ CAR T cells were cultured together with the AML cells (Supplementary Fig. 18d–g).

### AZA primed leukemia cells support intracellular retention of CTLA-4 in CD4^+^ anti-CD123 CAR T cells and enhances proximal signaling

**Fig. 9:**
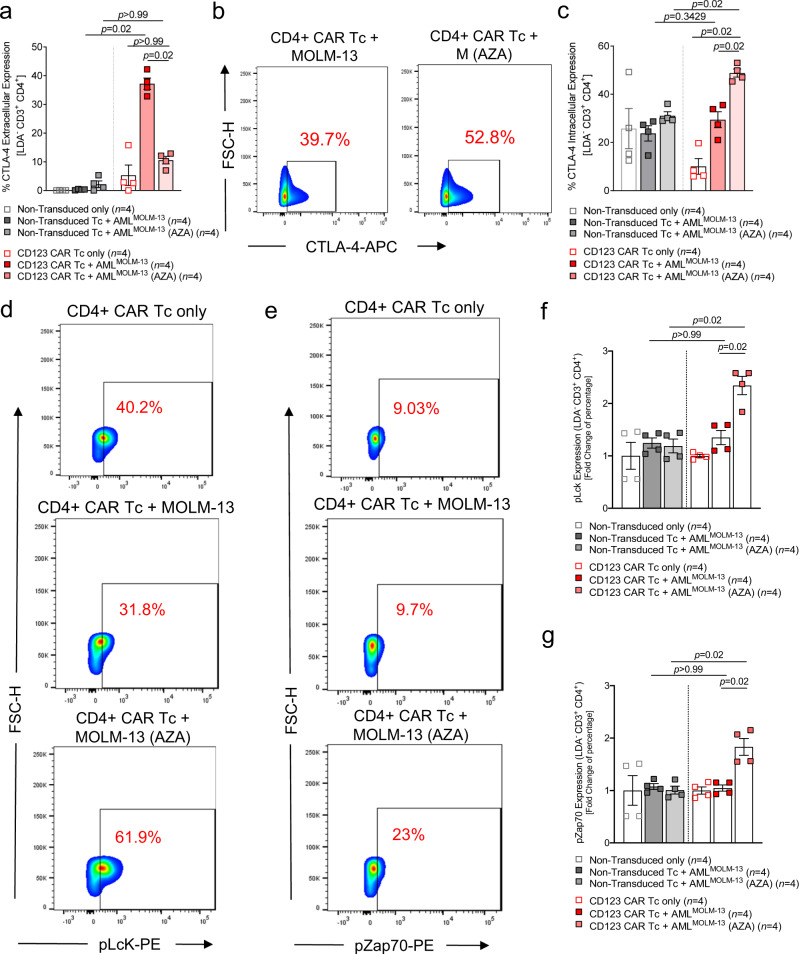
AZA pre-treatment of the leukemia cells promotes intracellular retention of CTLA-4 while enhancing Lck and ZAP70 signaling in the anti-CD123 CAR T cells. **a** Scatter plot showing the extracellular expression levels of CTLA-4 in CD4^+^ anti-CD123 CAR or NTD Tc following a 96 h co-culture in the presence of media only (*n* = 4), naive MOLM-13 cells (*n* = 4), or MOLM-13 cells pre-treated with 1 μM AZA (*n* = 4). Data were pooled from four independent experiments. **b** Representative flow cytometry plot depicting intracellular levels of CTLA-4 in CD4^+^ anti-CD123 CAR or NTD Tc. **c** Scatter plot showing the intracellular expression levels of CTLA-4 in CD4^+^ anti-CD123 CAR or NTD Tc following a 96 h co-culture in the presence of media only (*n* = 4), naive MOLM-13 cells (*n* = 4), or MOLM-13 cells pre-treated with 1 μM AZA (*n* = 4). Since raw intracellular expression levels could include surface levels of CTLA-4, extracellular and intracellular staining from the same sample were set up side by side and analyzed. The scatter plot depicts the difference between intracellular levels with extracellular levels to give the “true” intracellular expression of CTLA-4 in the cells. Data were pooled from four independent experiments. **d** Representative flow cytometry plot depicting the phosphorylated level of Lck (pLck) in CD4^+^ anti-CD123 CAR T cells that have been exposed for 96 h to media only, untreated MOLM-13 cells, or MOLM-13 cells pre-treated with 1 μM AZA. **e** Representative flow cytometry plot depicting the phosphorylated level of Zap70 (pZap70) in CD4^+^ anti-CD123 CAR T cells that have been exposed for 96 h to media only, untreated MOLM-13 cells, or MOLM-13 cells pre-treated with 1μM AZA. **f** Scatter plot depicting the fold change in percentage expression of pLck in CD4^+^ anti-CD123 CAR Tc (media only *n* = 4; untreated MOLM-13 *n* = 4; AZA-treated MOLM-13 *n* = 4). Data were pooled from four independent experiments. **g** Scatter plot depicting the fold change in percentage expression of pZap70 in CD4^+^ anti-CD123 CAR Tc (media only *n* = 4; untreated MOLM-13 *n* = 4; AZA-treated MOLM-13 *n* = 4). Data were pooled from four independent experiments and represented as mean ± SEM. *p-*values were calculated using two-sided one-way ANOVA (Kruskal–Wallis with Dunn’s multiple comparisons).

CTLA-4 is a surface receptor that mediates T cell responses. Prolonged extracellular expression is reported to inhibit T cell function by reducing intracellular downstream protein tyrosine phosphorylation of key signaling effectors such as zeta-associated protein of 70 kDa (Zap70) and p56 (Lck)^32^. We, therefore, sought to evaluate the extracellular and intracellular expression levels of CTLA-4 when CD4^+^ anti-CD123 CAR T cells were exposed to untreated or AZA pre-treated MOLM-13 cells. The CTLA-4 expression was compared to NTD T cells to discount possible expression as a result of TCR signaling. CD4^+^ anti-CD123 CAR T cells exposed to untreated MOLM-13 cells expressed significantly higher levels of surface CTLA-4 compared to CAR T cells exposed to AZA pre-treated MOLM-13 cells (Fig. 9a). Furthermore, the surface expression of CTLA-4 in the CAR T cells exposed to AZA pre-treated MOLM-13 cells was similar to that of resting CAR T cells cultured in media only. The opposite was observed with significantly higher intracellular levels of CTLA-4 when CD4^+^ anti-CD123 CAR T cells were cultured with AZA pre-treated MOLM-13 cells compared to untreated MOLM-13 cells (Fig. 9b, c). To understand whether the high expression of surface CTLA-4 was associated with an arrest in phosphorylation of Lck and Zap70, CD4^+^ and CD8^+^ anti-CD123 CAR T cells were co-cultured for 96 h in media, untreated MOLM-13 or AZA pre-treated MOLM-13 cells. CD4^+^ anti-CD123 CAR T cells demonstrated higher phosphorylation levels of Lck and Zap70 when cultured with AZA pre-treated MOLM-13 cells or naive MOLM-13 cells (Fig. 9d–g). This was similarly observed with CD8^+^ anti-CD123 CAR T cells for Lck but not Zap70 (Supplementary Fig. 19). These data indicate that mainly CTLA-4^negative^ CD4^+^ anti-CD123 CAR T cells are responsible for prolonged leukemia control and survival. Improved leukemia control is likely due to the ability of this population of CAR T cells to continuously produce TNFα and maintain phosphorylation of key intracellular molecules imperative for T cell activation and function.

## Discussion

The development of CAR T cells has caused a significant paradigm shift in the use of immunotherapeutic strategies for various malignancies. While its success has demonstrated improved survival outcomes for patients with certain B-lymphoid malignancies^5,6^, no comparable success has been achieved with CAR T cells directed against myeloid malignancies. The efficacy of CAR T cell therapy is reduced by the loss of target antigen which is associated with lack of persistence, due to the absence of activation. Both, antigen loss^33^ and failure of persistence of the CAR T cell population^34^ have been described. The clinical translation of CAR T cell therapy for AML has been further hindered by the development of an immunosuppressive microenvironment as well as on-target off-tumor toxicities. In this study, we used AZA to test if this was associated with increased CAR T cell efficacy.

To achieve this, we used third-generation anti-CD123 CAR T cells with a humanized CSL362-based^13^ ScFv and a CD28-OX40-CD3ζ signaling domain that exhibits anti-leukemic efficacy in vitro and in vivo. While the CAR T cells demonstrated anti-leukemic activity, disease eradication in the mice was incomplete. Therefore, we developed an approach to augment the expression of the target antigen, CD123, by pre-treating AML cells or leukemia-bearing mice with AZA. It is well documented that hypomethylating agents play a role in the prolonged survival of patients with myelodysplastic syndromes and relapsed/refractory AML by increasing the immunogenicity of tumor cells thereby enhancing T cell-mediated responses^35^. We found that the pre-treatment of leukemia-bearing mice with AZA followed by infusion of anti-CD123 CAR T cells led to improved AML rejection in vivo, which was associated with an improved survival of the mice.

We hypothesized that increased expression of CD123 on the AML cells would promote the expansion of functionally superior CAR T cells in vivo. Consistent with this concept, we found that pre-treatment of AML cells with AZA resulted in higher absolute numbers of CD4^+^ CTLA-4^negative^ CAR T cells compared to the CAR T cell only treated group. Functionally, these CTLA-4^negative^ CAR T cells were more potent compared to CTLA-4^positive^ CAR T cells in vitro with respect to their cytotoxicity against leukemia cells. Additionally, we also observed increased AML control in vivo which was associated with increased TNFα production. TNFα was shown to play a major role in leukemia control after allo-HCT^36^. Moreover, CTLA-4^negative^ CAR T cells were able to demonstrate continuous immune response in secondary AML-bearing mice while CTLA-4^positive^ CAR T cells did not. This finding is consistent with reports showing that the negative regulator of T cell activation, CTLA-4^37^, is mainly expressed on less effective T cells^22,38,39^. So far, no report had shown that the fraction of CTLA-4^negative^ anti-CD123 CAR T cells can be increased indirectly by AZA pre-treatment of the AML-bearing mice. In previous studies, AZA has been found to reduce the effects of DLI by promoting inhibitory T_reg_ cells while diminishing CD8^+^ effector T cell numbers^27^. Because of these previous observations, we avoided a direct contact of AZA to the transferred CAR T cells but only used an AZA pre-treatment regimen inducing CD123 on leukemia cells.

Enhanced expression of PD-L1, PD-L2, PD-1, and CTLA-4 in myelodysplastic syndromes upon treatment with hypomethylating agents has been reported^40^. Conversely, hypomethylating agents were also shown to induce an interferon response in cancer via dsRNA derived from endogenous retroviruses^15,41^. This indicates that hypomethylating agents can cause both, upregulation of inhibitory molecules and pro-inflammatory effects. The combination of AZA and SL-401 (tagraxofusp), the anti-CD123-directed cytotoxin, which consists of recombinant interleukin-3 fused to a truncated diphtheria toxin, is currently being tested in an ongoing clinical trial (ClinicalTrials.gov Identifier: NCT03113643) for patients with MDS or AML. Also consistent with our results, the combination of tagraxofusp and AZA was effective in patient-derived xenografts^42^. Consistent with our findings in AML, pre-treatment of CD19^+^ acute lymphoblastic leukemia (ALL) cells with AZA also showed enhanced CAR T cell efficacy^43^.

Further analysis revealed that exposure of leukemia cells to AZA caused immediate downstream consequences of antigen recognition strengthened CD3ξ ITAM phosphorylation. Previous studies have demonstrated that phosphorylation of CD3ξ occurs at a much greater intensity in CD28 co-stimulatory containing CARs due to the proline-rich region with which Lck associates^44,45^. In the presence of low antigen levels, inefficient Lck-mediated ITAM phosphorylation and Zap-70 activation can limit CAR T cell anti-leukemic responses^46^, while recent data show that non-canonical binding of Lck to CD3ε promotes TCR signaling and CAR function^47^. We observed that pre-treatment of leukemia cells with AZA was connected to increased Lck and Zap70 phosphorylation in CD4^+^ CAR T cells upon contact with the pre-treated leukemia cells. It is therefore possible that a higher expression of the target antigen CD123 may be one of the contributing mechanisms that lead to a stronger CAR activation and a consecutively higher level of pZAP70 in the CAR T cells. It is well known that AZA causes epigenetic changes to a wide array of genes. In this case, there may be other possible mechanisms contributing to the increased CAR T cell efficacy which were not further interrogated, in detail, in this study. For instance, Xu and colleagues^43^ demonstrated that the observed increase in CAR T cell efficacy may be due to upregulation of OX40L (*TNFSF4*) on the ALL cells. We also observed that AZA increased OX40L on the AML cells in our methylation and transcriptome studies. Since the CAR construct used in this study incorporates an OX40 co-stimulatory molecule, it is possible that increased signaling events through this interaction could also contribute to the enhanced CAR T cell efficacy.

There are conflicting reports showing that targeting of CD123 causes myelosuppression of healthy cells, which may be due to the different CAR constructs in use. We observed that the anti-CD123 CAR construct we used did not affect the viability and frequency of CFU-GM, CFU-GEMM, and BFU-E colonies in vitro. This was further shown in both a humanized NSG and MISTRG-SKI mouse model. These findings are concordant with others^10,42,48^. A recent study demonstrated in humanized mouse models that a third-generation construct comparable to our construct, namely CD28/4-1BB CAR T cells targeting CD123, exert no major cytotoxicity against various subsets of normal cells with low CD123 expression, indicating a low on-target/off-tumor toxicity effect^48^. Another study on a patient with a blastic plasmacytoid dendritic cell neoplasm enrolled in a clinical trial of anti-CD123 CAR T-cell therapy (ChiCTR1900022058) showed activity of the CAR T cells without significant hematotoxicity^49^.

In contrast, other studies have shown myelosuppression by CD123-directed CAR T cells^24,50^. The variances observed across the studies could be due to the different structural designs of the CAR. Nevertheless, recent studies have shown that attractive tumor target antigens that are also shared on healthy tissues can be “affinity-tuned” in order to avoid significant hematotoxicity^51^. We further confirmed that the increase in CD123 expression on AML cells was not observed in healthy hematopoietic cells under AZA induction. Additionally, the combination therapy did not significantly affect the ability of healthy CD34^+^ cells to form CFU-GM colonies which demonstrates that the addition of AZA into the treatment regimen would not bring any additional toxicities.

Besides myelosuppression, there is concern that the transfer of allogeneic CAR T cells may induce inflammatory organ damage or GVHD. Our preclinical studies found no evidence of TCR engagement from allogeneic transferred CAR T cells alone or combined with AZA as we did not observe any major tissue damage in GVHD target organs. In line with these findings, Ghosh et al.^52^ similarly reported that CAR T cells harboring the CD28 co-stimulatory motif demonstrated significantly decreased occurrence of GVHD compared to CAR T cells with the 4-1BB co-stimulatory motif. The combination of AZA and immunotherapy for AML is attractive and has already been tested, e.g., with immune checkpoint inhibition^21^ and DLIs with AZA for AML relapse after allo-HCT^31,53,54^. In contrast to the potential of polyclonal DLI to recognize major or minor MHC mismatches on leukemia cells, CAR T cells rely on the recognition of a defined target antigen which makes this approach more specific. Our study is the first report on a combination of AZA and CAR T cells for AML.

In summary, our studies show that AZA treatment increases expression of the CAR target, CD123, suggesting one possible mechanism attributing to improved leukemia control in vivo. The treatment induced higher numbers of CTLA-4^negative^ anti-CD123 CAR T cells which exhibited superior cytotoxicity compared to CTLA-4^positive^ anti-CD123 CAR T cells against AML target cells in vitro and in vivo. More importantly, these CTLA-4^negative^ CD123 CAR T cells exhibited prolonged phosphorylation of key intracellular markers important for cell activation and function thereby demonstrating capacity for immune memory in secondary non-AZA-treated AML-bearing mice. Since the combination of AZA with anti-CD123 CAR T cells did not cause epithelial tissue damage or significant hematopoietic insufficiency, our findings pave the way for a clinical trial combining AZA and anti-CD123 CAR T cells for AML treatment.

## Methods

### Human samples

Primary AML and healthy donor (HD) bone marrow (BM) samples were obtained from the South Australian Cancer Research Biobank (SACRB). Primary AML and HD peripheral blood (PB) was obtained from the University Medical Center, Freiburg, Germany. Written informed consent was obtained from each donor. HD cord blood (CB) samples were obtained from umbilical cords of healthy full-term newborns and processed by the biobank of the Department of Medical Oncology and Hematology, Switzerland, University Hospital Zürich. The studies were approved by the Australian Institutional Human Research Ethics Committee, the Institutional Ethics Review Board of the Medical Center, University of Freiburg, Germany, and the Cantonal Ethics Board Zürich, Switzerland (Ethics approval numbers: R20150526, HREC/15/RAH/221 and 509/16, and 2009-0062, respectively) and conducted in accordance with the Declaration of Helsinki.

### Mice

*Rag2*^–/–^*Il2rγ*^–/–^ immunocompromised mice were bred in-house at the University clinic (University of Freiburg) animal facility under standard room temperatures (~26 °C) with a 12 h light/dark cycle and humidity ranging 40–60%. Mice were housed in individually ventilated cages and received acidified and autoclaved water. Male and female mice were used between 8–10 weeks of age. Animal protocols were approved by the animal ethics committee Regierungspräsidium Freiburg, Freiburg, Germany (G18-019) or the Cantonal Veterinary Office Zürich (194/2018).

### MOLM-13 AML^FLT3 ITD^ xenograft model

For this model, *Rag2*^–/–^*Il2rγ*^–/–^ recipients were transplanted with 0.1 × 10^6^ MOLM-13^Luc^ cells intravenously following sub-lethal irradiation with 3.5 Gy 1 day prior. Mice were subsequently left for 7 days for leukemia to develop. Successful engraftment was confirmed on day 7 using in vivo bioluminescence imaging (BLI) before mice were distributed randomly into each treatment group. For in vivo BLI^55^ on day 7, mice were intravenously injected with 100 µL phosphate-buffered saline (PBS), 5 × 10^6^ non-transduced (NTD) T cells in 100 µL PBS, or 5 × 10^6^ anti-CD123 CAR T cells in 100 µL PBS. BLI was performed at weekly intervals to track leukemia progression or regression. Twenty-eight days following T cell injection, mice were either sacrificed for peripheral blood (PB) and bone marrow (BM) analyses by flow cytometry for the detection of residual AML cells (hCD45^+^ CD3^−^ CD123^+^) and T cells (hCD45^+^ CD4^+^/CD8^+^), or mice were left until day 60 for survival analysis (see schematic in figures).

### MOLM-13 AML^FLT3 ITD^ xenograft model with azacitidine treatment

For this model, *Rag2*^*–/–*^*Il2rγ*^*–/–*^ recipients were transplanted as above. Following confirmation of engraftment on day 7, mice were randomized and split into six groups: (1) PBS, (2) NTD T cells, (3) anti-CD123 CAR T cells only, (4) azacitidine (AZA) only, (5) AZA with NTD T cells, and (6) AZA with anti-CD123 CAR T cells. Mice in the PBS control group were treated intravenously with 100 µL PBS; mice in the anti-CD123 CAR T cells or NTD T cells only group were intravenously injected with 5 × 10^6^ anti-CD123 CAR T cells or NTD T cells, respectively. The AZA only, AZA with NTD T cells, and AZA with anti-CD123 CAR T cell treatment groups were first given one dose of 2.5 mg/kg AZA (Sigma Aldrich, Germany) in sterile PBS intraperitoneally every 3 days for a total of 4 doses. Twenty-four hours following the final dose of AZA, the dual treatment group was intravenously injected with 5 × 10^6^ NTD T cells or anti-CD123 CAR T cells in 100 µL PBS. A cohort of the mice from each group was sacrificed 28 days following T cell injection for flow cytometry analysis. The remaining cohort of mice from each group was left until day 60 for survival analysis (see schematic in figures).

### Primary healthy donor (HD) and AML cells

Primary cells were maintained in RPMI-1640 supplemented with 20% fetal calf serum (FCS), 2mM l-glutamine, 100 U/mL penicillin/streptomycin, and human stem cell factor (5 ng/mL; Peprotech, Rocky Hill, NJ).

### Peripheral blood mononuclear cell (PBMC) preparation

PBMCs were isolated by density gradient centrifugation using Lymphoprep or Pancoll (STEMCELL Technologies, Vancouver, BC, Canada; Pan Biotech, Germany). Briefly, blood samples were diluted 1:3 with 1× PBS, layered onto Lymphoprep/Pancoll, and centrifuged (30 min, 440×*g*) without brake. PBMCs were isolated and cryopreserved in 90% heat-inactivated FCS (Sigma Aldrich, Germany) and 10% dimethyl sulfoxide (DMSO) (Sigma Aldrich, Germany). Cells were stored overnight at −80 °C and subsequently transferred into liquid nitrogen. All analyses were conducted on cryopreserved material. Following thawing of sample material, cells were manually counted using trypan blue (Sigma Aldrich, Germany) staining.

### Tumor cell lines

The human leukemia cell lines MOLM-13, OCI-AML3, ML-2, HL-60, KG1a, and SUPB15 were purchased from ATCC (American Type Culture Collection, Manassas, Virginia, USA) and cultured in RPMI-1640 supplemented with 10% FCS, 2mM l-glutamine, 100 U/mL penicillin/streptomycin. MOLM-13 cells were transduced with a lentiviral vector encoding the firefly luciferase (ffluc)-green fluorescent protein (GFP) transgene to enable detection by bioluminescence imaging (ffLuc). These cells were kindly provided by Max Jakob Kappenstein and Prof. Dr. Nikolas von Bubnoff, Freiburg, Germany.

### Flow cytometric analysis of CD123 expression on primary bone marrow mononuclear cells

Cell surface expression of CD123 was analyzed on primary AML and healthy donor cells using conjugated mouse anti-human CD123 mAb. Cells were washed with 1× PBS supplemented with 2 mM EDTA, 2% FCS, and 5% sodium azide, resuspended in 100 µL, and stained with 15 µL/test of human anti-CD123 PerCPCy5.5 (BD Biosciences) for 30 min at 4 °C. Cells were also stained with: Live dead Aqua-V500, CD13-PeCy7, CD33-V450, CD34-AF700, CD38-ECD, CD90-BV650, Lin- cocktail (CD3, CD14, CD16, CD19, CD20, CD56)-PB, and CD45RA-PeCy7 (BD Biosciences or Beckman Coulter). Unstained and fluorescence minus one (FMO) controls were used to identify gating boundaries.

### Affinity of the CSL362 anti-CD123 monoclonal antibody

The affinity of the anti-CD123 antibody that was the basis for the ScFv fragment that we used for the anti-CD123 CAR T cells was characterized for its affinity against wild-type (WT) IL-3Rα. The binding affinity was found to be high at: 4.0 ± 1.1 *K*_D_ (nM)^13,56^. The binding affinity of the CSL362 mAb is comparable or higher than the binding affinity of other antibodies/single chains used to generate CD123-directed CAR T cells published by others^10^.

### CAR construction and generation of CAR Lentivirus

A codon-optimized single-chain fragment variant (ScFv) comprising the V_H_ and V_L_ segments of the humanized, and affinity-matured antibody of 7G3, CSL362, was synthesized and developed^13^. The ScFv was fused to a CAR backbone comprising a short IgG4-Fc hinge spacer, a CD28 transmembrane, CD28 followed by an OX40 co-stimulatory moiety, and the CD3ζ signaling domain. The anti-CD123 CAR lentivirus was produced in HEK-293T cells. Briefly, 293T cells were plated one day prior to transfection and cultured in hi-glucose Dulbecco’s minimum essential medium (DMEM) supplemented with 10% FCS and 1% penicillin/streptomycin to achieve 60-80% confluence on the day of transfection. On the day of transfection, 5.5 μg each of the anti-CD123 lentiviral DNA, pMDL-pRRE, pRSV-REV, and PMD2.G packaging plasmid DNA were added to serum-free media (Opti-MEM; Sigma Aldrich, Germany) and Lipofectamine 3000. The mixture was added to the HEK-293T cells following 30 min incubation at room temperature (RT). Cell culture media was replaced after 16 h and cultured for a further 48 h. The viral supernatant was harvested, centrifuged, and filtered through a 0.45 µm membrane filter. The viral supernatant was concentrated by ultracentrifugation, resuspended in 1/150th of the original culture volume with cold sterile 1× PBS, and stored in single-use aliquots at −80 °C until use.

### Preparation of HD-derived CAR-modified T cells

T cells were isolated from healthy donor peripheral blood mononuclear cells (PBMCs) using the human pan T cell isolation kit (Miltenyi Biotec). Purified CD3^+^ T cells were stimulated and cultured with human CD3/CD28 dynabeads (Gibco Biosciences) 2 days prior to lentiviral transduction. Lentiviral gene-transfer was performed at a multiplicity of infection (MOI) range of 5–10. For the NTD T cells, lentiviral-gene transfer was not performed. After 7 days, CD3/CD28 dynabeads were removed from the culture, and T cells successfully transduced with the CD123 CAR were determined by staining with biotinylated recombinant Protein-L (Pierce, ThermoFisher) (1 μg/10^6^ cells), streptavidin-PE (1:20), and 7-AAD viability dye (BD Biosciences, San Diego, CA) (1:100). Successfully transduced CD123 CAR T cells were purified (sort purity >95%) using flow sorting. Purified anti-CD123 CAR T cells were then expanded in culture with recombinant human 100 U/mL interleukin (IL)-2 (Peprotech, Rocky Hill, NJ) for 10 days.

### In vitro T cell subset phenotypic analysis

Anti-CD123 CAR T cells were harvested prior to and post lentiviral transduction and phenotypically analyzed. Cells were washed twice with 1× PBS supplemented with 2 mM EDTA, 2% FCS, and 5% sodium azide, resuspended in 100 µL, and stained with live dead aqua viability dye-V500 (1:500), CD3-PerCPCy5.5, CD8-APC, CD27-APC-ef780, CD45RO-PeCy7 (BD Biosciences) (1:100) for 30 min on ice, in the dark. Cells were washed twice and resuspended in a final volume of 200 µL prior to flow cytometric analysis. Unstained and fluorescence minus one (FMO) controls were used to identify gating boundaries.

### OCI-AML3 AML xenograft model with AZA treatment

For this model, *Rag2*^*–/–*^*Il2rγ*^*–/–*^ recipients were transplanted with 0.2 × 10^6^ OCI-AML3 cells intravenously following sub-lethal irradiation with 3.5 Gy 1 day prior. Mice were subsequently left for 7 days for leukemia to develop. Successful engraftment was confirmed on day 7 using peripheral blood bleeding. Successfully engrafted mice were defined as having >1% hCD45 in the blood. On day 7, mice were randomized and split into six groups: (1) PBS, (2) NTD T cells, (3) anti-CD123 CAR T cells only, (4) AZA only, (5) AZA with NTD T cells, and (6) AZA with anti-CD123 CAR T cells. As the leukemia progression is highly aggressive in this model, a cohort of mice from each group was sacrificed 21 days following T cell injection (instead of 28 for the MOLM-13 xenograft model) for flow cytometry analysis. The remaining cohort of mice from each group was left until day 60 for survival analysis.

### Humanized hematopoietic progenitor cell (HSPC) xenograft model

NSG (NOD-*scid IL2rγ*^null^) mice and humanized cytokine knock-in mice (*CSF1*h/*hIL-3*/*CSF2*h/hh*SIRPA*tg*TPO*h/h*Rag2*^−/−^*IL2rγ*^−/−^), also known as MISTRG-SKI, were used for the experiment. Humanized cytokine knock-in mice with human genes encoding cytokines important for myelopoiesis (macrophage colony-stimulating factor, interleukin-3, granulocyte-macrophage colony-stimulating factor, and thrombopoietin) were generated^57^. Mice were bred and maintained at the University Hospital Zürich animal facility according to the Swiss Federal Veterinary Office guidelines and the Cantonal Veterinary Office Zürich (194/2018). Eight-week-old NSG mice were sub-lethally irradiated with 1.5 Gy and injected intravenously with 0.2 × 10^6^ CD34^+^ isolated human CB hematopoietic progenitor cells^30^. 5 × 10^6^ anti-CD123 CAR T cells or NTD T cells were intravenously transferred 4 weeks following transplantation and confirmed hCD45^+^ myeloid engraftment. For the humanized knock-in mice, newborns were sub-lethally irradiated with 1.5 Gy and injected intra-hepatically with 0.2 × 10^6^ CD34^+^ isolated human CB hematopoietic progenitor cells. 5 × 10^6^ anti-CD123 CAR T cells or NTD T cells were intravenously transferred 6–7 weeks following transplantation and confirmed hCD45^+^ myeloid engraftment. Mice from both strains were killed 16 days following T cell transfer due to high engraftment-associated anemia. This engrafted associated anemia has been observed in the MISTRG-SKI mice^57^. In NSG mice, the engraftment-associated anemia could have been a side effect following T cell infusion. BM, Spleen, and PB were FACS-analyzed for changes in multi-lineage engraftment.

### Flow cytometry

Anti-human antibodies purchased from BioLegend, eBioscience, BD Biosciences, or Becton Dickinson were used for flow cytometry and are listed in the Supplementary Information. For cell surface staining, cells were isolated and washed twice with ice-cold 1× PBS prior to staining at 1:100 dilution with the relevant conjugated antibodies for 30 min on ice, in the dark. For cells isolated from the BM, spleen, or PB of mice, Fc receptor blockade (1:25) (Miltenyi Biotec, Germany) was performed for 15–20 min on ice prior to staining. Cells were washed twice with ice-cold 1× PBS after staining and resuspended in 250 µL 1× PBS supplemented with 2% FCS prior to analysis. For intracellular cytokine staining, cells were treated with 1 µL/mL of Brefeldin A (GolgiPlug) (BD Biosciences, Germany) in cRPMI-1640 medium for 4 h prior to staining using the BD Cytofix/Cytoperm kit (BD Biosciences, Germany) according to manufacturer’s instructions. In all analyses, the population of interest was gated based on forward vs. side scatter followed by singlet gating and dead cell exclusion (7-AAD^−^ or live dead aqua (LDA^−^)). Instrument setup including fluorescence amplification (voltages) and identification of gating boundaries was optimized using unstained and FMO controls. Compensation was optimized using compensation beads (BD Biosciences). Flow analyses were performed on the FACSCanto II or LSRFortessa (BD Biosciences) and the data were analyzed using the FlowJo software v10.4 or v10.6 (Treestar, Ashland, OR).

### T cell-mediated cytotoxicity assay

CellTrace Violet (Invitrogen, ThermoFisher Scientific, Germany) labeled KG1a, MOLM-13, HL-60, ML-2, SUPB15 cells, or primary AML cells (target cells) were used for the T cell-mediated cytotoxicity assay. In brief, anti-CD123 CAR T cells (effector cells) were incubated with target cells at the indicated ratios for 16 h in RPMI-1640 medium (supplemented with 20% FCS, 2 mM glutamine, 100 U/mL penicillin/streptomycin). Percentage-specific lysis of the target cells was determined using flow cytometry. Cells were harvested following the incubation period, stained with 7-AAD (BD Biosciences, Germany) and 10 μL CountBright^TM^ absolute counting beads (Invitrogen, ThermoFisher Scientific, Germany) were added to each sample in a fixed volume just prior to flow acquisition. A uniform number of bead events were acquired for each sample on the flow cytometer. Residual live target cells were CellTrace^TM^ violet^+^ 7-AAD-. Unstained and FMO controls were used to identify gating boundaries.

### AZA treatment of MOLM-13 AML cells and primary cells

MOLM-13, HL-60, ML-2, OCI-AML3, primary AML, or HD cells were maintained in cRPMI-1640 medium and 1 µM AZA (Sigma Aldrich, Germany). For primary AML or HD cells, the culture medium was additionally supplemented with 5 ng/mL recombinant human stem cell factor (hSCF) (Peprotech, Rocky Hill, NJ). The media was replaced every 2–3 days to maintain the cells.

### Colony formation assay

CD34^+^ and CD34^−^ cells were isolated from bone marrow mononuclear cells (BMMNCs) of HD and primary AML samples using the CD34 Ultrapure Microbead kit (Miltenyi Biotec). In the experiments where AZA was used, HD cells were first cultured with 1 μM AZA in RPMI-1640 media supplemented with 5 ng/mL hSCF for 24 h. 5 × 10^3^ CD34^+^ or CD34^−^ cells were then incubated with media alone, or with NTD T cells or anti-CD123 CAR T cells at an effector:target (E:T) ratio of 10:1 in cRPMI-1640 medium for 6 h. Following incubation, the cell suspension was added to a semi-solid methylcellulose-based medium (Methocult opti H3404, Stem Cell Technologies, Vancouver, BC, Canada) and plated into 3 cm tissue culture dishes. Colonies were enumerated using established criteria according to the manufacturer’s instructions and scored using an inverted microscope (Zeiss SteREO Discovery; 4×) after 14 days.

### In vivo T cell subset phenotypic analysis

Bone marrow from the hind legs, and peripheral blood mononuclear cells following cardiac puncture were isolated from MOLM-13 xenograft mice treated with PBS, NTD T cells, CD123 CAR T cells only, AZA only, or dual treated. Cells were washed thrice with 1× PBS, trypan blue counted, and equally distributed. Cells were blocked with human FcR (Miltenyi Biotec) for 20 min. Cells were subsequently stained for residual leukemia cells, residual CAR T cells and its phenotypic subsets, and exhaustion expression on the residual CAR T cells. Cells were stained on ice, in the dark for 45 min. Unstained and FMO controls were used to identify gating boundaries.

### Quantification of absolute cell numbers in bone marrow samples

Bone marrow from hind legs and hip bones were flushed in a fixed volume of PBS (20 mL). Cells from the BM of each mouse were counted based on the flushing volume and equally distributed in the FACS tubes for staining. A fixed number of bead events were acquired during FACS analysis along with a fixed volume for acquisition (150 μL). Absolute cell counts were calculated according to the formula provided by the CountBright^TM^ Absolute counting beads manufacturer’s protocol (Invitrogen, Germany).

### T cell phenotypic exhaustion profiling

Anti-CD123 CAR T cells were harvested at serial time points during the expansion period and analyzed for surface expression of TIM-3, CTLA-4, LAG-3, and PD-1. Briefly, CAR T cells were harvested, CD3/CD28 dynabeads removed, and stained for CTLA-4-BV786, CD8-BV711, TIM-3-BV650, CD3-PerCPCy5.5, PD-1-PE, LAG-3-AlexaFluor 647 (BD Biosciences), and live dead aqua fixable viability stain (Life Technologies) prior to flow cytometric analysis. Unstained and FMO controls were used to identify gating boundaries.

### In vitro CAR T cell functional analysis

#### T cell degranulation

Anti-CD123 CAR T cells and KG1a were incubated at ratios 1:1, 2:1, 5:1, and 10:1 for 4 h at 37^o^C with FITC-CD107a (clone H4A3, BD Biosciences) and the protein transport inhibitor monensin (BD GlogiStop)^58^. SUPB15 (CD123-) cells were used as a control. Following incubation, cells were stained with CD3-PerCPCy5.5, CD8-APC (BD Biosciences), and live dead aqua fixable dead cell stain (Life Technologies) to determine the percentage of CD107^+^ CD4 and CD8 CAR T cells by flow cytometry. 1 µg/mL Staphylococcus aureus, Enterotoxin type B (SEB) (Merck Millipore) was used as a positive control of degranulation. Negative controls received RPMI-1640 culture medium (supplemented with 10% FCS) or RPMI-1640 media with anti-CD123 CAR T cells only.

#### CellTrace violet proliferation assay

Anti-CD123 CAR T cells were labeled with CellTrace Violet (Life Technologies) according to the manufacturer’s instructions. KG1a or SUPB15 cells were irradiated at a dose of 100 Gy. T cells were incubated at a ratio of 1:1 with irradiated target cells for 120 h. Cells were then harvested, stained for CD3-PerCPCy5.5, CD8-BV711, and Live dead far-red fixable viability stain (Invitrogen) prior to flow cytometric analysis.

#### Secretory cytokine measurement

Anti-CD123 CAR T cells (effectors) and KG1a cells (targets) were co-cultured at a E:T ratio of 10:1 at 37 °C for 24 h. For controls, SUPB15 cells were used as CD123^−^ targets. The supernatant was harvested and stored in single-use aliquots at −80 °C until use. The supernatant was analyzed using the human 30-plex milliplex kit according to the manufacturer’s protocol (Merck Millipore).

### Epithelial tissue damage histological scoring

Mice were transplanted as described in the MOLM-13 AML^FLT3 ITD^ xenograft model. Samples from the small intestine, colon, and liver were collected 28 days following T cell infusion. Samples were prepared, cut into 3 μm sections, and stained with Hematoxylin & Eosin (H/E). The presence of epithelial tissue damage was determined and scored by an experienced pathologist (K.A.) blinded to the treatment groups. Tissue damage severity was determined according to an established histopathology scoring system^59^.

### Pharmaceutical drugs

5′-Azacitidine (Sigma Aldrich, Munich, Germany) was reconstituted in sterile DMSO (Sigma Aldrich, Munich, Germany) prior to dilution in sterile dH_2_O and used in in vitro and in vivo experiments.

### Optimization of azacitidine concentration In Vitro

In human plasma, *C*_max_ values of AZA are 3–11 µM in order to observe substantial immunomodulatory and, especially, anti-leukemic effects^60,61^. In our in vitro studies, preliminary dose escalation studies for the cell lines demonstrated no further enhanced immunomodulatory effects beyond 1 µM. An immediate (12 h following addition of AZA to cells) and a significant decrease in cell viability was observed in all AML cell lines with concentrations higher than 1 µM. For the purposes of these studies, 1 µM AZA was therefore used in all in vitro experiments since the focus was aimed at the immunomodulatory effects of AZA and not anti-leukemic activity.

### Optimization of azacitidine administration In Vivo

The dose and schedule of AZA administration were chosen based on published data. The minimum dose with therapeutic benefit/immunomodulatory effects was 2 mg/kg^62^. The maximum tolerated dose reported was 5 mg/kg in a pre-clinical model of AML using immunocompromised mice^63^. Since the aim of the study was not to use AZA for anti-leukemic purposes, the concentration of AZA used was 2.5 mg/kg with the same schedule of administration as the study conducted with AML xenografts^63^. In preliminary experiments, no significant anti-leukemic effects were observed. All in vivo experiments involving AZA, herein, followed this concentration and dosing schedule.

### Whole-genome bisulfite sequencing (WGBS) and data analysis

Whole-cell lysates of MOLM-13, HL-60, and OCI-AML3 cells treated with or without AZA were used for DNA or RNA isolation. For library preparation, the Ovation Ultralow Methyl-Seq DR Multiplex System (NuGEN/TECAN) was used. The EpiTect Fast Bisulfite Kit (Qiagen) was used for bisulfite conversion. Library quantification and fragment size estimation of the finished libraries were performed with Qubit dsDNA HS Assay Kit (ThermoFisher) and Agilent High Sensitivity DNA Kit (Agilent), respectively. Libraries were pooled and sequenced on a Nextseq 2000 P3 100PE (Illumina®). De-multiplexing was performed by the EMBL Gene Core. For bioinformatic analysis, we used tools integrated in the Galaxy server platform^64^. Sequencing data were mapped to human genome assembly hg38 using bwameth^65^. Reads were trimmed with Trim Galore (https://github.com/FelixKrueger/TrimGalore) prior mapping. PCR duplicate removal was performed with RmDup (SAMtools)^66^. Extraction of methylation data was done with MethylDackel (https://github.com/dpryan79/MethylDackel). MethylseekR^67^ software was used for the segmentation of the DNA methylation data. LMRs and UMRs were called with default settings and a false discovery rate of <5 %. For further analysis, UMRs overlapping the TSS (±1 kb) and LMRs outside the TSS (±1 kb) were used. Heatmaps and profile plots were generated with DeepTools^68^.

### Statistical analyses

Statistical analyses and significance were determined using GraphPad Prism v7.01 or v8.2.1 software (GraphPad Software Inc. LA Jolla, CA). All data were tested for normality applying the Kolmogorov-Smirnov and Shapiro-Wilk test. Based on the results of the normality tests, paired Student’s *t-*test, unpaired or paired parametric (two-sided) Student’s *t*-test, unpaired non-parametric (two-sided) Student’s *t*-test (Mann–Whitney test), ordinary two-sided one-way ANOVA (Tukey’s test) or one-way ANOVA (Kruskal–Wallis test with Dunn’s multiple comparisons) or two-sided Log-rank (Mantel–Cox) was used, where appropriate, for comparing differences between groups. A log-rank test was used to calculate significance between survival curves. All graphed data, except for survival curves, are presented as median or mean ± standard error of the mean (SEM). In all cases, a *p*-value of ≤0.05 was considered significant. Exact *p*-values are given whenever suitable.

### Reporting summary

Further information on research design is available in the Nature Research Reporting Summary linked to this article.

## Supplementary information

Supplementary information

Reporting summary

## Data Availability

The RNA-sequencing data in this study have been deposited in the GEO repository under accession code GSE184891. The whole-genome bisulfite sequencing data in this study have been deposited in the BioProject repository under accession code PRJNA766490. All other processed data generated in this study as depicted in the main and Supplementary Figures are provided as a source data file. Source data are provided with this paper.

## Source data

Source data
