# Supplementary material for: Demethylating therapy increases anti-CD123 CAR T cell cytotoxicity against acute myeloid leukemia
Source: Nat Commun. 2021 Nov 8;12:6436. doi: 10.1038/s41467-021-26683-0 (PMC8575966; doi:10.1038/s41467-021-26683-0)
Supplement: Supplementary file 1 — Supplementary information [file 41467_2021_26683_MOESM1_ESM.pdf]

Supplementary Information

Supplementary Figure 1: Phenotypic analysis of CD123 expression on primary healthy donor bone marrow cells

Supplementary Figure 2: CD123 is overexpressed on primary AML while low to absent on healthy donor cells

Supplementary Figure 3: Third-generation anti-CD123 CAR T cell construct and the phenotypic evaluation of the CAR T cells prior to functional analysis

Supplementary Figure 4: In vitro effector functions of anti-CD123 CAR T cells against AML cell lines

Supplementary Figure 5: Third-generation anti-CD123 CAR T cells recognize and eliminate $CD123^+$ AML cells in vitro

Supplementary Figure 6: Treatment of AML cells with azacitidine induces global DNA methylation and increased CD123 expression in vitro and in vivo

Supplementary Figure 7**:** AZA treatment supports the increased anti-leukemic effect of anti-CD123 CAR T cells in OCI-AML3 xenograft mice

Supplementary Figure 8: Treatment of AML with azacitidine and CD123 CAR T cells does not cause epithelial tissue damage

Supplementary Figure 9: The effect of anti-CD123 CAR T cells on normal hematopoietic progenitor cell development in human cord blood engrafted MISTRG-SKI mice

Supplementary Figure 10: Analysis of residual T cells and their expression for exhaustion markers in the bone marrow of MOLM-13 AML xenograft mice

Supplementary Figure 11: Analysis of residual T cells and their expression for exhaustion markers in the peripheral blood of MOLM-13 AML xenograft mice

Supplementary Figure 12: Analysis of residual $CD4^+$ and $CD8^+$ T cell subsets in the bone marrow and peripheral blood of MOLM-13 AML xenograft mice

Supplementary Figure 13: Analysis of residual T cells and their expression for exhaustion markers in the bone marrow of OCI-AML3 AML xenograft mice

Supplementary Figure 14: Analysis of residual T cells and their expression for exhaustion markers in the peripheral blood of OCI-AML3 AML xenograft mice

Supplementary Figure 15: Analysis of residual $CD4^+$ and $CD8^+$ T cell subsets in the bone marrow and peripheral blood of OCI-AML3 AML xenograft mice

Supplementary Figure 16: Analysis of CTLA-4^{negative} and TNFα expression on residual CD8^{+} T cells in the bone marrow and peripheral blood of MOLM-13 AML xenograft mice treated with CTLA-4^{negative} or CTLA-4^{positive} anti-CD123 CAR T cells

Supplementary Figure 17: CTLA-4^{+} and CTLA-4$_{-}$ CD123 CAR T cells demonstrate higher lytic capacity against AZA treated AML cells compared to untreated AML cells

Supplementary Figure 18: Influence of CTLA-4^{+} anti-CD123 CAR T cells on CTLA-4$_{-}$ anti-CD123 CAR T cell function

Supplementary Figure 19: Assessment of the phosphorylation of intracellular Lck and Zap70 in CD8^{+} anti-CD123 CAR T cells in the presence of MOLM-13 AML cells

Supplementary Table 1: AML Patient Characteristics (SAHMRI, Adelaide, Australia)
Supplementary Table 2: AML Patient Characteristics (University Medical Clinic, Freiburg, Germany)
Supplementary Table 3: Antibodies for flow cytometry
Supplementary Table 4: Immune response genes and associated transcripts depicted in Supplementary Figure 6

Supplementary Figure 1

a

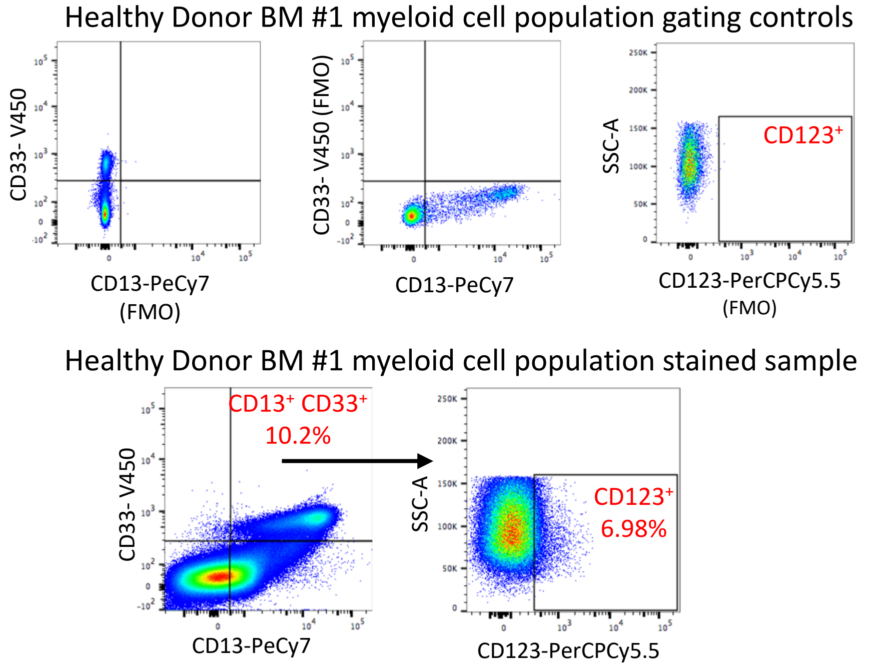

b

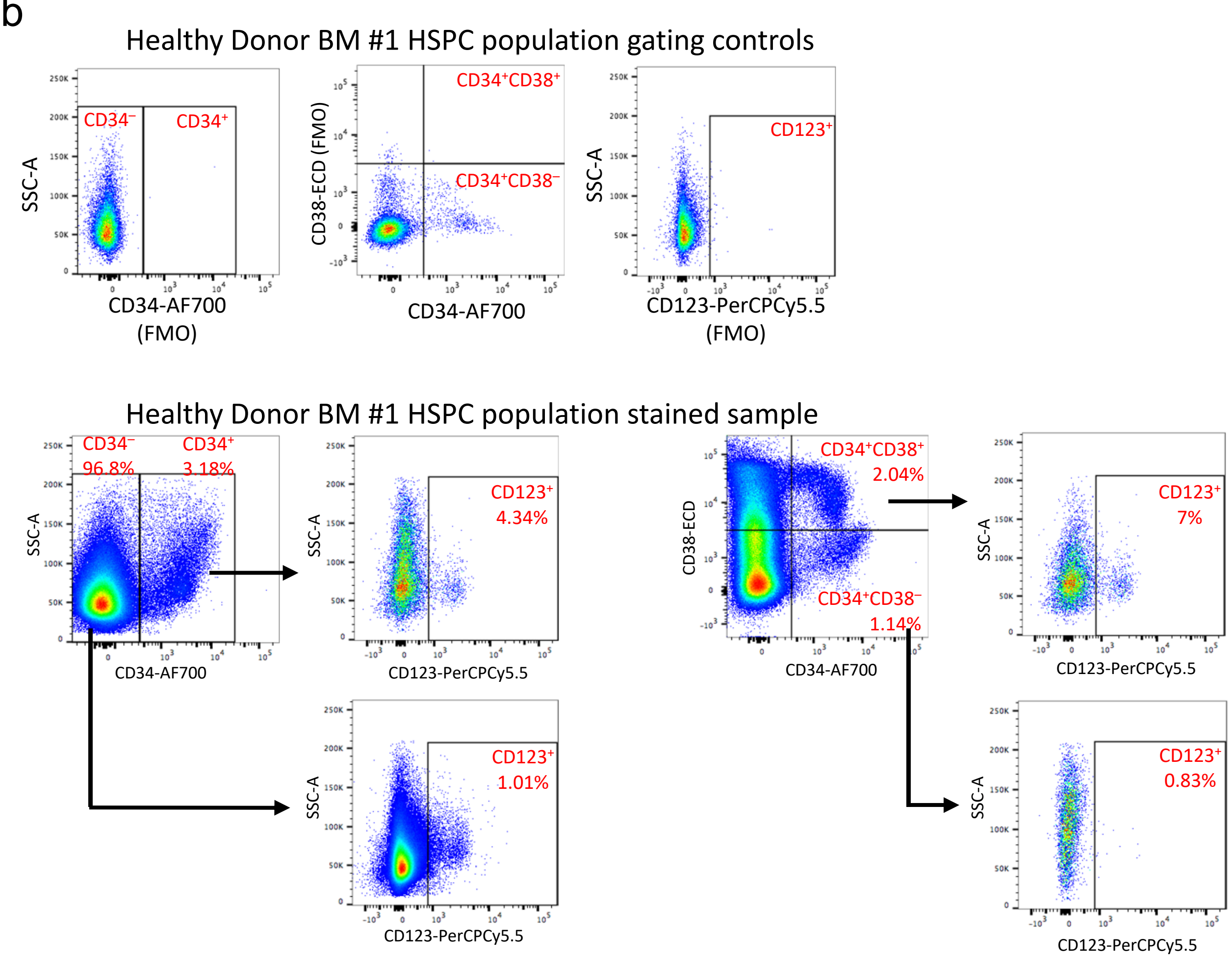

Supplementary Figure 1. Phenotypic analysis of CD123 expression on primary healthy donor bone marrow cells. a Representative flow cytometry gating strategy, including fluorescence minus one (FMO) controls, used to evaluate CD123 expression on bulk healthy donor (HD) myeloid cells. **b** Representative flow cytometry gating strategy, including fluorescence minus one (FMO) controls, used to evaluate CD123 expression on HD stem and progenitor cell populations. In all cases, gates were first set on bulk AML or healthy donor (HD) cells (SSC v FSC), following this; viable single cells were defined based on FSC-A v FSC-H and exclusion of dead cells by live dead aqua viability stain (LDA^{-}). Specific cell populations were then gated from the live single cells.

Supplementary Figure 2

a

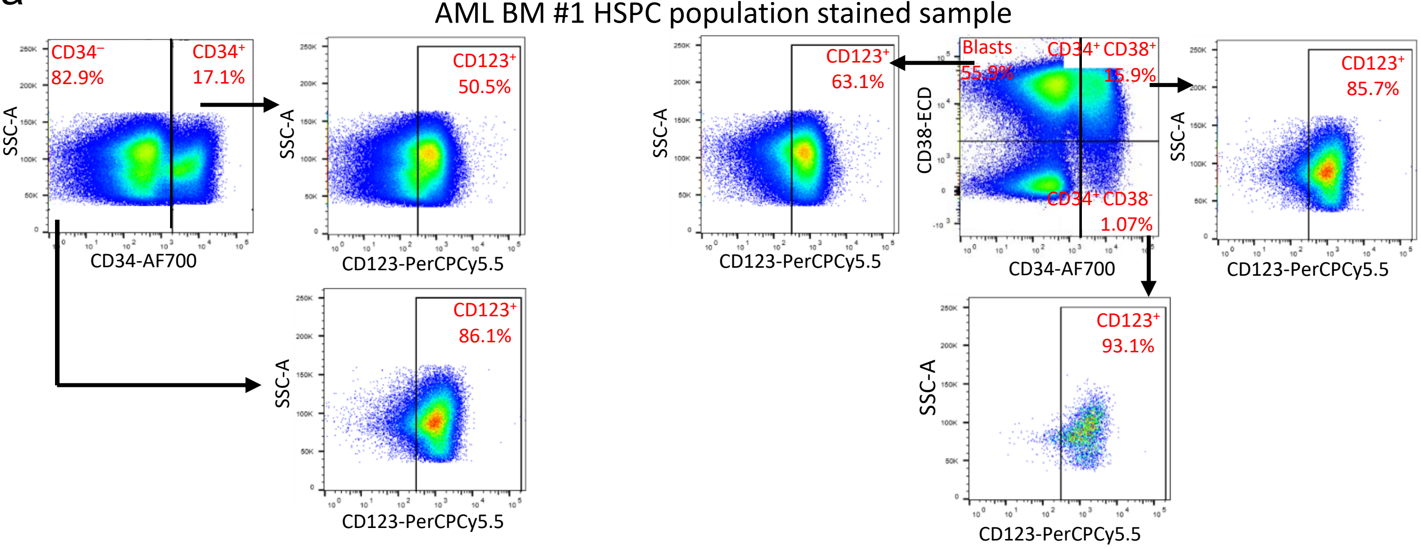

b

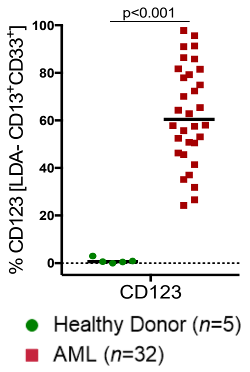

c

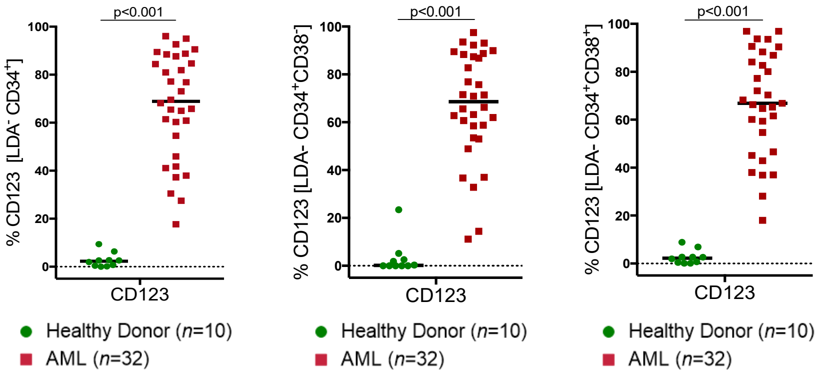

d

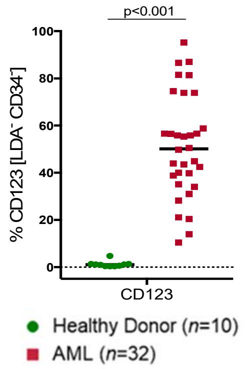

Supplementary Figure 2. CD123 is overexpressed on primary AML while low to absent on healthy donor cells. a Representative flow cytometry gating strategy used to evaluate CD123 expression on acute myeloid leukemia (AML) stem and progenitor cells. Gates were first set on bulk AML or healthy donor (HD) cells (SSC v FSC), following this; viable single cells were defined based on FSC-A v FSC-H and exclusion of dead cells by live dead aqua viability stain (LDA^-). Specific cell populations were then gated from the live single cells. CD123 expression was evaluated on **b** $CD13^+CD33^+$ bulk myeloid cells in HD BMMC (*n*=5) and relapsed/refractory AML patient BMMC samples (*n*=32) at diagnosis. CD123 expression on **c** $CD34^+$ bulk primitive cells (left panel), $CD34^+CD38^-$ progenitor cells (middle panel), $CD34^+CD38^+$ HSC/MPP populations (right panel), and **d** $CD34^-$ bulk mature/differentiated cells were also evaluated in HD bone marrow mononuclear cells (BMMC) (*n*=10) and relapsed/refractory AML patient BMMC samples (*n*=32) at diagnosis. Data are represented as individual values with bars representing the median value of each group. *p*-values were calculated using unpaired two-sided student's *t*-test (Mann-Whitney).

Supplementary Figure 3

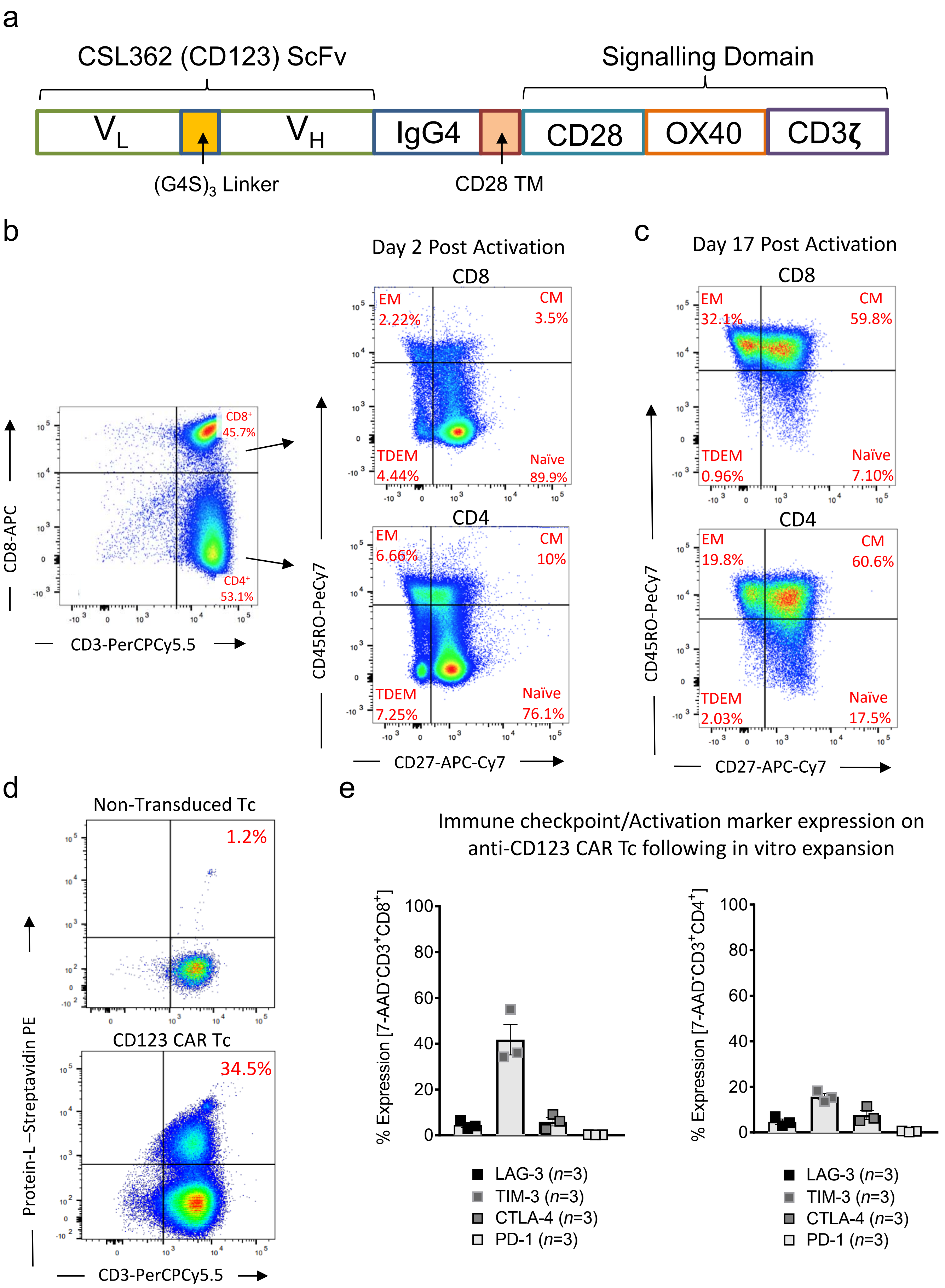

Supplementary Figure 3. Third-generation anti-CD123 CAR T cell construct and the phenotypic evaluation of the CAR T cells prior to functional analysis. a Schematic of the third-generation CAR construct. The single chain variable fragment (ScFv) was derived from the fully humanized CD123 (CSL362) neutralizing antibody connected with a $(G4S)_3$ linker. The ScFv was fused to an IgG4 hinge spacer, CD28 transmembrane domain, and intracellular signaling module. The intracellular signaling module consists of two co-stimulatory molecules: CD28 and OX40 which are attached to the CD3ζ chain. **b** Representative flow cytometry dot plots demonstrating the phenotype of healthy donor derived T cells prior to activation with CD3/CD28 dynabeads and CD123 CAR lentiviral transduction. The majority of T cells from healthy donors (HD) were $CD4^+$ phenotype (left panel). The majority of $CD8^+$ (right top panel) and $CD4^+$ (right lower panel) T cells possess a naive phenotype prior to activation. However, a small portion of cells are phenotypically central memory (CM), effector memory (EM) or terminally differentiated effector memory (TDEM). **c** Representative flow cytometry dot plots demonstrating the change in phenotype of healthy donor derived T cells following 17 days of activation and expansion, and post CD123 CAR lentivirus transduction. By day 17 the majority of $CD8^+$ (top panel) and $CD4^+$ T cells (bottom panel) possess a CM or EM phenotype. **d** Representative flow cytometry dot plot showing the expression of $CD3^+$ T cells transduced with Protein-L on day 5 post transduction. Cells that are double positive for CD3 and Protein-L successfully incorporated the CD123 CAR. Non-transduced (NTD) T cells are included for comparison. Cells were then enriched for $CD3^+$ Protein-L^+ by flow sorting prior to functional testing. **e** Scatter plots depicting the expression of exhaustion markers: LAG-3, TIM-3, CTLA-4, and PD-1 on $CD8^+$ T cells (*n*=3) (left panel) and $CD4^+$ T cells (*n*=3) (right panel) 17 days following activation and pre-exposure to $CD123^+$ target cells. The data shown are from 3 independent transductions and are presented as mean ± SEM.

Supplementary Figure 4

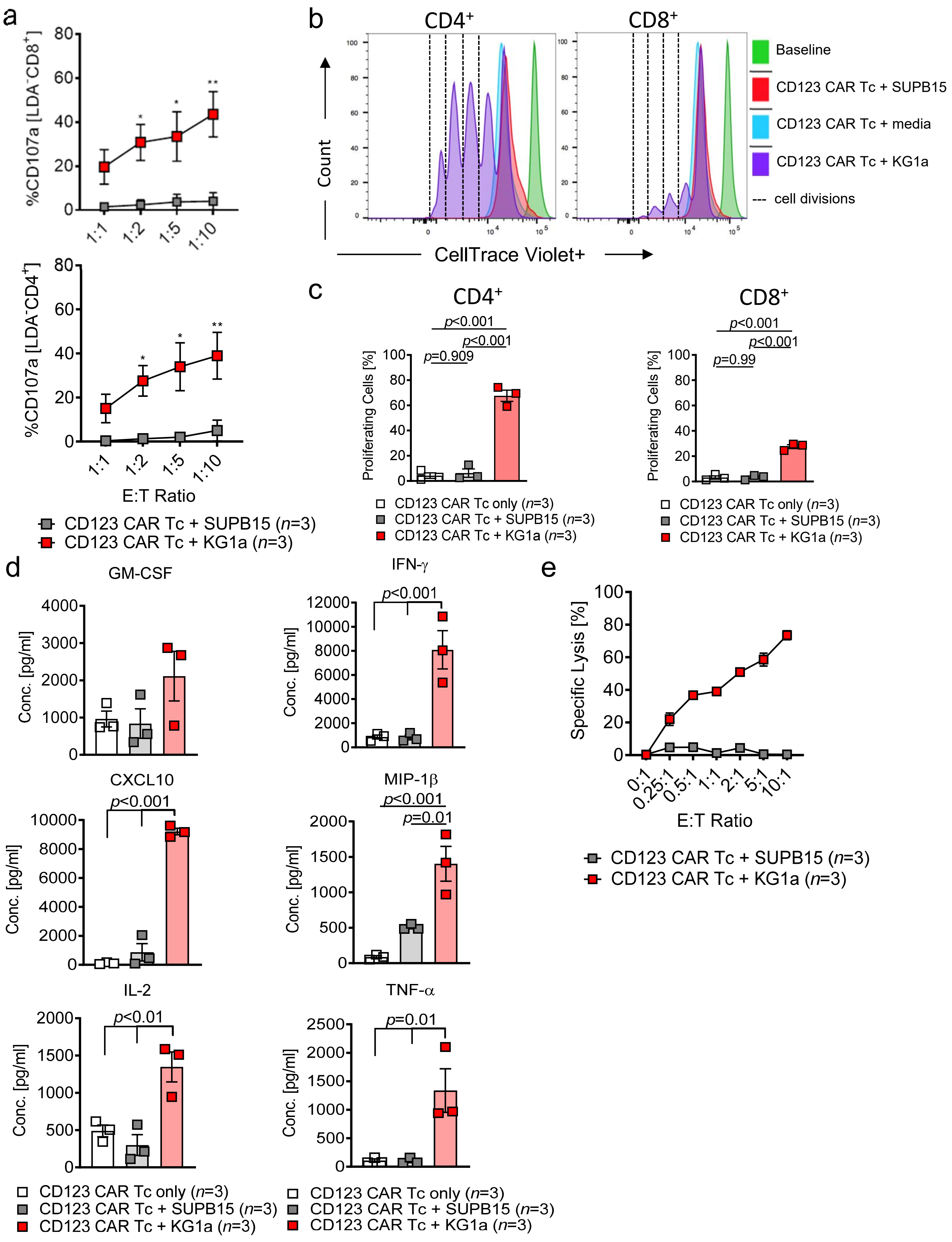

Supplementary Figure 4. In vitro effector functions of anti-CD123 CAR T cells against AML cell lines. a CD123 $CD8^+$ (top panel) and $CD4^+$ (bottom panel) CAR T cells were co-cultured for 4 h with KG1a ($CD123^+$) (*n*=3) or SUPB15 ($CD123^-$) (*n*=3) cells at different E:T ratios and analyzed for surface CD107a expression. **b** Representative histograms depicting the proliferation of $CD8^+$ (left panel) and $CD4^+$ (right panel) anti-CD123 CAR T cells examined by CellTrace^{TM} violet dye dilution following 96 h of co-culture with media (untreated), KG1a or SUPB15 cells at an E:T ratio of 1:1. Each dotted line represents one cell division. **c** Scatter plot graphs denoting the total proliferating $CD8^+$ and $CD4^+$ CAR T cells following the 96 h co-culture with media (*n*=3), KG1a (*n*=3) or SUPB15 cells (*n*=3). **d** Anti-CD123 CAR T cells were co-cultured with media only (untreated) (*n*=3), KG1a (*n*=3) or SUPB15 cells (*n*=3) at an E:T ratio of 10:1 for 24 h. The supernatant was analyzed and quantified for the release of various cytokines. The cytokines/chemokines with significant differences between each treatment group are depicted. **e** Specific cytotoxicity of anti-CD123 CAR T cells against KG1a (*n*=3) or SUPB15 cells (*n*=3) (CellTrace^{TM} violet labelled) by flow cytometric analysis following a 16 h co-incubation. Assay was performed in triplicate with a fixed number of target cells/well for all E:T ratios. Counting beads were used to quantify the absolute number of residual live target cells at the end of the co-culture. Residual live target cells were CellTrace violet$^+$ 7-AAD$^-$. All data are pooled from 3 independent experiments, each plated in technical duplicates and presented as mean ± SEM. *p*-values were calculated using two-sided one-way ANOVA (Kruskal-Wallis test with Dunn's multiple comparison).

Supplementary Figure 5

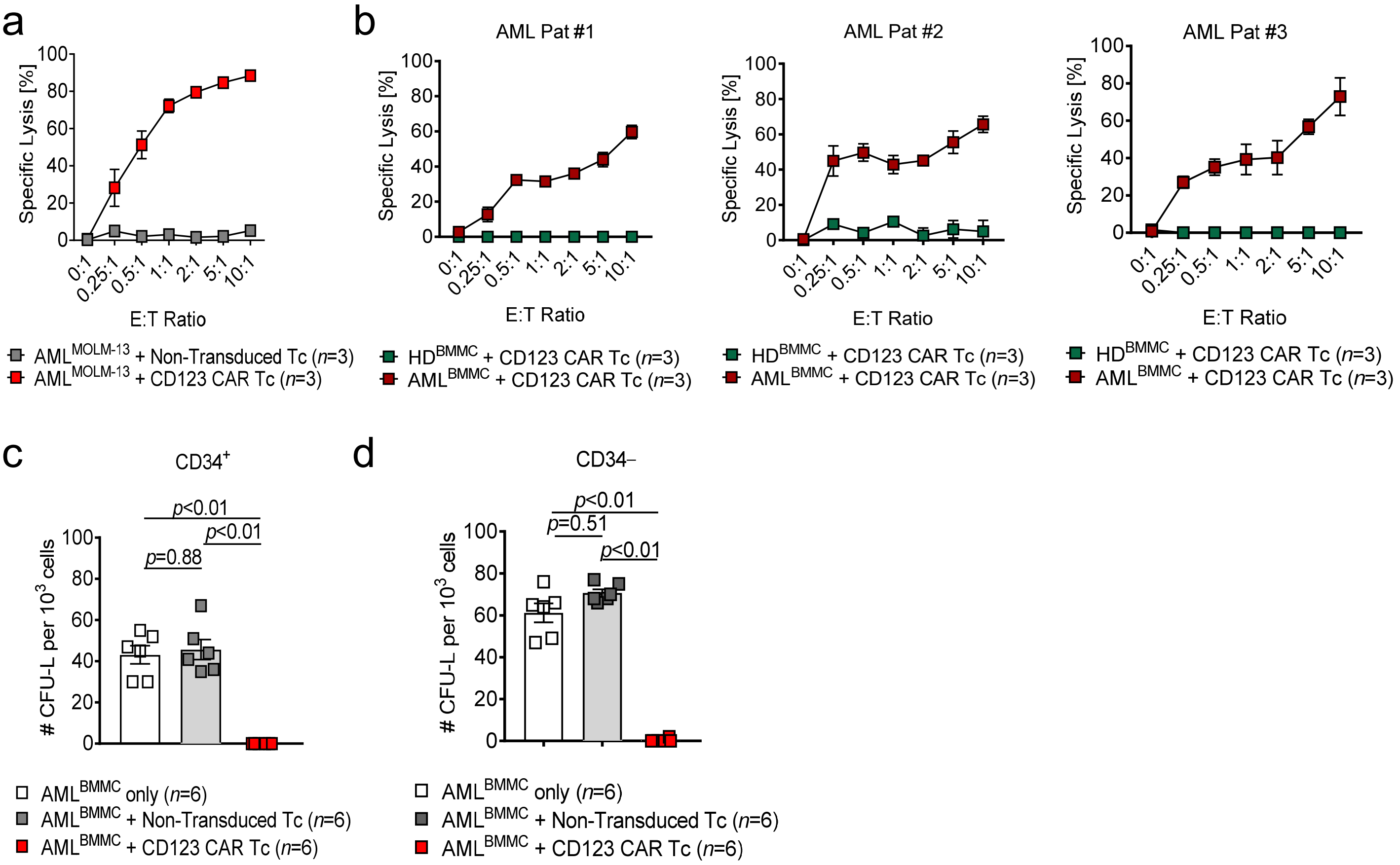

Supplementary Figure 5. Third-generation anti-CD123 CAR T cells recognize and eliminate $CD123^+$ AML cells in vitro. a Specific cytotoxicity of anti-CD123 CAR T cells (*n*=3) or NTD T cells (*n*=3) against $CD123^+$ MOLM-13 cells (CellTrace violet labelled) by flow cytometric analysis following a 16 h co-incubation. **b** Specific cytotoxicity of anti-CD123 CAR T cells against 3 patient primary AML bone marrow cells with varying $CD123^+$ expression or healthy donor (HD) bone marrow (BM) cells (CellTrace violet labelled) by flow cytomeric analysis following a 16 h co-incubation. In both cases, the assays were performed (*n*=3) for each patient sample with a fixed number of target cells/well for all E:T ratios. Counting beads were used to quantify the absolute number of residual live target cells at the end of the co-culture. Residual live target cells were CellTrace $violet^+$ $7\text{-}AAD^-$. **c** $CD34^+$ or **d** $CD34^-$ cells were immunomagnetically selected and co-cultured in media only (untreated), with anti-CD123 CAR T cells or NTD T cells for 6 h at an E:T ratio of 10:1. The cells were subsequently plated in semisolid methylcellulose progenitor media, cultured for 14 days, and scored using an inverted microscope for the presence of leukemia colony-forming units (CFU-L). The experiment was performed and pooled using 3 different primary AML patient samples, each plated in duplicates. Colony numbers are represented per 1000 plated cells. All graphed data are represented as mean values ± SEM. *P*-values **(c, d)** were calculated with two-sided one-way ANOVA (Kruskal-Wallis test with Dunn's multiple comparison).

Supplementary Figure 6

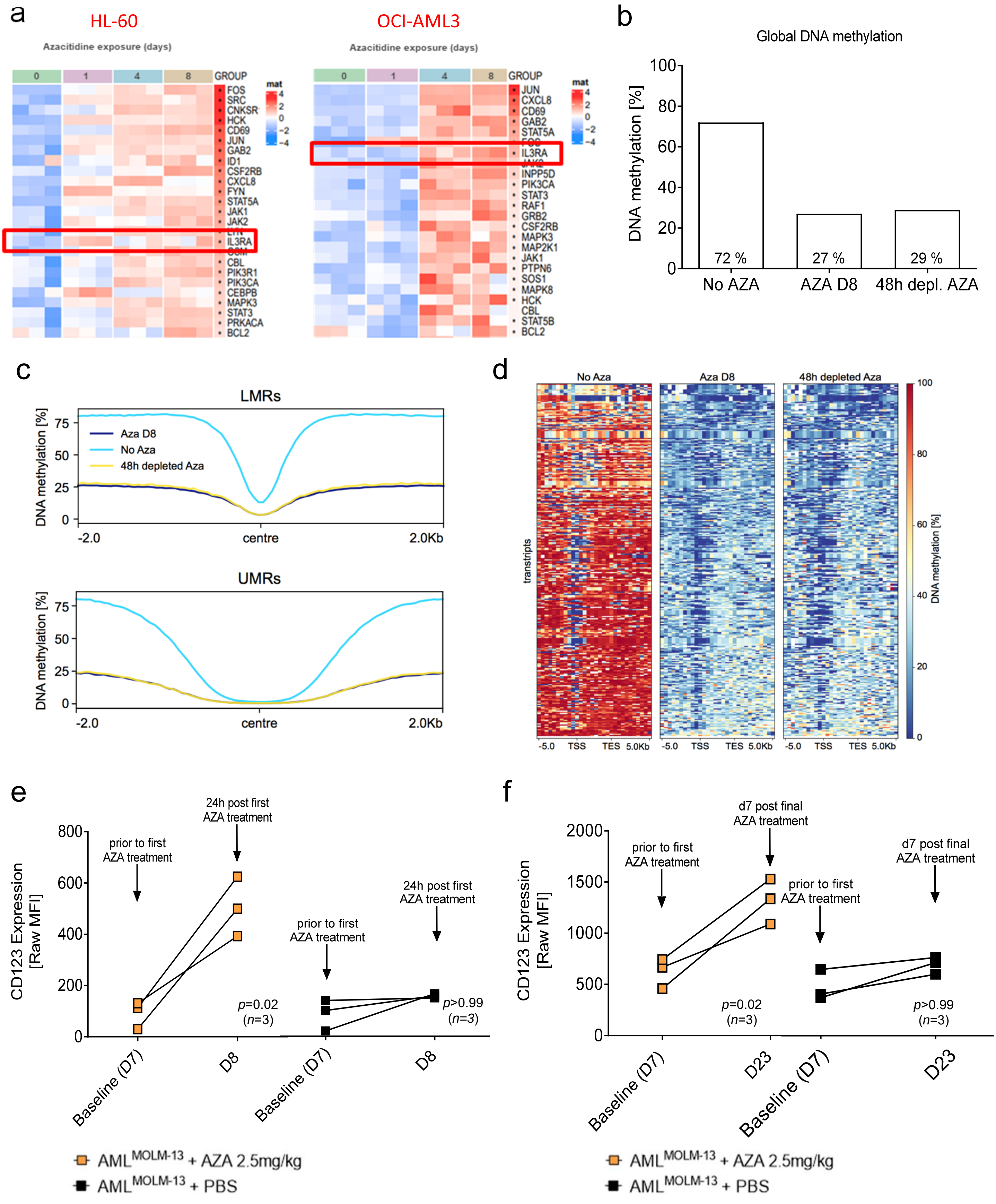

Supplementary Figure 6. Treatment of AML cells with azacitidine induces global DNA methylation and increased CD123 expression in vitro and in vivo. a Heatmap depicting (in particular highlighting IL3RA/CD123 in red) upregulated genes in HL-60 and OCI-AML3 in the absence (day 0) or presence of (1,4, and 8 days) 1μM azacitidine (AZA) treatment in culture. **b** Bar graph depicting the percentage of mean global DNA methylation analysis of MOLM-13 AML cells in cells without AZA treatment, AZA treated (1μM) for 8 days, or cells treated with AZA (1μM) for 8 days then depleted from AZA for 48 h. **c** Profile plot of low methylated and un-methylated regions in MOLM-13 cells without AZA treatment (light blue), AZA treated cells (1μM) for 8 days (dark blue), and cells treated with AZA (1μM) for 8 days then depleted from AZA for 48 h (yellow). **d** DNA methylation of immune response genes and associated transcripts (in ascending order as listed in Supplementary Table 4) in MOLM-13 cells without AZA treatment, AZA treated (1μM) for 8 days, or cells treated with AZA (1μM) for 8 days then depleted from AZA for 48 h. **e** Scatter plot showing the raw mean fluorescence intensity (MFI) expression of CD123 at D7 (prior to first AZA treatment; 2.5mg/kg) (*n*=3) and D8 (24 h post first AZA treatment; 2.5mg/kg) (*n*=3) in the peripheral blood (PB) of $AML^{MOLM\text{-}13}$ bearing mice treated with AZA and $AML^{MOLM\text{-}13}$ bearing mice treated with phosphate buffered saline (PBS) (*n*=3). **f** Scatter plot showing the raw MFI expression of CD123 at D7 (prior to first AZA treatment; 2.5mg/kg) (*n*=3) and D23 (7 days post the final AZA treatment; 2.5mg/kg) (*n*=3) in the PB of $AML^{MOLM\text{-}13}$ bearing mice treated with AZA and $AML^{MOLM\text{-}13}$ bearing mice treated with PBS (*n*=3). *p*-values were calculated with two-sided paired student's *t*-test.

Supplementary Figure 7

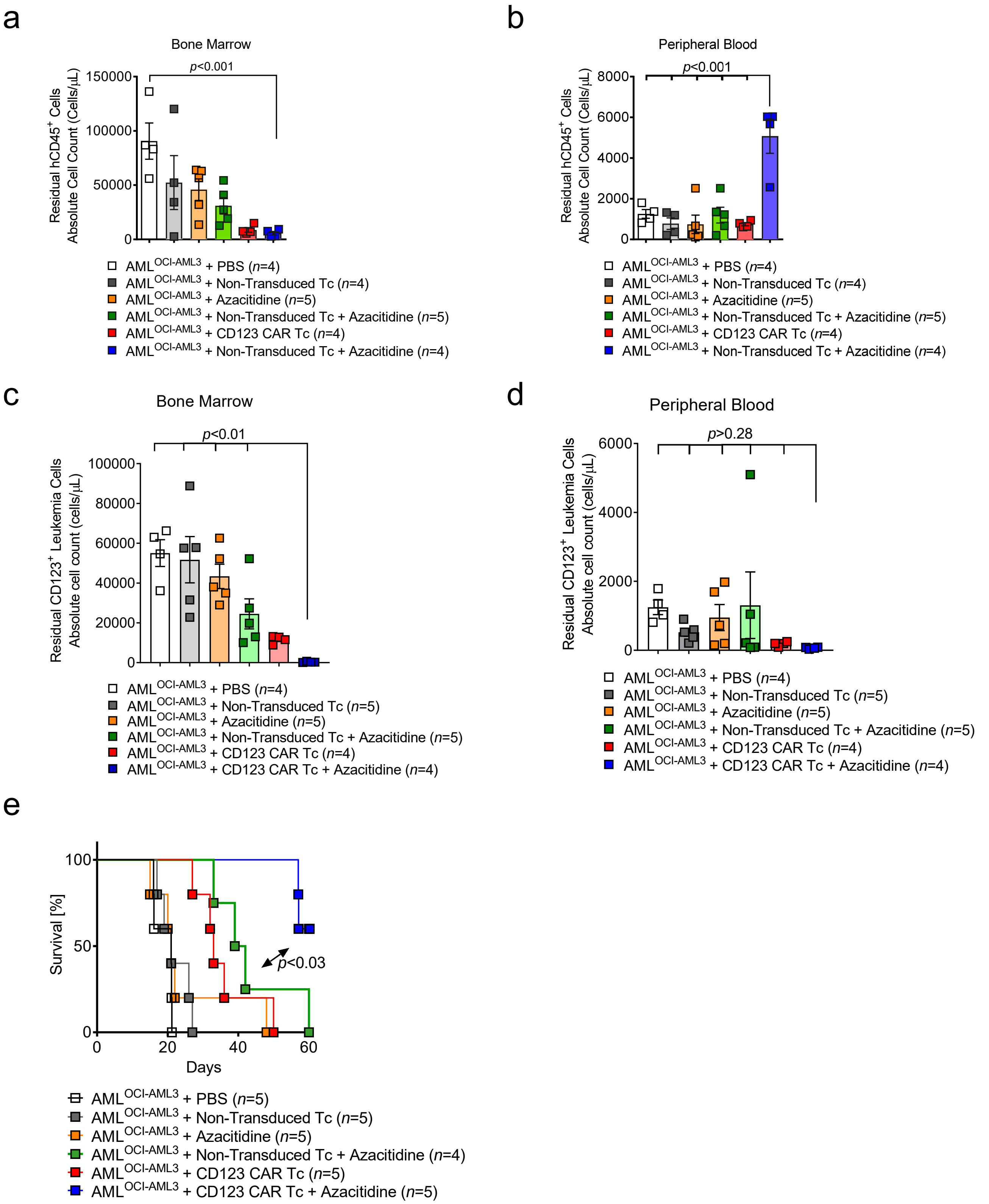

Supplementary Figure 7. Azacitidine treatment supports the increased anti-leukemic effect of anti-CD123 CAR T cells in OCI-AML3 xenograft mice. Flow cytometric analysis depicting absolute counts (cells/μL) of residual **a** bone marrow (BM) hCD45+, **b** peripheral blood (PB) hCD45+, **c** residual $CD123^+$ leukemia cells in the BM, and **d** residual $CD123^+$ leukemia cells in the PB of the various treatment groups (PBS *n*=4; NTD Tc *n*=4 (a, b) *n*=5 (c-e); AZA *n*=5; NTD Tc + AZA *n*=5; CD213 CAR Tc *n*=4; CD123 CAR Tc +AZA *n*=4). **e** Kaplan-Meier analysis of percentage survival for each treatment group (PBS *n*=5; NTD Tc *n*=5; AZA *n*=5; NTD Tc + AZA *n*=4; CD213 CAR Tc *n*=5; CD123 CAR Tc +AZA *n*=5). Attrition of mice was due to paralysis in hind legs, growth of subcutaneous tumors (>2cm) or physical deterioration of the mice. All data are represented as mean ± SEM. *p*-values were calculated using two-sided one-way ANOVA (Kruskal-Wallis test with Dunn's multiple comparison) **a-d**. *p*-values for survival analysis **e** was calculated using two-sided Mantel-Cox test (log-rank). For statistical analysis of survival, all groups were compared to the $AML^{OCI-AML3}$ + CD123 CAR Tc + AZA group.

Supplementary Figure 8

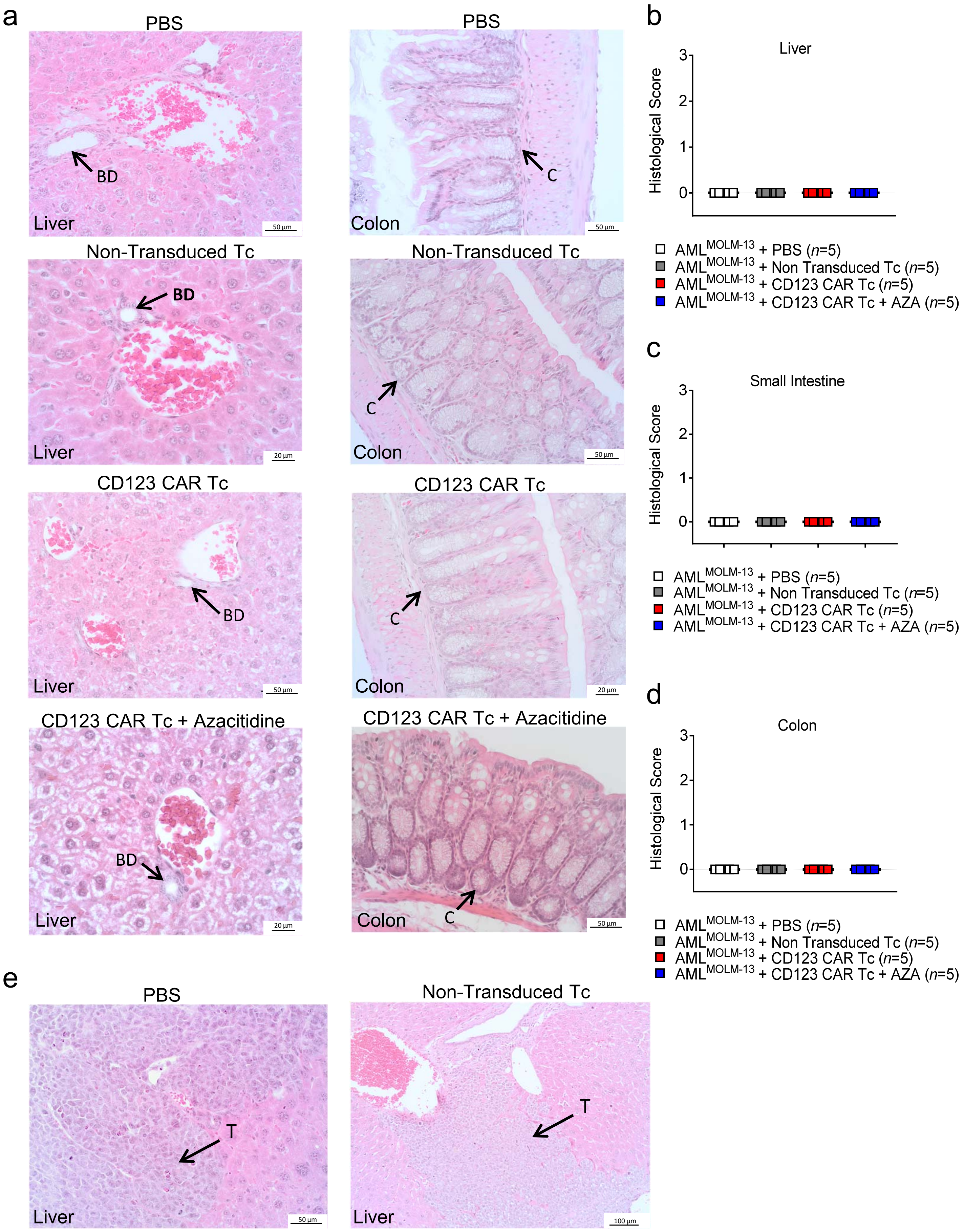

Supplementary Figure 8. Treatment of AML with azacitidine and anti-CD123 CAR T cells do not cause epithelial tissue damage. a Representative hematoxylin and eosin (H&E) staining of liver and colon tissues of MOLM-13 engrafted $Rag2^{-/-}Il2r\gamma^{-/-}$ mice treated with PBS, $5x10^6$ NTD T cells, $5x10^6$ CD123 anti-CAR T cells, or $5x10^6$ anti-CD123 CAR T cells + AZA to detect the presence of epithelial tissue damage. Images were taken at magnifications: 100x, 200x, 400x (represented on the scale bar as 20μm, 50μm, 100μm, respectively). **b-d** Liver, small intestine, and colon tissues of PBS (*n*=5), NTD T cells (*n*=5), anti-CD123 CAR T cells (*n*=5) or anti-CD123 CAR T cells + AZA (*n*=5) were scored for degree of tissue damage and quantified. Data were pooled from 2 independent experiments. **e** Representative H&E staining of liver tissue from MOLM-13 engrafted $Rag2^{-/-}Il2r\gamma^{-/-}$ mice treated with PBS or NTD T cells. Images show tumor cell infiltration in the liver tissues. Images were taken at magnifications 200x and 400x (represented on the scale bar as 50μm and 100μm). Abbreviations: *BD*: Biliary duct- without intraepithelial lymphocytic infiltration and without bile duct destruction. *C*: crypt- no apoptosis within the epithelium of the crypt base and without crypt destruction. *T*: tumor- infiltration of tumor cells with undifferentiated phenotype.

Supplementary Figure 9

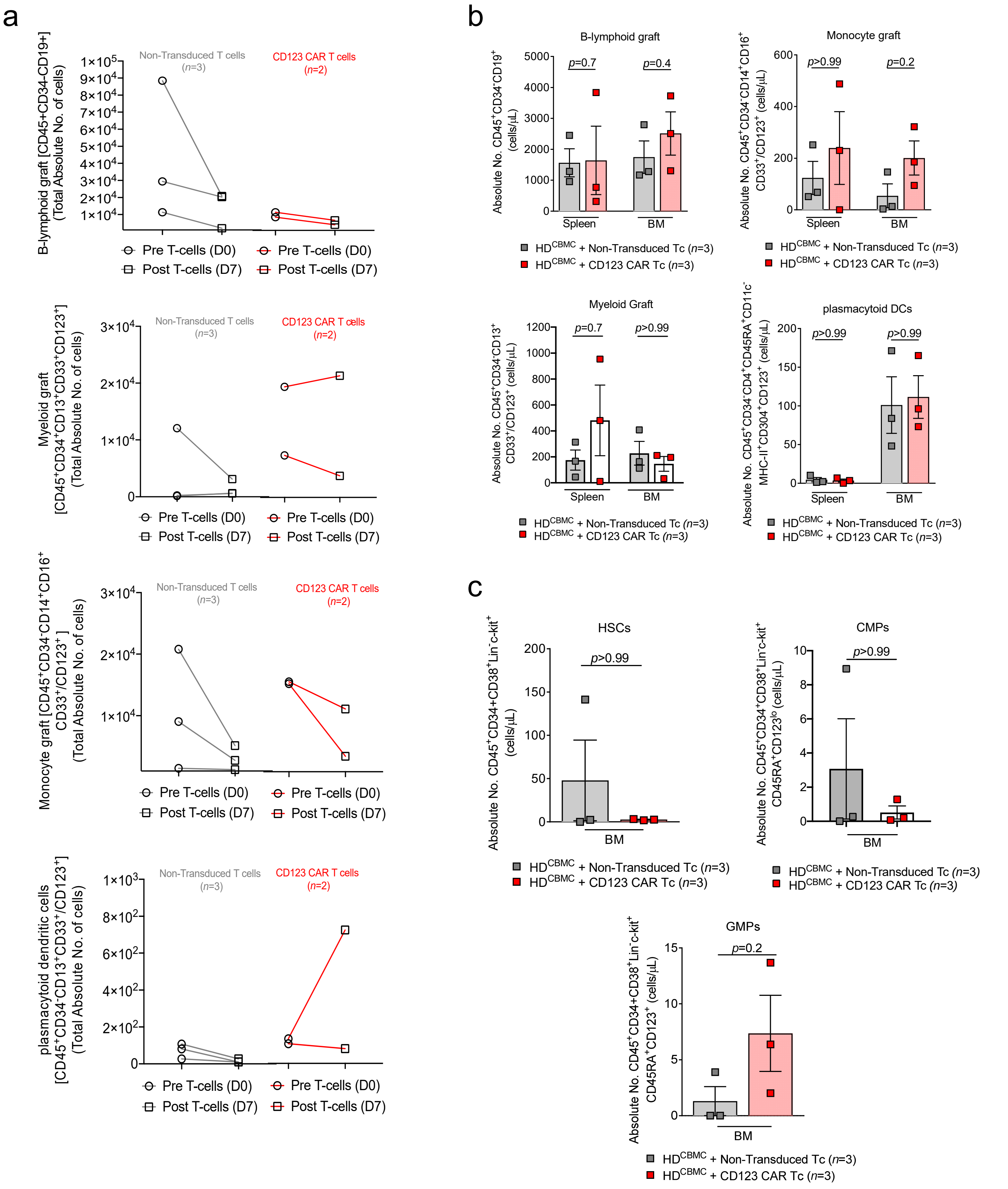

Supplementary Figure 9. The effect of anti-CD123 CAR T cells on normal hematopoietic progenitor cell development in human cord blood engrafted MISTRG-SKI mice. a Flow cytometric analysis depicting the total absolute cell numbers of B-lymphoid ($CD45^+CD34^-CD19^+$), Monocyte ($CD45^+CD34^-CD14^+CD16^+CD33^+/CD123^+$), Myeloid ($CD45^+CD34^-CD13^+CD33^+/CD123^+$), and plasmacytoid dendritic cell (pDC) ($CD45^+CD34^-CD4^+CD45RA^+CD11c^-MHC\text{-}II^+CD304^+CD123^+$) graft in the peripheral blood (PB) of healthy donor (HD) $CD34^+$ cord blood (CBMC) engrafted mice pre- (day 0) and post-infusion (day 7) with 5 x 10^6 anti-CD123 CAR T cells (*n*=2) or NTD T cells (*n*=3). **b** Flow cytometric analysis depicting the absolute cell numbers (cells/µL) of B-lymphoid, monocyte, myeloid, and pDCs in the spleen and bone marrow (BM) of HD $CD34^+$ CBMC engrafted mice 14 days following infusion with 5 x 10^6 anti-CD123 CAR (*n*=3) or NTD T cells (*n*=3). **c** Flow cytomteric analysis depicting the absolute cell numbers (cells/µL) of hematopoietic stem cells (HSCs), common myeloid progenitors (CMPs), and granulocytic macrophage progenitors (GMPs) in the BM of HD $CD34^+$ CBMC engrafted mice 14 days following infusion with 5 x 10^6 anti-CD123 CAR T (*n*=3) cells or NTD T cells (*n*=3). All data are represented as mean ± SEM. **b-c** *p*-values were calculated using two-sided unpaired student's *t*-test (Mann-Whitney).

Supplementary Figure 10

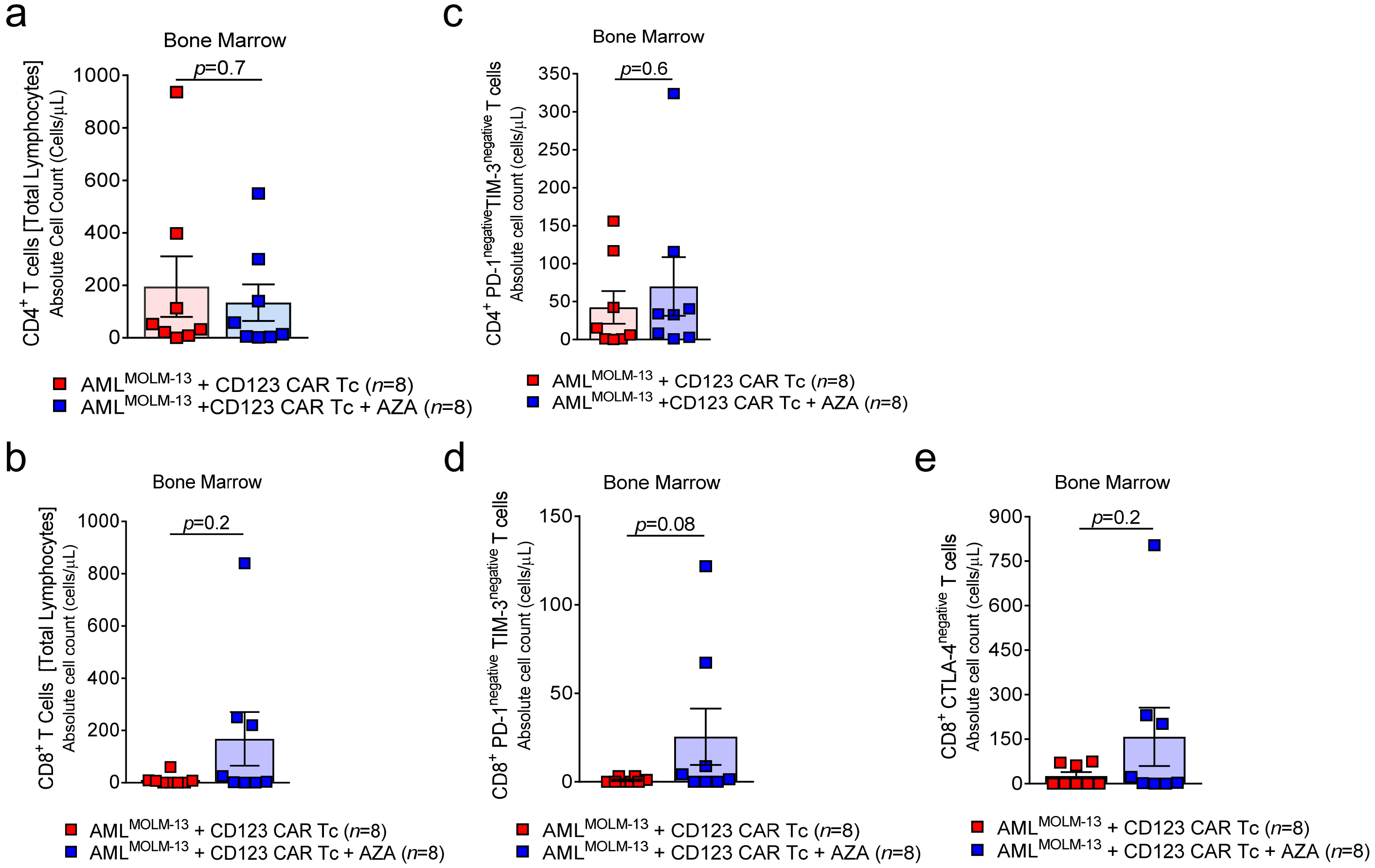

Supplementary Figure 10. Analysis of residual T cells and their expression for exhaustion markers in the bone marrow of MOLM-13 AML xenograft mice. a Scatter plot graph depicting the residual $CD4^+$ T cells in the mice treated with anti-CD123 CAR T cells only (*n*=8) versus the mice treated with anti-CD123 CAR T cells + AZA (*n*=8). **b** Scatter plot graph depicting the residual $CD8^+$ T cells in the mice treated with anti-CD123 CAR T cells only (*n*=8) versus the mice treated with anti-CD123 CAR T cells + AZA (*n*=8). Scatter plot graphs depicting the expression of residual $CD4^+$ T cells from mice treated with anti-CD123 CAR T cell only (*n*=8) versus anti-CD123 CAR T cells + AZA (*n*=8) that were **c** PD-$1^{negative}$ TIM-$3^{negative}$. Scatter plot graphs depicting the expression of residual $CD8^+$ T cells from mice treated with CD123 CAR T cell only (*n*=8) versus anti-CD123 CAR T cells + AZA (*n*=8) that were **d** PD-$1^{negative}$ TIM-$3^{negative}$ and **e** CTLA-$4^{negative}$. All data were pooled from 2 independent experiments and presented as mean absolute cell count (cells/µL) ± SEM. *p*-values were calculated using two-sided unpaired student's *t*-test (Mann-Whitney).

Supplementary Figure 11

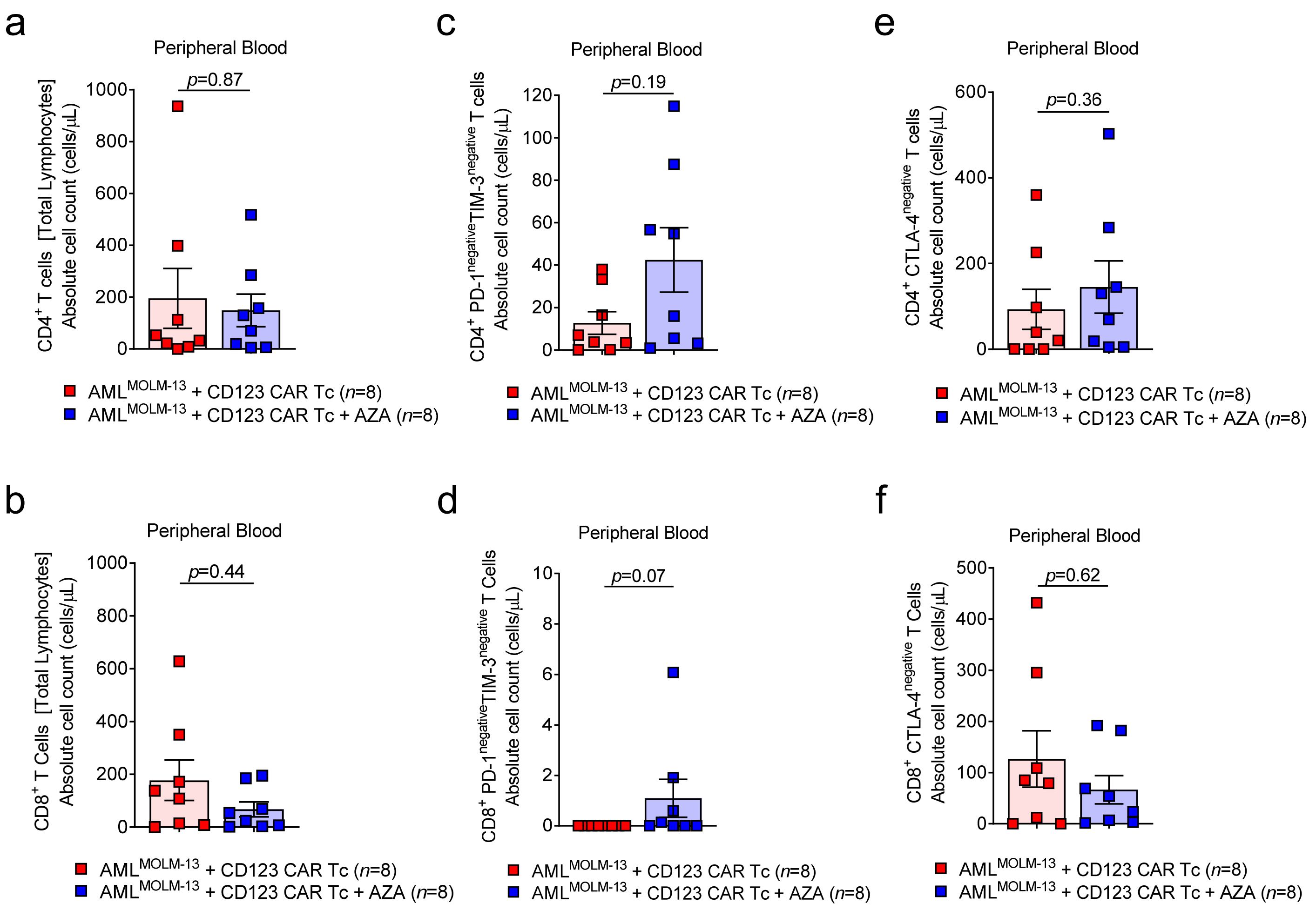

Supplementary Figure 11. Analysis of residual T cells and their expression for exhaustion markers in the peripheral blood of MOLM-13 AML xenograft mice. a Scatter plot graph depicting the residual $CD4^+$ T cells in the mice treated with anti-CD123 CAR T cells only (*n*=8) versus the mice treated with anti-CD123 CAR T cells + AZA (*n*=8). **B** Scatter plot graph depicting the residual $CD8^+$ T cells in the mice treated with anti-CD123 CAR T cells only (*n*=8) versus the mice treated with anti-CD123 CAR T cells + AZA (*n*=8). Scatter plot graphs depicting the expression of residual $CD4^+$ T cells from mice treated with anti-CD123 CAR T cell only (*n*=8) versus anti-CD123 CAR T cells + AZA (*n*=8) that were **c** PD-$1^{negative}$ TIM-$3^{negative}$. Scatter plot graphs depicting the expression of residual $CD8^+$ T cells from mice treated with anti-CD123 CAR T cell only (*n*=8) versus anti-CD123 CAR T cells + AZA (*n*=8) that were **d** PD-$1^{negative}$ TIM-$3^{negative}$. Scatter plot graphs depicting the expression of residual $CD4^+$ T cells from mice treated with anti-CD123 CAR T cell only (*n*=8) versus anti-CD123 CAR T cells + AZA (*n*=8) that were **e** CTLA-$4^{negative}$. Scatter plot graphs depicting the expression of residual $CD8^+$ T cells from mice treated with anti-CD123 CAR T cell only (*n*=8) versus anti-CD123 CAR T cells + AZA (*n*=8) that were **f** CTLA-$4^{negative}$. All data were pooled from 2 independent experiments and presented as mean absolute cell count (cells/µL) ± SEM. *p*-values were calculated using two-sided unpaired student's *t*-test (Mann-Whitney).

Supplementary Figure 12

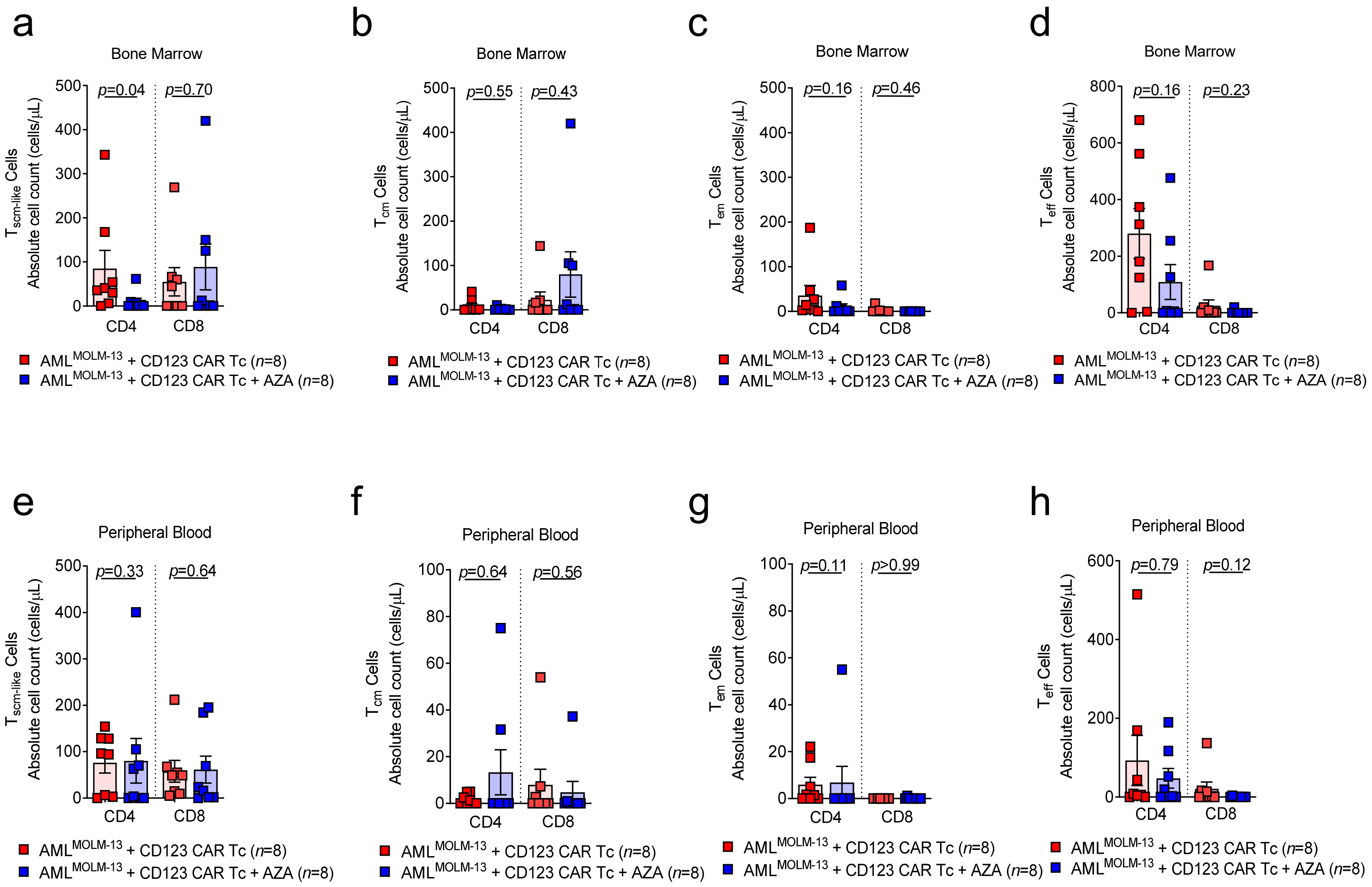

Supplementary Figure 12. Analysis of residual CD4$^+$ and CD8$^+$ T cell subsets in the bone marrow and peripheral blood of MOLM-13 AML xenograft mice. Scatter plot graphs depicting **a** T stem cell-like ($T_{scm\text{-}like}$), **b** central memory (T_{cm}), **c** effector memory (T_{em}), and **d** terminally differentiated effectors (T_{eff}) cells from residual CD4$^+$ and CD8$^+$ anti-CD123 CAR T cells in the bone marrow (BM) of mice treated with anti-CD123 CAR T cells only (n=8) versus the mice treated with anti-CD123 CAR T cells + AZA (n=8). Scatter plot graphs depicting **e** T stem cell-like ($T_{scm\text{-}like}$), **f** central memory (T_{cm}), **g** effector memory (T_{em}), and **h** terminally differentiated effectors (T_{eff}) cells from residual CD4$^+$ and CD8$^+$ anti-CD123 CAR T cells in the peripheral blood (PB) of mice treated with anti-CD123 CAR T cells only versus (n=8) the mice treated with anti-CD123 CAR T cells + AZA (n=8). All data were pooled from 2 independent experiments and presented as mean absolute cell count (cells/µL) ± SEM. p-values were calculated using two-sided unpaired student's t-test (Mann-Whitney).

Supplementary Figure 13

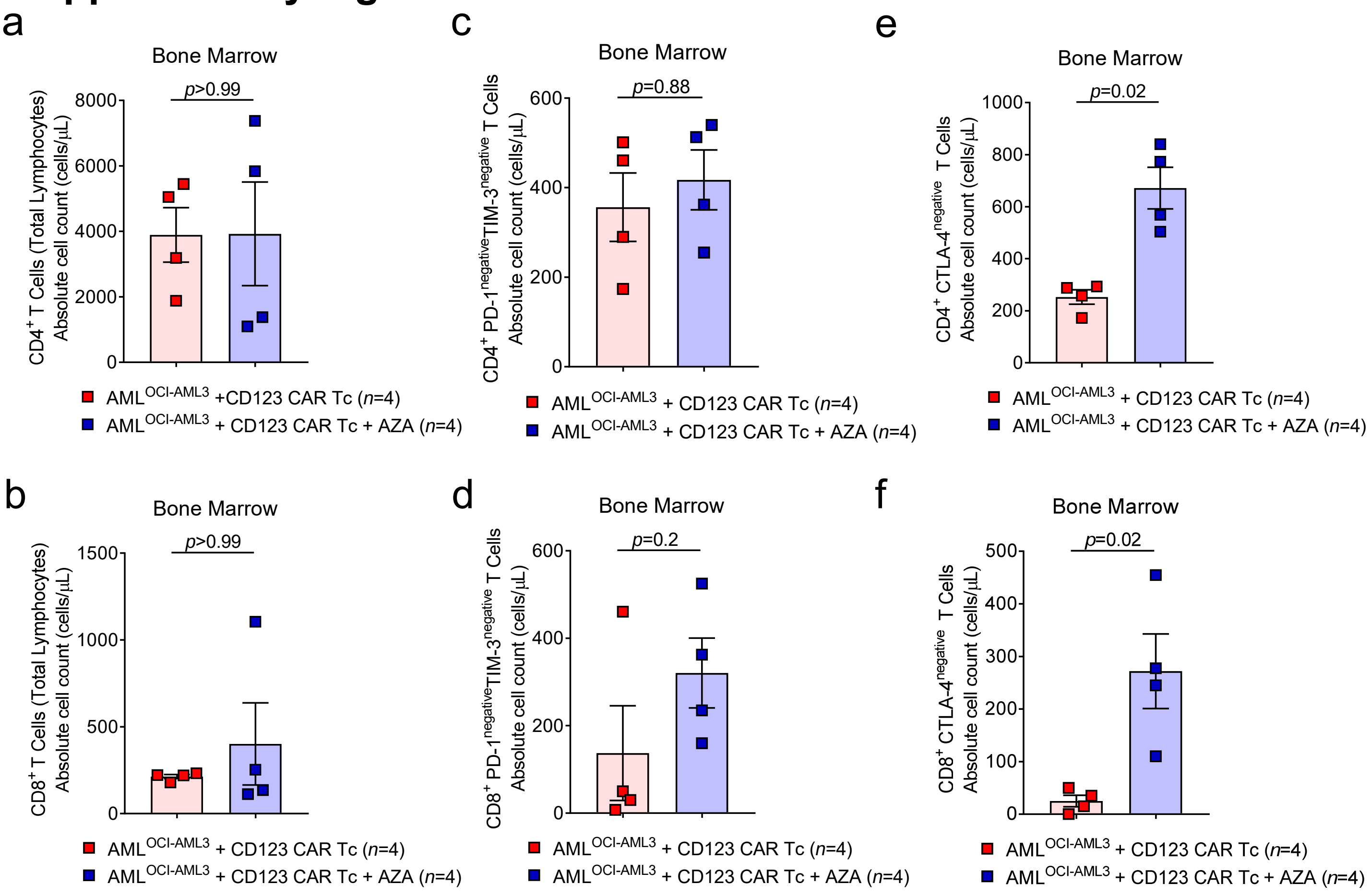

Supplementary Figure 13. Analysis of residual T cells and their expression for exhaustion markers in the bone marrow of OCI-AML3 AML xenograft mice. a Scatter plot graph depicting the residual $CD4^+$ T cells in the mice treated with anti-CD123 CAR T cells only (*n*=4) versus the mice treated with anti-CD123 CAR T cells + AZA (*n*=4). **b** Scatter plot graph depicting the residual $CD8^+$ T cells in the mice treated with anti-CD123 CAR T cells only (*n*=4) versus the mice treated with anti-CD123 CAR T cells + AZA (*n*=4). Scatter plot graphs depicting the expression of residual **c** $CD4^+$ T cells that were PD-$1^{negative}$ TIM-$3^{negative}$ or **d** $CD8^+$ T cells that were PD-$1^{negative}$ TIM-$3^{negative}$ from mice treated with anti-CD123 CAR T cell only (*n*=4) versus anti-CD123 CAR T cells + AZA (*n*=4). Scatter plot graphs depicting the expression of **e** residual $CD4^+$ T cells that were CTLA-$4^{negative}$ or **f** residual $CD8^+$ T cells that were CTLA-$4^{negative}$ from mice treated with CD123 CAR T cell only (*n*=4) versus anti-CD123 CAR T cells + AZA (*n*=4). All data are presented as mean absolute cell count (cells/μL) ± SEM. *p*-values were calculated using two-sided unpaired student's *t*-test (Mann-Whitney).

Supplementary Figure 14

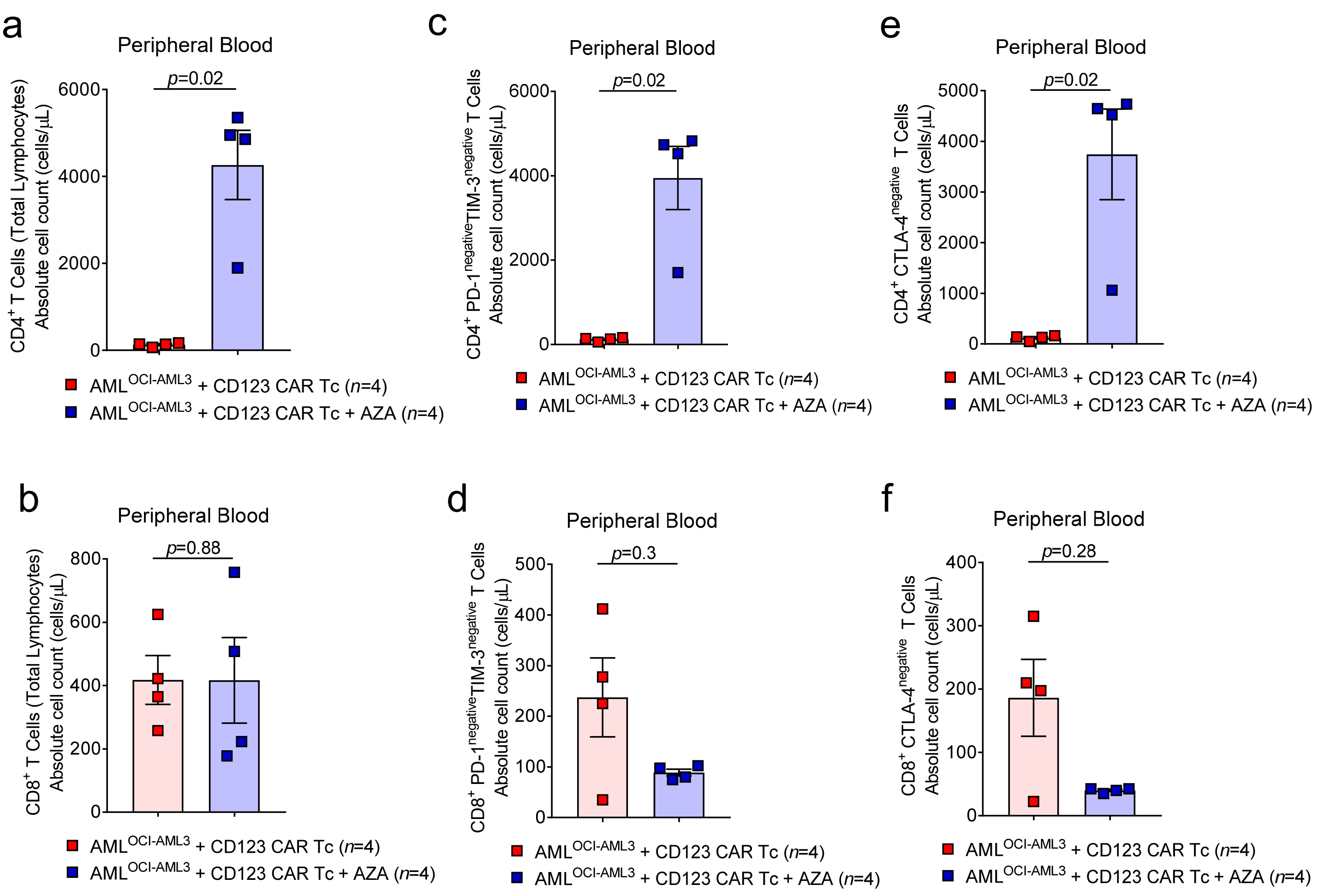

Supplementary Figure 14. Analysis of residual T cells and their expression for exhaustion markers in the peripheral blood of OCI-AML3 AML xenograft mice. Scatter plot graph depicting the residual **a** $CD4^+$ T cells or **b** $CD8^+$ T cells in the mice treated with anti-CD123 CAR T cells only versus (*n*=4) the mice treated with anti-CD123 CAR T cells + AZA (*n*=4). Scatter plot graphs depicting the expression of residual **c** $CD4^+$ T cells that were PD-$1^{negative}$ TIM-$3^{negative}$ or **d** $CD8^+$ T cells that were PD-$1^{negative}$ TIM-$3^{negative}$ from mice treated with anti-CD123 CAR T cell only (*n*=4) versus anti-CD123 CAR T cells + AZA (*n*=4). Scatter plot graphs depicting the expression of **e** residual $CD4^+$ T cells that were CTLA-$4^{negative}$ or **f** residual $CD8^+$ T cells that were CTLA-$4^{negative}$ from mice treated with CD123 CAR T cell only (*n*=4) versus anti-CD123 CAR T cells + AZA (*n*=4). All data are presented as mean absolute cell count (cells/μL) ± SEM. *p*-values were calculated using two-sided unpaired student's *t*-test (Mann-Whitney).

Supplementary Figure 15

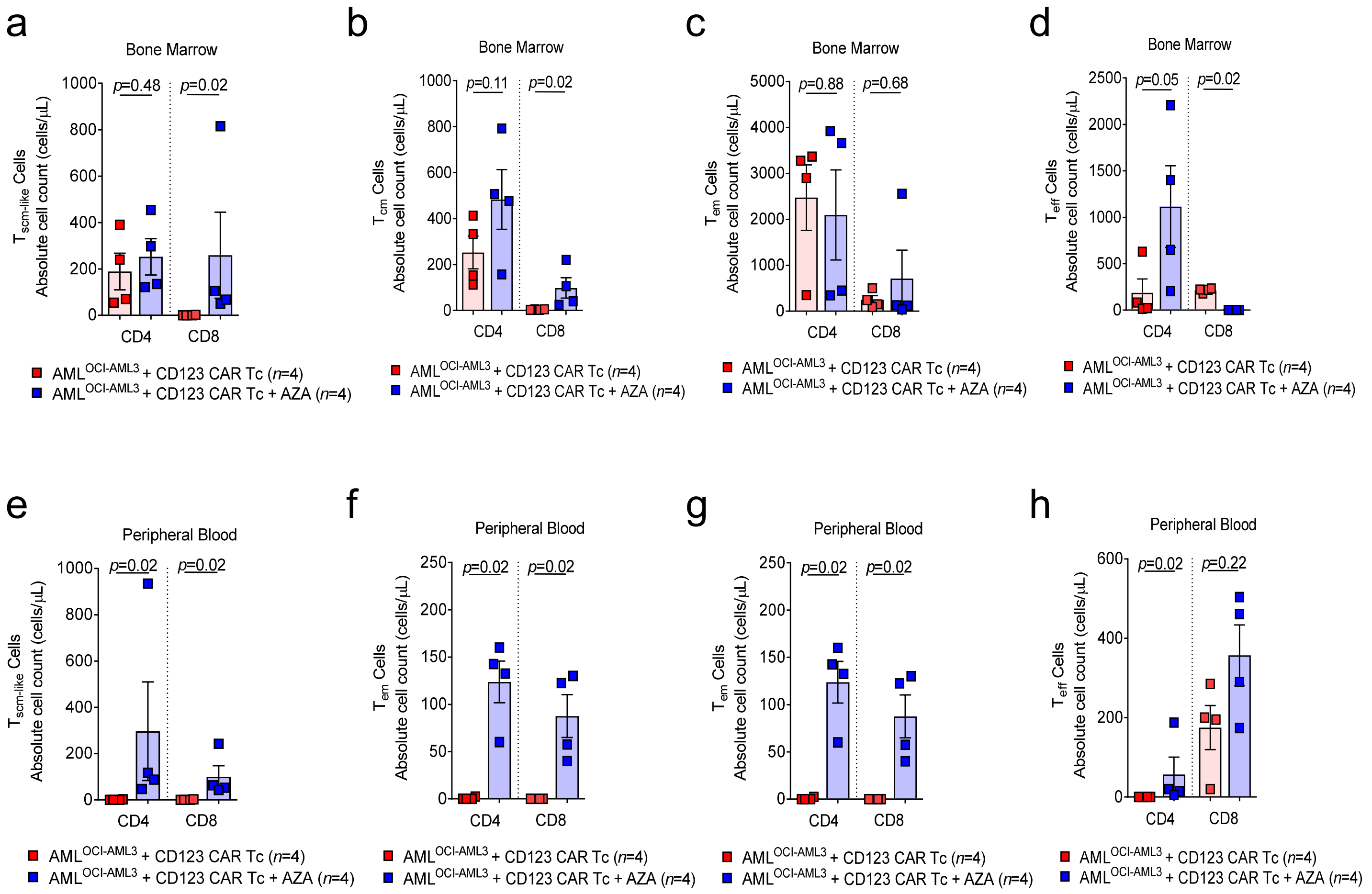

Supplementary Figure 15. Analysis of residual CD4$^+$ and CD8$^+$ T cell subsets in the bone marrow and peripheral blood of OCI-AML3 AML xenograft mice. Scatter plot graphs depicting **a** T stem cell-like ($T_{scm\text{-}like}$), **b** central memory (T_{cm}), **c** effector memory (T_{em}), and **d** terminally differentiated effectors (T_{eff}) cells from residual CD4$^+$ and CD8$^+$ anti-CD123 CAR T cells in the bone marrow (BM) of mice treated with anti-CD123 CAR T cells only (*n*=4) versus the mice treated with anti-CD123 CAR T cells + AZA (*n*=4). Scatter plot graphs depicting **e** T stem cell-like ($T_{scm\text{-}like}$), **f** central memory (T_{cm}), **g** effector memory (T_{em}), and **h** terminally differentiated effectors (T_{eff}) cells from residual CD4$^+$ and CD8$^+$ anti-CD123 CAR T cells in the peripheral blood (PB) of mice treated with anti-CD123 CAR T cells only (*n*=4) versus the mice treated with anti-CD123 CAR T cells + AZA (*n*=4). All data are presented as mean absolute cell count (cells/μL) ± SEM. *p*-values were calculated using two-sided unpaired student's *t*-test (Mann-Whitney).

Supplementary Figure 16

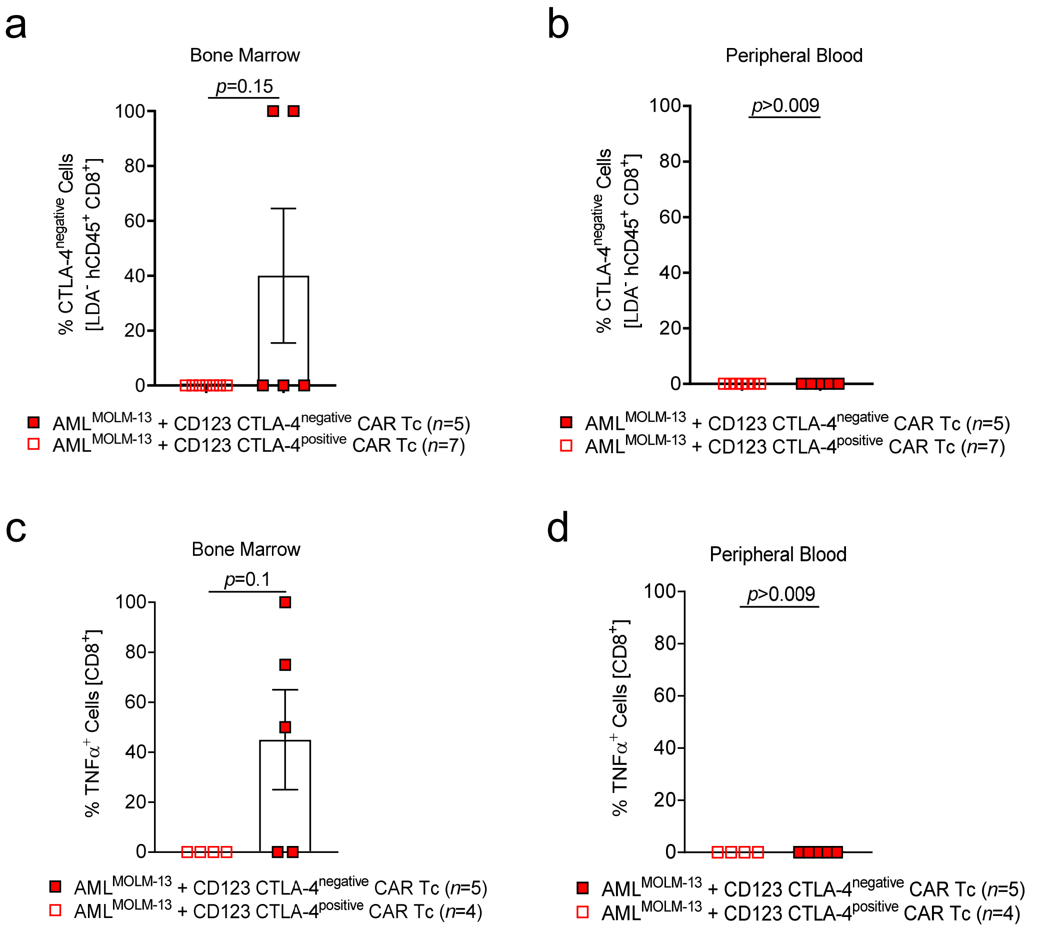

Supplementary Figure 16. Analysis of CTLA-4^{negative} and TNFα expression on residual CD8^{+} T cells in the bone marrow and peripheral blood of MOLM-13 AML xenograft mice treated with CTLA-4^{negative} or CTLA-4^{positive} anti-CD123 CAR T cells. Scatter plot graphs depicting the CTLA-4^{negative} expression of residual CD8^{+} T cells in **a** bone marrow (BM) and **b** peripheral blood (PB) of mice treated with CTLA-4^{negative} (n=5) or CTLA-4^{positive} (n=7) anti-CD123 CAR T cells. Scatter plot graphs depicting the TNFα expression of residual CD8^{+} T cells in the **c** BM and **d** PB of mice treated with CTLA-4^{negative} (n=5) or CTLA-4^{positive} (n=4) anti-CD123 CAR T cells. All data were pooled from 2 independent experiments and presented as mean ± SEM. *p*-values were calculated using two-sided unpaired student's *t*-test (Mann-Whitney).

Supplementary Figure 17

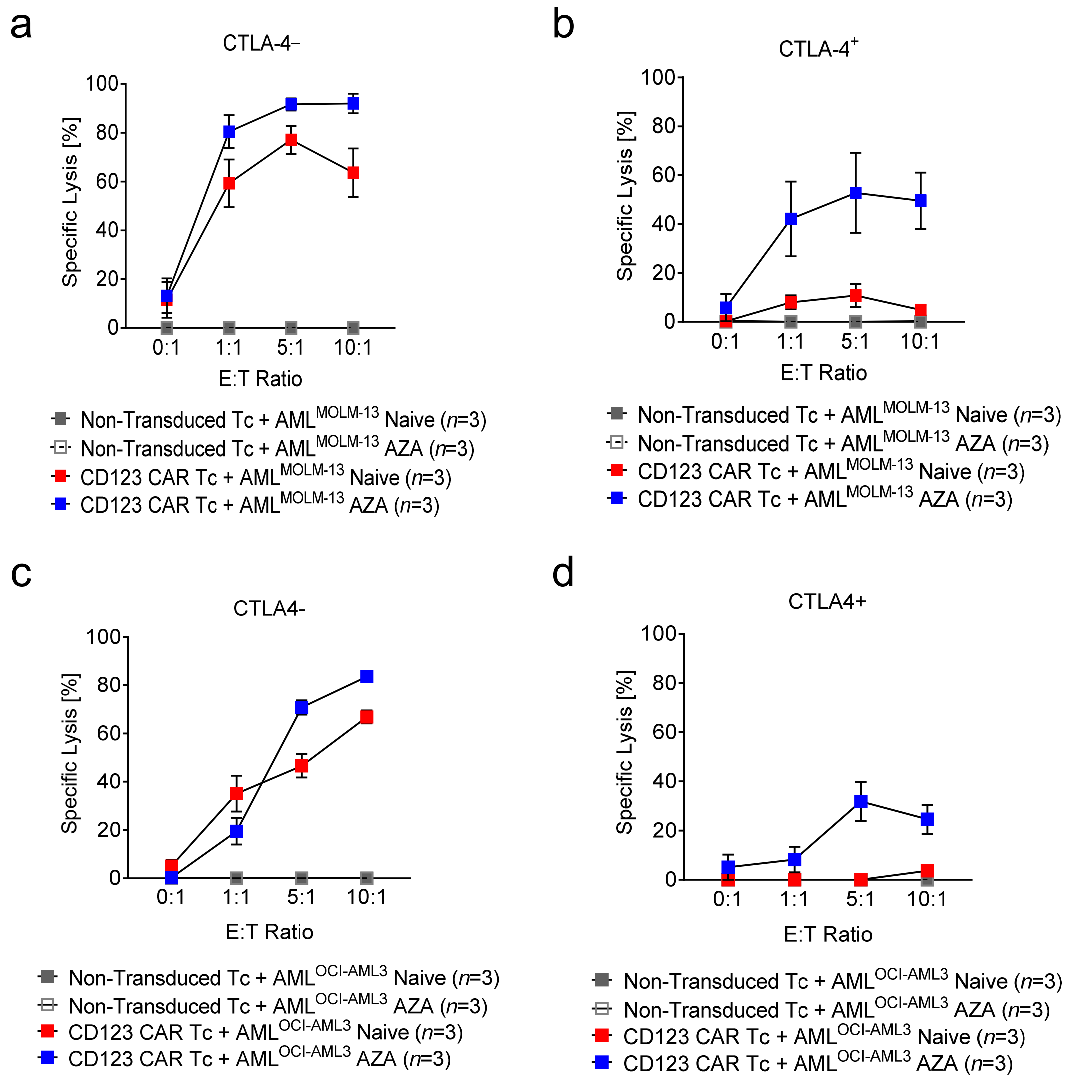

Supplementary Figure 17. CTLA-4$^+$ and CTLA-4$^-$ CD123 CAR T cells demonstrate higher lytic capacity against AZA treated AML cells compared to untreated AML cells. Specific cytotoxicity of **a** CTLA-4$^-$ and **b** CTLA-4$^+$ anti-CD123 CAR T cells (isolated from primary AML engrafted recipient mice) against untreated (*n*=3) or AZA pre-treated (*n*=3) MOLM-13 cells (CellTrace^{TM} violet labelled) by flow cytometric analysis following a 16 h co-incubation. Non-transduced (NTD) T cells were used as a control (*n*=3). Specific cytotoxicity of **c** CTLA-4$^-$ and **d** CTLA-4$^+$ anti-CD123 CAR T cells (isolated from primary AML engrafted recipient mice) against untreated (*n*=3) or AZA pre-treated (*n*=3) OCI-AML3 cells (CellTrace^{TM} violet labelled) by flow cytometric analysis following a 16 h co-incubation. Non-transduced (NTD) T cells were used as a control (*n*=3). Assay was performed in triplicate with a fixed number of target cells/well for all E:T ratios. Counting beads were used to quantify the absolute number of residual live target cells at the end of the co-culture. Residual live target cells were CellTrace violet$^+$ 7-AAD$^-$. All graphed data are presented as mean ± SEM.

Supplementary Figure 18

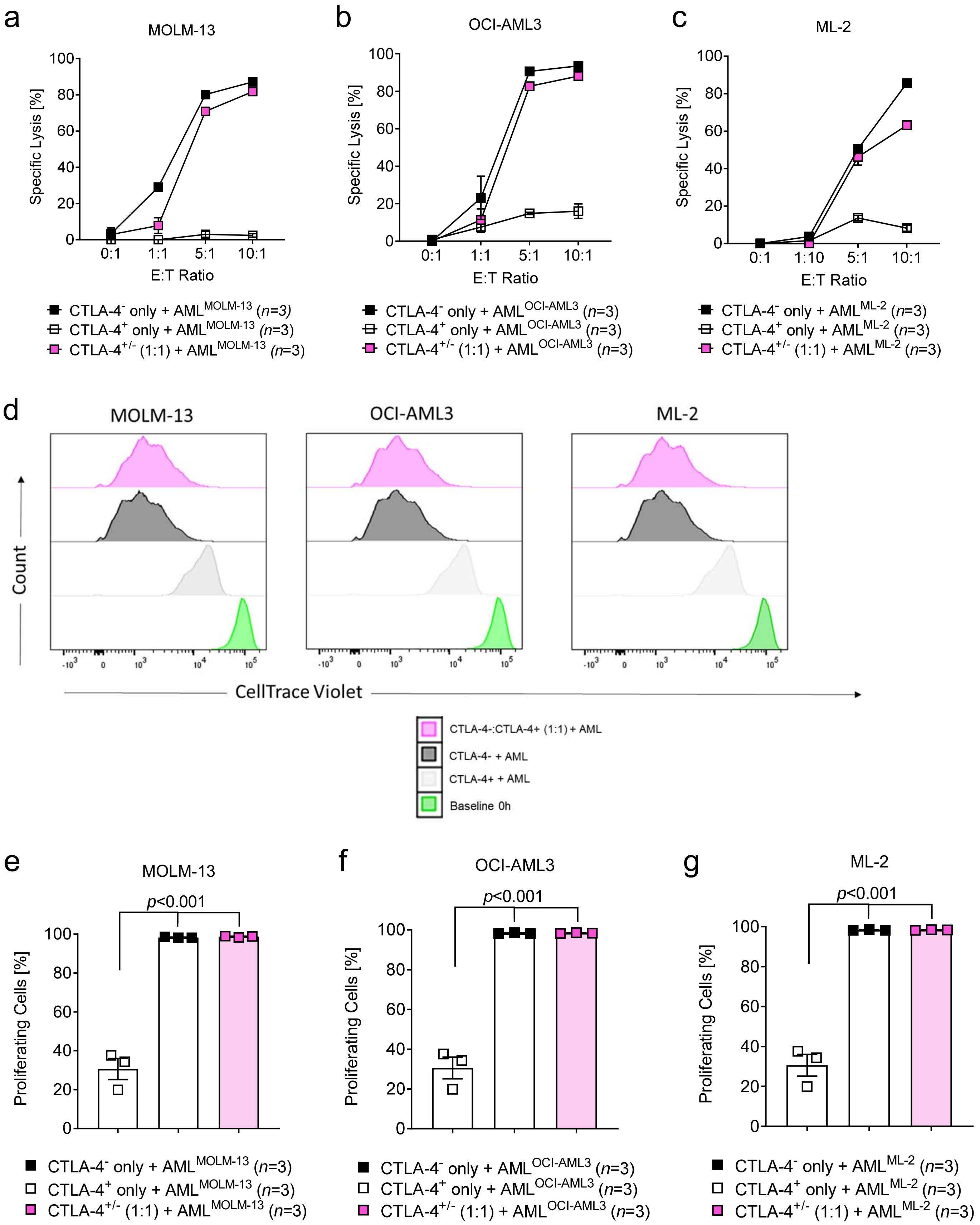

Supplementary Figure 18. Influence of CTLA-4$^+$ anti-CD123 CAR T cells on CTLA-4$^-$ anti-CD123 CAR T cell function. Specific cytotoxicity of CTLA-4$^+$ (*n*=3), CTLA-4$^-$ (*n*=3), or CTLA-4$^{+/-}$ (1:1) (*n*=3) anti-CD123 CAR T cells (isolated from primary AML engrafted recipient mice) against CellTrace^{TM} violet labelled **a** MOLM-13 cells, **b** OCI-AML3 cells, and **c** ML-2 cells by flow cytometric analysis following a 16 h co-incubation. Data were pooled from 3 independent experiments with a fixed number of target cells/well for all E:T ratios. Counting beads were used to quantify the absolute number of residual live target cells at the end of the co-culture. Residual live target cells were CellTrace violet$^+$ 7-AAD$^-$. **d** Representative histograms depicting the proliferation of CTLA-4$^-$ (dark grey), CTLA-4$^+$ (light grey), and CTLA-4$^{+/-}$ (1:1) (purple) anti-CD123 CAR T cells examined by CellTrace^{TM} violet dye dilution following a 96 h co-culture with MOLM-13, OCI-AML3 and ML-2 cells. For the condition where CTLA-4$^{+/-}$ cells were mixed at an E:T ratio, only CTLA-4$^-$ anti-CD123 CAR T cells were CellTrace^{TM} violet labelled. Scatter plot graphs denoting the total percentage of proliferating CTLA-4$^-$ (*n*=3), CTLA-4$^+$ (*n*=3), and CTLA-4$^{+/-}$ (1:1) (*n*=3) anti-CD123 CAR T cells following a 96 h co-culture with **e** MOLM-13, **f** OCI-AML3, and **g** ML-2 AML cells. Data were pooled from 3 independent experiments. All graphed data are presented as mean ± SEM. *p*-values for were calculated by two-sided one-way ANOVA (Kruskal-Wallis test with Dunn's multiple comparison)**.**

Supplementary Figure 19

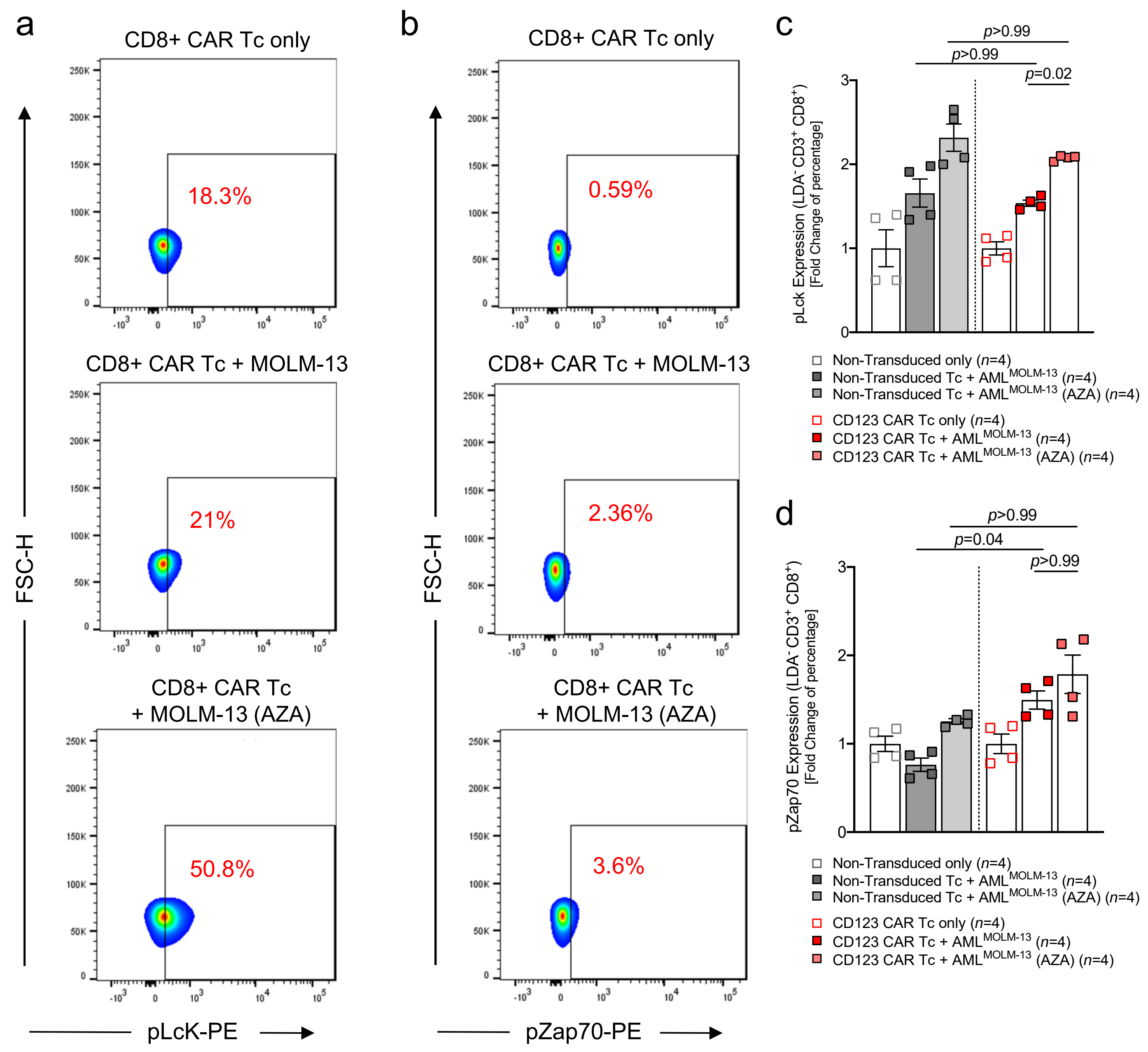

Supplementary Figure 19. Assessment of the phosphorylation of intracellular Lck and Zap70 in CD8$^+$ anti-CD123 CAR T cells in the presence of MOLM-13 AML cells. a Representative flow cytometry plot depicting the phosphorylated level of LcK (pLck) in CD8$^+$ anti-CD123 CAR T cells that have been exposed for 96 h to media only, naive MOLM-13 cells, or MOLM-13 cells pre-treated with 1μM AZA. **b** Representative flow cytometry plot depicting the phosphorylated level of Zap70 (pZap70) in CD8$^+$ anti-CD123 CAR T cells that have been exposed for 96 h to media only, naive MOLM-13 cells, or MOLM-13 cells pre-treated with 1μM AZA. **c** Scatter plot depicting the fold change in percentage expression of pLcK in CD4$^+$ anti-CD123 CAR T cells that have been exposed for 96 h to media only (*n*=4), naive MOLM-13 cells(*n*=4), or MOLM-13 cells pre-treated with 1μM AZA (*n*=4). Data were pooled from 4 independent experiments. **d** Scatter plot depicting the fold change in percentage expression of pZap70 in CD4$^+$ anti-CD123 CAR T cells that have been exposed for 96 h to media only (*n*=4), naive MOLM-13 cells (*n*=4), or MOLM-13 cells pre-treated with 1μM AZA (*n*=4). Data were pooled from 4 independent experiments. All graphed data are represented as mean ± SEM. *p*-values were calculated using two-sided unpaired student's *t*-test (Mann-Whitney).

Supplementary Table 1: AML Patient Characteristics (SAHMRI, Adelaide, Australia)

Patient	Cytogenetics	Risk Group
**1**	Normal	Intermediate
**2**	Normal	Intermediate
**3**	Normal	Intermediate
**4**	Normal	Intermediate
**5**	Monosomy 7q	Poor
**6**	Normal	Intermediate
**7**	Normal	Intermediate
**8**	Trisomy 8	Intermediate
**9**	Normal	Intermediate
**10**	Normal	Intermediate
**11**	t(5,10); tri21	Intermediate
**12**	Trisomy 8, trisomy 19, 11q- and marker chromosome X 3	Poor
**13**	Normal	Intermediate
**14**	Del9q; trisomy21	Intermediate
**15**	Normal	Intermediate
**16**	Normal	Intermediate
**17**	Normal	Intermediate
**18**	Normal	Intermediate
**19**	Normal	Intermediate
**20**	Normal	Intermediate
**21**	11q23 rearranged, trisomy 21	Intermediate
**22**	Del6q	Intermediate
**23**	Normal	Intermediate
**24**	Normal	Intermediate
**25**	$Flt3\text{-}ITD^+$	Poor
**26**	Trisomy 8	Intermediate
**27**	$Flt3\text{-}ITD^+$	Poor
**28**	$Flt3\text{-}ITD^+$	Poor
**29**	Normal	Intermediate
**30**	Normal	Intermediate
**31**	Del5q; Monosomy 7	Poor
**32**	Trisomy 11	Intermediate

*Blast counts (%) for patients were not available in the database.

Supplementary Table 2: AML Patient Characteristics (University Medical Clinic, Freiburg, Germany)

Patient	Cytogenetics	% Blast Count PB	% Blast Count BM	Blast phenotype
**1**	V617F (JAK2)	94	85	$CD34^{+}$ CD117-
**2**	Normal	0	0.3	$CD34^{+}$ CD117-
**3**	21q22/RUNX1 mutation	12	71	$CD34^{+}$ CD117-
**4**	ASXL-1, DNMT3A, IDH1, PHF6, RUNX1 mutations	90	75	$CD34^{+}$ CD117-
**5**	5q31/5q33 (EGR1); KMT2A 11q23 rearrangement; t(8;21) RUNX1 mutation	4	43	$CD34^{+}$
**6**	NPM1 mutation	13	Not available	$CD117^{+}$
**7**	Monosomy 7; t(8;21)	50	74	$CD34^{+}$
**8**	Normal	77	93	$CD117^{+}$
**9**	IDH2, IKZF1, NRAS, TET-2 mutations	>95	80	$CD34^{+}$
**10**	Normal	Not available	Not available	Not available
**11**	CBL, TP53 mutations	35	2.5	$CD34^{+}$ $CD117^{+}$
**12**	PTPN11 (exon 13) mutation	13	Not available	$CD34^{+}$ $CD117^{+}$

Supplementary Table 3: Antibodies for flow cytometry

Antibody	Clone	Catalogue number	Fluorochrome	Vendor
Anti-human CD3	SK7	344824	Pacific Blue	Biolegend
Anti-human CD3	UCHT1	560835	PerCP-Cy5.5	BD Bioscience
Anti-human CD3	OKT3	317333	PeCy7	Biolegend
Anti-human CD3	HIT3a	300312	APC	Biolegend
Anti-human CD3	HIT3a	300306	FITC	Biolegend
Anti-human CD4	SK3	11-0047-42	FITC	eBioscience
Anti-human CD4	OKT4	317436	BV650	Biolegend
Anti-human CD95	DX2	555674	PE	BD Bioscience
Anti-human CD8	RPA-T8	555369	APC	BD Bioscience
Anti-human CD8	BW135/80	130-113-162	Pacific Blue	Miltenyi Biotec
Anti-human CD8	RPA-T8	563677	BV711	BD Bioscience
Anti-human CD45RO	UCHL1	25-0427-42	PeCy7	eBioscience
7-Aminoactinomycin D (7-AAD)	N/A	559925	PerCP-Cy5.5	BD Bioscience
Anti-human CD27	0323	47-0279-42	APC-ef780	eBioscience
Anti-human CD45RA	HI100	304138	BV711	Biolegend
Anti-human CD45RA	HI100	560675	PeCy7	BD Bioscience
Anti-human CD45	2D1	347463	FITC	BD Bioscience
Anti-human CD45	2D1	560178	APC-H7	BD Bioscience
Anti-human CD123	7G3	558714	PerCP-Cy5.5	BD Bioscience
Anti-human CD123	7G3	560826	PeCy7	BD Bioscience
Anti-human CD279 (PD-1)	EH12.2H7	329904	FITC	Biolegend
Anti-human CD279 (PD-1)	EH12.1	560795	PE	BD Bioscience
Anti-human CD152 (CTLA-4)	BN13	563931	BV786	BD Bioscience
Anti-human CD152 (CTLA-4)	L3D10	349908	APC	Biolegend
Anti-human CD152 (CTLA-4)	14D3	11-1529-42	FITC	BD Bioscience
Anti-human CD336 (TIM-3)	7D3	565564	BV650	BD Bioscience

Anti-human CD223 (LAG-3)	T47-530	565716	Alexa Flour 647	BD Bioscience
Live Dead Aqua	N/A	555516	V500/Amcyan	Invitrogen/Thermo Fischer Scientific
Anti-human Lin-Cocktail (CD3, CD14, CD16, CD19, CD20, CD56)	UCHT1;HCD14;3G8;HIB19:2H7:HCD56	348805	Pacific Blue	Biolegend
Anti-human CD38	LS198-4-3	A99022	ECD	Beckman Coulter
Anti-human CD34	581	561440	Alexa Fluor 700	BD Bioscience
Anti-human CD13	WM15	561599	PeCy7	BD Bioscience
Anti-human CD33	WM53	561157	V450	BD Bioscience
Anti-human CD19	HIB19	302234	BV421	Biolegend
Anti-human CD11c	3.9	301610	PeCy5	Biolegend
Anti-human CD304	12C2	354504	PE	Biolegend
Anti-human CD14	TuK4	MHCD1417	PE Texas Red	Life Technologies
Anti-human HLA-DR	TU36	MHLDR17	PE Texas Red	Life Technolgies
Anti-human CD117 (c-kit)	104D2	332785	PE	BD Biosciences
Anti-human CD107a	H4A3	555800	FITC	BD Bioscience
Streptavidin-Conjugated PE	N/A	349023	PE	BD Bioscience
Pierce Biotinylated recombinant Protein-L	N/A	21189	N/A	Thermo Fischer Scientific
Anti-human TNFα	Mab11	502909	PE	Biolegend
Anti-human IFN-γ	4S.B3	502512	APC	Biolegend

Supplementary Table 4: Immune response genes and associated transcripts depicted in Supplementary Figure 6

Gene name	gene ID
IRF1	ENSG00000125347
IRF2	ENSG00000168310
IRF3	ENSG00000126456
IRF4	ENSG00000137265
IRF5	ENSG00000128604
IRF6	ENSG00000117595
IRF8	ENSG00000140968
IRF9	ENSG00000213928
IFNAR1	ENSG00000142166
IFNAR2	ENSG00000159110
IL1A	ENSG00000115008
IL1B	ENSG00000125538
IL1F10	ENSG00000136697
IL1RN	ENSG00000136689
IL2	ENSG00000109471
IL3	ENSG00000164399
IL4	ENSG00000113520
IL5	ENSG00000113525
IL6	ENSG00000136244
IL7	ENSG00000104432
CXCL8	ENSG00000169429

Gene name	gene ID
TNFRSF1A	ENSG00000067182
TNFRSF1B	ENSG00000028137
TNFRSF4	ENSG00000186827
CD40	ENSG00000101017
TNFRSF6B	ENSG00000243509
CD27	ENSG00000139193
TNFRSF8	ENSG00000120949
TNFRSF9	ENSG00000049249
TNFRSF10A	ENSG00000104689
TNFRSF10B	ENSG00000120889
TNFRSF10C	ENSG00000173535
TNFRSF10D	ENSG00000173530
TNFRSF11A	ENSG00000141655
TNFRSF11B	ENSG00000164761
TNFRSF12A	ENSG00000006327
TNFRSF13B	ENSG00000240505
TNFRSF13C	ENSG00000159958
NGFR	ENSG00000064300
TNFRSF17	ENSG00000048462
TNFRSF18	ENSG00000186891
TNFRSF19	ENSG00000127863
TNFRSF21	ENSG00000146072
TNFRSF25	ENSG00000215788
CD274	ENSG00000120217
