# Supplementary material for: Demethylating therapy increases anti-CD123 CAR T cell cytotoxicity against acute myeloid leukemia
Source: Nat Commun. 2021 Nov 8;12:6436. doi: 10.1038/s41467-021-26683-0 (PMC8575966; doi:10.1038/s41467-021-26683-0)
Supplement: Supplementary file 2 — Reporting summary [file 41467_2021_26683_MOESM2_ESM.pdf]

nature research

Corresponding author(s): Robert Zeiser
Last updated by author(s): Oct 2, 2021

Reporting Summary

Nature Research wishes to improve the reproducibility of the work that we publish. This form provides structure for consistency and transparency in reporting. For further information on Nature Research policies, see Authors & Referees and the Editorial Policy Checklist .

Statistics

For all statistical analyses, confirm that the following items are present in the figure legend, table legend, main text, or Methods section.

n/a	Confirmed	
☐	☒	The exact sample size (*n*) for each experimental group/condition, given as a discrete number and unit of measurement
☐	☒	A statement on whether measurements were taken from distinct samples or whether the same sample was measured repeatedly
☐	☒	The statistical test(s) used AND whether they are one- or two-sided<br>*Only common tests should be described solely by name; describe more complex techniques in the Methods section.*
☒	☐	A description of all covariates tested
☐	☒	A description of any assumptions or corrections, such as tests of normality and adjustment for multiple comparisons
☐	☒	A full description of the statistical parameters including central tendency (e.g. means) or other basic estimates (e.g. regression coefficient) AND variation (e.g. standard deviation) or associated estimates of uncertainty (e.g. confidence intervals)
☐	☒	For null hypothesis testing, the test statistic (e.g. *F*, *t*, *r*) with confidence intervals, effect sizes, degrees of freedom and *P* value noted<br>*Give P values as exact values whenever suitable.*
☒	☐	For Bayesian analysis, information on the choice of priors and Markov chain Monte Carlo settings
☒	☐	For hierarchical and complex designs, identification of the appropriate level for tests and full reporting of outcomes
☒	☐	Estimates of effect sizes (e.g. Cohen's *d*, Pearson's *r*), indicating how they were calculated

Our web collection on statistics for biologists contains articles on many of the points above.

Software and code

Policy information about availability of computer code

Data collection	No software was used for data collection.
Data analysis	Statistical analysis was performed using Graphpad Prism version 7.01 or 8.2.1. Flow cytometry data was analyzed using FlowJo software (Flowjo v10.4 or v10.6). WGS: library preperation was performed using Ovation Ultralow Methyl-Seq DR Multiplex System (NuGEN/ TECAN). Library quantification and fragment size estimation of the finished libraries was performed with Qubit dsDNA HS Assay Kit (ThermoFisher) and Agilent High Sensitivity DNA Kit (Agilent), respectively. Sequencing data was mapped to human genome assembly hg38 using bwameth. Reads were trimmed with Trim Galore (https://github.com/FelixKrueger/TrimGalore) prior mapping. Extraction of methylation data was done with MethylDackel (https://github.com/dpryan79/MethylDackel). MethylseekR software was used for segmentation of the DNA methylation data.<br>In Vivo Bioluminescent imaging data was quantified using Living Image v4.7.3.

For manuscripts utilizing custom algorithms or software that are central to the research but not yet described in published literature, software must be made available to editors/reviewers. We strongly encourage code deposition in a community repository (e.g. GitHub). See the Nature Research guidelines for submitting code & software for further information.

Data

Policy information about availability of data

All manuscripts must include a data availability statement. This statement should provide the following information, where applicable:

- Accession codes, unique identifiers, or web links for publicly available datasets
- A list of figures that have associated raw data
- A description of any restrictions on data availability

The RNA-sequencing and whole genome bisulfite sequencing data in this study have been deposited in the GEO repository under accession code GSE184891 (https://www.ncbi.nlm.nih.gov/geo/query/acc.cgi?acc=GSE184891) and BioProject repository under accession code PRJNA766490 (https://

dataview.ncbi.nlm.nih.gov/object/PRJNA766490?reviewer=jvclq763e4cg7bptp553ikjbks), respectively. All other processed data generated in this study as depicted in the main and supplementary figures are provided as a source data file.

Field-specific reporting

Please select the one below that is the best fit for your research. If you are not sure, read the appropriate sections before making your selection.

[x] Life sciences [ ] Behavioural & social sciences [ ] Ecological, evolutionary & environmental sciences

For a reference copy of the document with all sections, see nature.com/documents/nr-reporting-summary-flat.pdf

Life sciences study design

All studies must disclose on these points even when the disclosure is negative.

Sample size	For the sample size throughout vivo experiments a sample size of at least n=5 per group was used to allow for accurate statistical analyses and comparisons across groups . For In Vitro experiments, a sample size of at least n=3 was used to allow for proper statistcal analyses of the data.
Data exclusions	No data were excluded from the analyses.
Replication	All the experimental findings were reliably reproduced. All experiments were performed independently at least 3 times with each condition set up with at least technical duplicates.
Randomization	For In vivo experiments, mice were first imaged using BLI to confirm engraftment. Thereafter, mice with similar engraftment levels were randomized into the experimental treatment groups. All samples or mice were included in our analysis.
Blinding	The experiments were performed in a non-blinded manner. Only tissue damage histological scoring was done in a blinded setting. In order to obtain unbiased data, the histopathological scoring and analysis was performed by a pathologist blinded to the treatment groups. Only after finalization of the quantitative scores, the samples were allocated to their designated groups.

Reporting for specific materials, systems and methods

We require information from authors about some types of materials, experimental systems and methods used in many studies. Here, indicate whether each material, system or method listed is relevant to your study. If you are not sure if a list item applies to your research, read the appropriate section before selecting a response.

Materials & experimental systems

n/a	Involved in the study	
[ ]	[x]	Antibodies
[ ]	[x]	Eukaryotic cell lines
[x]	[ ]	Palaeontology
[ ]	[x]	Animals and other organisms
[ ]	[x]	Human research participants
[x]	[ ]	Clinical data

Methods

n/a	Involved in the study	
[x]	[ ]	ChIP-seq
[ ]	[x]	Flow cytometry
[x]	[ ]	MRI-based neuroimaging

Antibodies

Antibodies used

Anti-human CD3 SK7 344824 Pacific Blue Biolegend
Anti-human CD3 UCHT1 560835 PerCP-Cy5.5 BD Bioscience
Anti-human CD3 OKT3 317333 PeCy7 Biolegend
Anti-human CD3 HIT3a 300312 APC Biolegend
Anti-human CD3 HIT3a 300306 FITC Biolegend
Anti-human CD4 SK3 11-0047-42 FITC eBioscience
Anti-human CD4 OKT4 317436 BV650 Biolegend
Anti-human CD95 DX2 555674 PE BD Bioscience
Anti-human CD8 RPA-T8 555369 APC BD Bioscience
Anti-human CD8 BW135/80 130-113-162 Pacific Blue Miltenyi Biotec
Anti-human CD8 RPA-T8 563677 BV711 BD Bioscience
Anti-human CD45RO UCHL1 25-0427-42 PeCy7 eBioscience
7-Aminoactinomycin D (7-AAD) N/A 559925 PerCP-Cy5.5 BD Bioscience
Anti-human CD27 0323 47-0279-42 APC-ef780 eBioscience
Anti-human CD45RA HI100 304138 BV711 Biolegend
Anti-human CD45RA HI100 560675 PeCy7 BD Bioscience

Anti-human CD45 2D1 347463 FITC BD Bioscience
Anti-human CD45 2D1 560178 APC-H7 BD Bioscience
Anti-human CD123 7G3 558714 PerCP-Cy5.5 BD Bioscience
Anti-human CD123 7G3 560826
PeCy7 BD Bioscience
Anti-human CD279 (PD-1) EH12.2H7 329904 FITC Biolegend
Anti-human CD279 (PD-1) EH12.1 560795 PE BD Bioscience
Anti-human CD152 (CTLA-4) BN13 563931
BV786 BD Bioscience
Anti-human CD152 (CTLA-4) L3D10 349908 APC Biolegend
Anti-human CD152 (CTLA-4) 14D3 11-1529-42 FITC BD Bioscience
Anti-human CD336 (TIM-3) 7D3 565564 BV650 BD Bioscience
Anti-human CD223
(LAG-3) T47-530 565716 Alexa Flour 647 BD Bioscience
Live Dead Aqua N/A 555516 V500/Amcyan Invitrogen/Thermo Fischer Scientific
Anti-human Lin- Cocktail (CD3, CD14, CD16, CD19, CD20, CD56) UCHT1;HCD14;3G8;HIB19:2H7:HCD56 348805 Pacific Blue Biolegend
Anti-human CD38 LS198-4-3 A99022 ECD Beckman Coulter
Anti-human CD34 581 561440 Alexa Fluor 700 BD Bioscience

Anti-human CD13 WM15 561599 PeCy7 BD Bioscience
Anti-human CD33 WM53 561157 V450 BD Bioscience
Anti-human CD19 HIB19 302234 BV421 Biolegend
Anti-human CD11c 3.9 301610 PeCy5 Biolegend
Anti-human CD304 12C2 354504 PE Biolegend
Anti-human CD14 TuK4 MHCD1417 PE Texas Red Life Technologies
Anti-human HLA-DR TU36 MHLDR17 PE Texas Red Life Technolgies
Anti-human CD117 (c-kit) 104D2 332785 PE BD Biosciences
Anti-human CD107a H4A3 555800 FITC BD Bioscience
Streptavidin-Conjugated PE N/A 349023 PE BD Bioscience
Pierce Biotinylated recombinant Protein-L N/A 21189 N/A Thermo Fischer Scientific
Anti-human TNFα Mab11 502909 PE Biolegend
Anti-human IFN-γ 4S.B3 502512 APC Biolegend

These information are also provided in the supplementary methods. All antibodies were used at a dilution of 1:100 except for Protein-L which was used at a conc. of 1ug/mL and streptavidin-PE was used at 1:20 dilution.

Validation

Antibodies were validated by titration experiments where the base line was the recommended working concentration provided by the manufacturer. In most cases, the manufacturer always recommended a dilution of 1:20. Validation was performed per experimental setup, and cell type that was used.

Eukaryotic cell lines

Policy information about cell lines

Cell line source(s)

Human AML cell lines:
-KG1a (ATCC)
-MOLM-13 (ATCC)
-MOLM-13 GFP-ffLuc (Max Jacob Kappenstein and Prof. Dr. Nikolas von Bubnoff-University of Freiburg, Germany)
-ML-2 (Prof. Dr. Michael Lübbert research laboratory-University of Freiburg, Germany, Commercial Source: ATCC)
-HL-60 (Prof. Dr. Michael Lübbert research laboratory-University of Freiburg, Germany, Commerical Source: ATCC)
-OCI-AML3 (Commerical Source: ATCC)

Human ALL cell lines:
-SUPB15 (as control cell line) (Leukemia research laboratory - Deborah White, SAHMRI, Adelaide, Australia, commercial source: ATCC)

-HEK-293T (ATCC)

Authentication

The cell lines used in this study were obtained from a batch that had been prevoiusly authenticated at DSMZ, Germany using PCR assays with species-specific primers.

Mycoplasma contamination

The cell lines used in this study were obtained from a batch that had been prevoiusly tested for Mycoplasma contamination and were found to be negative. Cell lines were subsequently routinely tested for mycoplasma contamination.

Commonly misidentified lines
(See ICLAC register)

No commonly misidentified cell lines were used in this study.

Animals and other organisms

Policy information about studies involving animals; ARRIVE guidelines recommended for reporting animal research

Laboratory animals	Rag2–/–Il2rγ–/– immunocompromised mice were bred in-house at the University clinic (University of Freiburg) animal facility under standard room temperatures (~26oC) with a 12 h light/dark cycle and humidity ranging 40-60%. Mice were housed in individually ventilated cages and received acidified and autoclaved water. Male and female mice were used between 8-10 weeks of age. Mice were bred and housed under specific pathogen-free (SPF) conditions in the animal facility of University Medical centre Freiburg (ZKF).<br><br>NSG (NOD-scid IL2rγnull) mice and humanized cytokine knock-in mice (CSF1h/hIL-3/CSF2h/hhSIRPAtgTPOh/hRag2-/-IL2rγ-/-), also known as MISTRG-SKI, were bred and maintained at the University Hospital Zürich animal facility according to the Swiss Federal Veterinary Office guidelines and the Cantonal Veterinary Office Zürich. Mice were housed in similar conditions as stated above.<br><br>Details are also supplied in the manuscript.
Wild animals	The study did not involve wild animals.
Field-collected samples	The study did not involve samples collected from the field.
Ethics oversight	All mouse experiments were approved by the Federal Ministry for Nature, Environment and Consumers' Protection of the state of Baden-Württemberg, Germany (Protocol numbers: G-18/019) or the Cantonal Veterinary Office Zürich (194/2018).

Note that full information on the approval of the study protocol must also be provided in the manuscript.

Human research participants

Policy information about studies involving human research participants

Population characteristics	Human samples used in the study included patients with acute myeloid leukemia (AML) that were collected at diagnosis or had relapsed/refractory disease following transplantation (Australia and Germany). Samples were obtained following written consent. Healthy Donor BM (Australia) samples were obtained following written consent from the donors. Samples were obtained from either female or males ranging who were at least of legal age (>18 years old).<br><br>Further details of the AML patients are supplied in the supplementary materials.
Recruitment	Samples from patients of interest, who acquired written informed consent at University Medical Center Freiburg and SAHMRI, Adelaide, Australia were only used. for the AML patients, written consent was obtained to take extra material during standard diagnostics testing. No other specific criteria was used for the selection of the AML samples. For the healthy donors, specimens were taken on a complete voluntary basis following the written consent. No selection criteria other than legal age was used.
Ethics oversight	Human sample collection and analysis were approved by the Institutional Ethics Review Board of the Medical center, University of Freiburg, Germany (protocol number: 509/16) and the Australian Institutional Human Research Ethics Committee (R20150526, HREC/15/RAH/221) and and the Cantonal Ethics Board Zürich, Switzerland (Ethics approval no's: 2009-0062). Written informed consent was obtained from each patient. All analysis of human data was carried out in compliance with relevant ethical regulations

Note that full information on the approval of the study protocol must also be provided in the manuscript.

Flow Cytometry

Plots

Confirm that:

- [x] The axis labels state the marker and fluorochrome used (e.g. CD4-FITC).
- [x] The axis scales are clearly visible. Include numbers along axes only for bottom left plot of group (a 'group' is an analysis of identical markers).
- [x] All plots are contour plots with outliers or pseudocolor plots.
- [x] A numerical value for number of cells or percentage (with statistics) is provided.

Methodology

Sample preparation	Cells analysed by flow cytometry were either fresh mouse bone marrow, spleen, or peripheral blood or PBMCs or BMMCs derived from healthy donor or AML patient samples. blood was first processed using density gradient centrifugation using Lymphoprep or Panoll. Then, based on the experimental set up, cells were harvested according to the cell type, counted using a haemocytometer to examine cell count. Cell viability was analysed using the LIVE/DEAD fixable dead cell stain kit (molecular Probes) or 7-AAD (BD Biosciences). Following 20min incubation on ice, the cells were washed twice in FACs buffer. For cells

isolated from the BM, spleen or PB of mice, Fc receptor blockade (1:25) (Miltenyi Biotec, Germany) was performed for 15-20 mins on ice prior to staining. Cells were washed twice with ice-cold 1x PBS after staining and re-suspended in 250µL 1x PBS supplemented with 2% FCS prior to analysis. For intracellular cytokine staining, cells were treated with 1µL/mL of Brefeldin A (GolgiPlug) (BD Biosciences, Germany) in cRPMI-1640 medium for 4 h prior to staining using the BD Cytofix/Cytoperm kit (BD Biosciences, Germany) according to manufacturer's instructions. For cell surface staining, cells were isolated and washed twice with ice-cold 1x PBS prior to staining with the relevant antibodies for 30 mins on ice, in the dark.

All details are also provided in the manuscript.

Instrument

The majority of data were acquired on a BD LSR Fortessa or BD FACS CANTO II flow cytometer (BD Bioscience) and BD Fusion for cell sorting.

Software

The majority of data were analyzed using FlowJo (Flowjo 10.4 or 10.6, LLC) software.

Cell population abundance

The gating strategy was determined using unstained controls, single stains and fluorescence minus one (FMO) controls.

Gating strategy

All experiments were performed using appropiated controls and compensation controls were also considered. Cells were first gated using SSC-A vs FSC-A, doublet cells were excluded by gating on FSC-H vs FSC-A, dead cells were exclude by gating on AmCyan, 7-AAD, followed by donor/recipient cells discrimination gating using the SSC-A vs donor genotype mice. Subsequently, the desired paramiters were gated. Unstained controls, single stains and fluorescence minos one (FMO) controls were used for appropiate gating on desired parameters and differentiation between positive and negative cells. Gating strategy will be provided upon request.

☒ Tick this box to confirm that a figure exemplifying the gating strategy is provided in the Supplementary Information.
